# A phylogenomic and molecular signature based approach for characterization of the phylum Spirochaetes and its major clades: proposal for a taxonomic revision of the phylum

**DOI:** 10.3389/fmicb.2013.00217

**Published:** 2013-07-30

**Authors:** Radhey S. Gupta, Sharmeen Mahmood, Mobolaji Adeolu

**Affiliations:** Department of Biochemistry and Biomedical Sciences, McMaster UniversityHamilton, ON, Canada

**Keywords:** Spirochaetes, Spirochaetes phylogeny and taxonomy, molecular signatures, *Spirochaetaceae*, *Borreliaceae*, *Brachyspiriales*, *Leptospiriales*, conserved signature indels

## Abstract

The Spirochaetes species cause many important diseases including syphilis and Lyme disease. Except for their containing a distinctive endoflagella, no other molecular or biochemical characteristics are presently known that are specific for either all Spirochaetes or its different families. We report detailed comparative and phylogenomic analyses of protein sequences from Spirochaetes genomes to understand their evolutionary relationships and to identify molecular signatures for this group. These studies have identified 38 conserved signature indels (CSIs) that are specific for either all members of the phylum Spirochaetes or its different main clades. Of these CSIs, a 3 aa insert in the FlgC protein is uniquely shared by all sequenced Spirochaetes providing a molecular marker for this phylum. Seven, six, and five CSIs in different proteins are specific for members of the families *Spirochaetaceae*, *Brachyspiraceae*, and *Leptospiraceae*, respectively. Of the 19 other identified CSIs, 3 are uniquely shared by members of the genera *Sphaerochaeta*, *Spirochaeta*, and *Treponema*, whereas 16 others are specific for the genus *Borrelia*. A monophyletic grouping of the genera *Sphaerochaeta*, *Spirochaeta*, and *Treponema* distinct from the genus *Borrelia* is also strongly supported by phylogenetic trees based upon concatenated sequences of 22 conserved proteins. The molecular markers described here provide novel and more definitive means for identification and demarcation of different main groups of Spirochaetes. To accommodate the extensive genetic diversity of the Spirochaetes as revealed by different CSIs and phylogenetic analyses, it is proposed that the four families of this phylum should be elevated to the order level taxonomic ranks (viz. *Spirochaetales*, *Brevinematales* ord. nov., *Brachyspiriales* ord. nov., and *Leptospiriales* ord. nov.). It is further proposed that the genera *Borrelia* and *Cristispira* be transferred to a new family *Borreliaceae* fam. nov. within the order *Spirochaetales*.

## Introduction

The phylum Spirochaetes consists of a large group of motile bacteria which are widespread in the environment and are highly prevalent disease causing agents (Seshadri et al., 2004; Paster, 2011a). The members of this phylum share a distinguishing morphological feature, the endoflagella, a special class of flagella that folds back into the cell and remains within the periplasm (Li et al., 2008). Most spirochetes have one or more of these structures protruding from either pole of the cell, forming an axial filament, which gives rise to the characteristic jerky, corkscrew-like motility of the members of the phylum (Li et al., 2008; Paster, 2011a).Currently, the phylum Spirochaetes consists of 15 genera which are highly divergent in terms of their lifestyle and other characteristics (Euzéby, 2013). They live in marine sediments, deep within soil, commensally in the gut of arthropods, including termites, as well as in vertebrates as obligate parasites. They can also be free-living or host-associated, pathogenic or non-pathogenic, and aerobic or anaerobic (Paster, 2011a). There is also enormous variability in the genome sizes and organization of Spirochaetes species Table 1. However, despite the diverse characteristics of its members, the phylum Spirochaetes is currently comprised of a single class, *Spirochaetia*, containing a single order, *Spirochaetales*, which is made up of four families (viz. *Spirochaetaceae*, *Brachyspiraceae*, *Leptospiraceae*, and *Brevinemataceae*) (Paster, 2011a; Euzéby, 2013).

**Table 1:** **Genome characteristics of the sequenced members of the phylum Spirochaetes**.

**Strain name**	**Accession number**	**Size (Mb)**	**GC %**	**Chromosomes**	**Plasmids**	**Genome source**
*Borrelia afzelii* PKo	NC_017238	1.4	27.90	1	17	Casjens et al., 2011
*Borrelia bissettii* DN127	NC_015921	1.4	28.33	1	16	Schutzer et al., 2012
*Borrelia burgdorferi* B31^T^	NC_001318	1.52	28.18	1	21	Zhong and Barbour, 2004
*Borrelia crocidurae* Achema	NC_017808	1.53	29.06	1	39	Elbir et al., 2012
*Borrelia duttonii* Ly	NC_011229	1.57	28.02	1	16	Lescot et al., 2008
*Borrelia garinii* PBi	NC_006156	0.99	28.12	1	11	Glöckner et al., 2004
*Borrelia hermsii* DAH	NC_010673	0.93	29.81	1	2	Dai et al., 2006
*Borrelia recurrentis* A1	NC_011244	1.24	27.51	1	7	Unité des Rickettsies^1^
*Borrelia* sp. SV1	NZ_ABJZ00000000	1.28	28.27	1	9	Casjens et al., 2011
*Borrelia spielmanii* A14S	NZ_ABKB00000000	1.25	27.69	–	8	Schutzer et al., 2012
*Borrelia turicatae* 91E135	NC_008710	0.92	29.10	1	–	Rocky Mountain Laboratories^2^
*Borrelia valaisiana* VS116^T^	NZ_ABCY00000000	0.35	25.83	–	11	Schutzer et al., 2012
*Brachyspira hyodysenteriae* ATCC 27164^T^	NZ_ARSY00000000	3.05	27.00	1	1	DOE-JGI^3^
*Brachyspira intermedia* PWS/A^T^	NC_017243	3.31	27.19	1	1	Håfström et al., 2011
*Brachyspira murdochii* DSM 12563^T^	NC_014150	3.24	27.80	1	–	Pati et al., 2010
*Brachyspira pilosicoli* P43/6/78^T^	NC_019908	2.56	27.90	1	–	Lin et al., 2013
*Leptonema illini* DSM 21528^T^	NZ_AHKT00000000	4.52	54.30	–	–	DOE-JGI^3^
*Leptospira biflexa* Patoc 1 (Ames)^T^	NC_010842	3.96	38.90	2	1	Picardeau et al., 2008
*Leptospira borgpetersenii* L550	NC_008509	3.93	40.20	2	–	Bulach et al., 2006
*Leptospira broomii* 5399^T^	NZ_AHMO00000000	4.49	42.90	–	–	JCV^4^
*Leptospira inadai* 10^T^	NZ_AHMM00000000	4.57	44.50	–	–	JCV^4^
*Leptospira interrogans* RGA^T^	NZ_AOVR00000000	4.6	35.00	2	–	JCV^4^
*Leptospira kirschneri* 3522 C^T^	NZ_AHMN00000000	4.4	35.90	–	–	JCV^4^
*Leptospira kmetyi* Bejo-Iso9^T^	NZ_AHMP00000000	4.48	44.70	–	–	JCV^4^
*Leptospira licerasiae* VAR 010^T^	NZ_AHOO00000000	4.21	35.90	–	–	JCV^4^
*Leptospira meyeri* Went 5	NZ_AKXE00000000	4.19	38.00	–	–	JCV^4^
*Leptospira santarosai* LT 821^T^	NZ_ADOR00000000	3.88	41.80	–	–	Chou et al., 2012
*Leptospira* sp. Fiocruz LV3954	NZ_AKWV00000000	4.04	41.70	–	–	JCV^4^
*Leptospira weilii* 2006001853	NZ_AFLV00000000	4.37	40.80	–	–	JCV^4^
*Sphaerochaeta coccoides* DSM 17374^T^	NC_015436	2.23	50.60	1	–	Abt et al., 2012
*Sphaerochaeta globosa* Buddy^T^	NC_015152	3.32	48.90	1	–	DOE-JGI^3^
*Sphaerochaeta pleomorpha* Grapes^T^	NC_016633	3.59	46.20	1	–	DOE-JGI^3^
*Spirochaeta africana* DSM 8902^T^	NC_017098	3.29	57.80	1	–	DOE-JGI^3^
*Spirochaeta smaragdinae* DSM 11293^T^	NC_014364	4.65	49.00	1	–	Mavromatis et al., 2010
*Spirochaeta thermophila* DSM 6578^T^	NC_017583	2.56	60.90	1	–	DOE-JGI^3^
*Treponema azotonutricium* ZAS-9^T^	NC_015577	3.86	49.80	1	–	JCV^4^
*Treponema brennaborense* DSM 12168^T^	NC_015500	3.06	51.50	1	–	DOE-JGI^3^
*Treponema caldaria* DSM 7334^T^	NC_015732	3.24	45.60	1	–	Abt et al., 2013
*Treponema denticola* ATCC 35405^T^	NC_002967	2.84	37.90	1	1	Seshadri et al., 2004
*Treponema pallidum* Nichols	NC_000919	1.14	52.80	1	–	Fraser et al., 1997
*Treponema paraluiscuniculi* Cuniculi A	NC_015714	1.13	52.70	–	–	Smajs et al., 2011
*Treponema phagedenis* F0421	NZ_AEFH00000000	2.83	40.10	–	–	WUGSC^5^
*Treponema primitia* ZAS-2^T^	NC_015578	4.06	50.80	1	–	JCV^4^
*Treponema saccharophilum* DSM 2985^T^	NZ_AGRW00000000	3.45	53.20	–	–	DOE-JGI^3^
*Treponema* sp. JC4	NZ_AJGU00000000	3.03	40.30	–	–	CSIRO^6^
*Treponema succinifaciens* DSM 2489^T^	NC_015385	2.9	39.17	1	1	Han et al., 2011
*Treponema vincentii* ATCC 35580	NZ_ACYH00000000	2.51	45.70	–	–	JCV^4^
*Turneriella parva* DSM 21527^T^	NC_018020	4.41	53.60	1	1	DOE-JGI^3^

Genomic information was collected from: http://www.ncbi.nlm.nih.gov/genomes/

1 *Unité des Rickettsies: Genome sequenced by Unité des Rickettsies at Center National de Référence*.

2 *Rocky Mountain Laboratories: Genome sequenced by the Laboratory of Human Bacterial Pathogenesis at Rocky Mountain Laboratories*.

3 *DOE-JGI: Genome sequenced by the United States Department of Energy Joint Genome Institute*.

4 *JCV: Genome sequenced by the J. Craig Venter Institute*.

5 *WUGSC: Genome sequenced by the Washington University Genome Sequencing Center*.

6 *CSIRO: Genome sequenced by the Commonwealth Scientific and Industrial Research Organization*.

T *Type strain*.

There are four clinically important genera of the phylum Spirochaetes whose species are the causative agents of many globally prevalent illnesses, *Treponema*, *Borrelia*, *Leptospira*, and *Brachyspira* (Bellgard et al., 2009). Of these, *Treponema* and *Borrelia* are members of the family *Spirochaetaceae*, which also includes the genera *Clevelandina, Cristispira, Diplocalyx, Hollandina, Pillotina, Spirochaeta*, and *Sphaerochaeta* (Paster, 2011b; Euzéby, 2013). However, the genera *Clevelandina, Diplocalyx, Hollandina*, and *Pillotina* have yet to be isolated and grown in pure or mixed culture and their phylogeny is based largely on analyses of morphological characteristics (Bermudes et al., 1988). *Treponema pallidum* subspecies *pallidum* is the causative agent of syphilis, a sexually transmitted disease which affects at least 25 million adults worldwide (Gerbase et al., 1998). Other members of the genus *Treponema* are responsible for diseases such bejel, yaws, and pinta and play important role in periodontal diseases (Ellen and Galimanas, 2005; Visser and Ellen, 2011; Smajs et al., 2012). Members of the genus *Borrelia*, namely *Borrelia burgdorferi s* and *Borrelia recurrentis*, are important human pathogens that cause Lyme disease and relapsing fever, respectively (Dworkin et al., 2008; Nau et al., 2009; Cutler, 2010). *Leptospira* and *Brachyspira*, are members of the families *Leptospiraceae* and *Brachyspiraceae*, and causative agents of the diseases leptospirosis and intestinal spirochaetosis, respectively (Adler and de la Peña Moctezuma, 2010; Anthony et al., 2013; Euzéby, 2013).

Despite the importance of species of the phylum Spirochaetes in causing many important human diseases, the evolutionary relationship of species within this phylum remains poorly understood and no distinguishing molecular features are known that are specific for all members of the different families (Olsen et al., 2000; Paster and Dewhirst, 2000; Paster, 2011a). The availability of genome sequences provides a valuable resource to identify/discover novel molecular markers that are helpful in these regards and to gain insights into their evolutionary relationships. Genomes from 48 species covering the three main families of the phylum Spirochaetes are now available in the NCBI database (Table 1) (NCBI, 2013). The availability of genome sequences allows for the use of comparative genomic approaches to identify molecular markers that are specific for different bacterial taxa at various taxonomic levels. Using genomic sequences, one useful approach pioneered by our lab involves the discovery of Conserved Signature insertions/deletions (i.e., Indels) or CSIs present in protein sequences that are specific for different groups of organisms. Due to the specificity of these CSIs for particular groups/taxa of species, they provide valuable molecular markers of common evolutionary descent (i.e., synapomorphies) for identification and demarcation of different phylogenetic/taxonomic clades of organisms in molecular terms. Additionally, based upon the presence or absence of these CSIs in outgroup species, it is possible to infer whether the observed genetic change is an insert or a deletion and a rooted phylogenetic relationship among different groups can be derived (Baldauf and Palmer, 1993; Gupta, 1998; Griffiths and Gupta, 2004; Gao and Gupta, 2012a).

In this work, we report the results of comparative analyses on protein sequences for the phylum Spirochaetes to identify molecular markers (CSIs) that are specific for the species from the phylum and its subgroups, or those that provide information regarding interrelationships among them. These studies have led to identification of 38 CSIs providing novel molecular markers for the species from the phylum and clarifying their evolutionary relationships. Additionally, we have also constructed a phylogenetic tree for all genome sequenced members of the phylum Spirochaetes based upon concatenated sequences for 22 conserved proteins. The inferences from different identified CSIs are strongly supported by the branching pattern of species in the phylogenetic tree indicating that the identified CSIs provide reliable molecular markers for the indicated groups of Spirochaetes.

## Methods

### Phylogenetic sequence analysis

Phylogenetic analysis was performed on a concatenated sequence alignment of 22 highly conserved proteins (viz. UvrD, GyrA, GyrB, RpoB, RpoC, EF-G, EF-Tu, RecA, ArgRS, IleRS, ThrRS, TrpRS, SecY, DnaK, and ribosomal proteins L2, L5, S2, S3, and S9) which have been widely used for phylogenetic analysis (Harris et al., 2003; Gao and Gupta, 2012a). Sequences for these proteins were obtained from the NCBI database for representative strains of all the sequenced Spirochaetes species (Table 1) and *Thermosynechococcus elongatus* and *Nostoc flagelliforme* which were used to root the tree. Multiple sequence alignments for these proteins were created using Clustal_X 1.83 (Jeanmougin et al., 1998) and concatenated into a single alignment file. Poorly aligned regions from this alignment file were removed using Gblocks 0.91 b (Castresana, 2000). The resulting alignment, which contained 7411 aligned amino acids, was used for phylogenetic analysis. The maximum likelihood (ML) and neighbor joining (NJ) trees based on 100 bootstrap replicates of this alignment were constructed using MEGA 5.1 (Tamura et al., 2011) employing the Whelan and Goldman (Whelan and Goldman, 2001) and Jones-Taylor-Thornton (Jones et al., 1992) substitution models, respectively.

A 16S rRNA gene sequence tree was also created for 107 sequences that included representative species for all 11 cultured Spirochaetes genera. 16S rRNA gene sequences larger than 1200 bp were obtained for all type species classified under the phylum Spirochaetes in release 114 of the SILVA database (Quast et al., 2013). Information for these sequences is provided in Supplemental Table 1. A ML tree based on these sequences was created using 100 bootstrap replicates of the 16S rRNA sequence alignments in MEGA 5.1 (Tamura et al., 2011) employing the General Time-Reversible (Tavaré, 1986) substitution model.

### Identification of molecular markers (CSIs)

To identify CSIs that are commonly shared by different groups of Spirochaetes, BLASTp searches (Altschul et al., 1997) were performed on each protein in the genome of *Treponema pallidum* subspecies *pallidum* strain Nichols. These searches were performed using the default BLAST parameters against all available sequences in the GenBank non-redundant database. For those proteins for whom high scoring homologs (*E*-values < 1e^−20^) were present in other species from the phylum Spirochaetes and some other bacterial groups multiple sequence alignments were created using the Clustal_X 1.83 program (Jeanmougin et al., 1998). These alignments were visually inspected for the presence of insertions or deletions that were flanked on both sides by at least 4–5 conserved amino acid residues in the neighboring 30–40 amino acids. Indels that were not flanked by conserved regions were not further considered, as they do not provide useful molecular markers (Gupta, 1998; Gao and Gupta, 2012a; Adeolu and Gupta, 2013). The specificity of potentially useful indels for members of the Spirochaetes was further evaluated by carrying out detailed Blastp searches on short sequence segments containing the indel and the flanking conserved regions (60–100 amino acids long). To ensure that the identified signatures are only present in the Spirochaetes homologs, a minimum of 250 blast hits with the highest similarity to the query sequence were examined for the presence or absence of these CSIs. In this work, we report the results of only those CSIs that are specific for different groups of Spirochaetes and where similar CSIs were not observed in any other bacteria in the top 250 blast hits. The sequence alignment files presented here contain sequence information for all sequenced genera within Spirochaetes. However, due to size restraints, different strains and/or species of the sequenced genera are not shown as they all exhibited similar patterns.

## Results

### Genomic characteristics of the sequenced spirochaetes

There are currently 48 genome sequenced species of Spirochaetes. Table 1 lists some characteristics of representative strains for all Spirochaetes species that have been completely sequenced. The genome sizes of these species of Spirochaetes showed a large amount of variation, ranging from 0.92 to 4.7 Mb in length. The G + C content of these species also showed a large amount of variation, ranging from 25.8 to 60.9%. The members of the phylum Spirochaetes also exhibited a large amount of variation in genome structure. The genome structure of members of genus *Borrelia* is one of the most unique among prokaryotes (Chaconas, 2005; Chaconas and Kobryn, 2010). The *Borrelia* genome consists of 6–24 DNA segments, including a linear chromosome about 900 kb in length which is accompanied by multiple essential linear and circular plasmids ranging from 5 to 220 kb in length (Chaconas and Kobryn, 2010). Linear chromosomes and plasmids terminated by covalently closed hairpin telomers are particularly uncommon genomic features among prokaryotes and are only found in the genomes of the *Borrelia* species and the species *Agrobacterium tumefaciens* (Goodner et al., 2001; Kobryn, 2007; Chaconas and Kobryn, 2010). Members of the genus *Leptospira* also have an unusual genome structure consisting of two circular chromosomes, a big chromosome about 3.6–4.2 Mb in length and a smaller chromosome about 300 kb in length (Ren et al., 2003; Picardeau et al., 2008).

### Phylogenetic analyses of the sequenced spirochaetes

**Figure 1:**
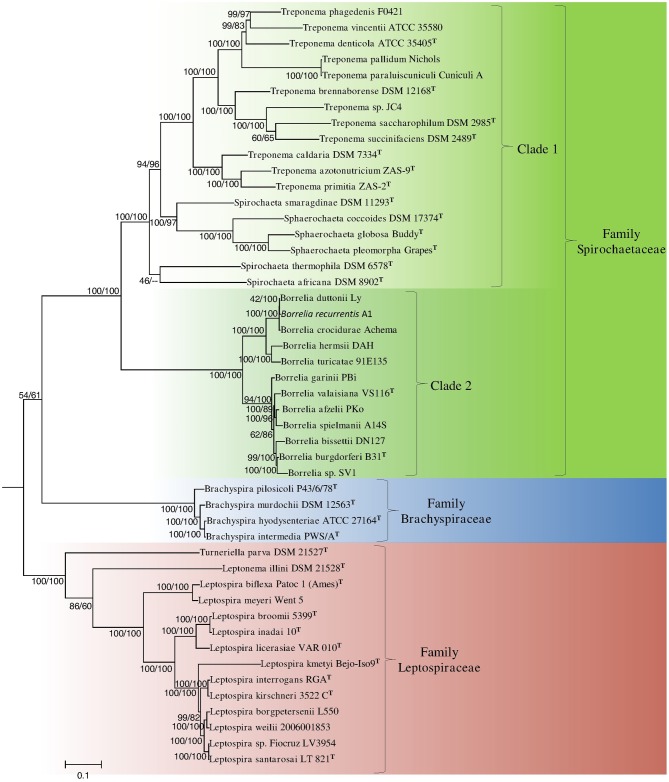
**A phylogenetic tree of genome sequenced members of the phylum Spirochaetes based on the concatenated amino acid sequences of 22 conserved proteins**. The tree shown is a maximum-likelihood (ML) distance tree. Bootstrap values are shown at branch nodes for both maximum-likelihood and neighbor-joining tree construction methods as ML/NJ. The different sequenced families and two main clades of the family *Spirochaetaceae* supported by the tree are marked. The letter ^T^ refers to the type strain of the species.

**Figure 2:**
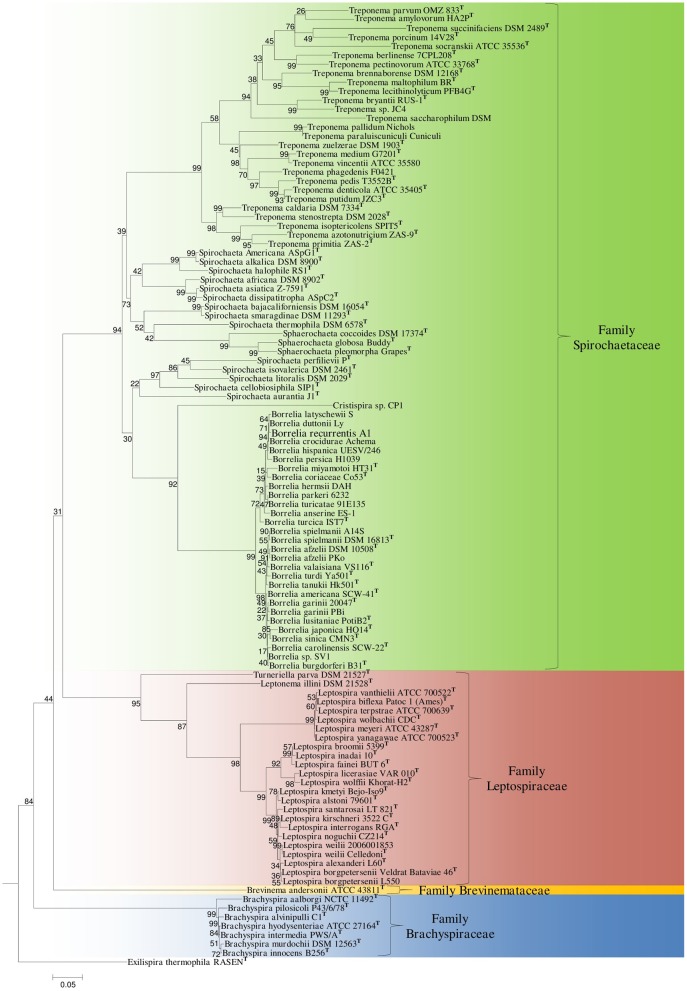
**A ML tree based on the 16S rRNA gene sequences of representative species from cultured genera within the phylum Spirochaetes**. Bootstrap values are shown at branch nodes. The different families of the phylum Spirochaetes are marked. The letter^T^ refers to the type strain of the species. The accession numbers of the 16S rRNA gene sequences used in this analysis are provided in Supplemental Table 1.

The branching order of species within the phylum Spirochaetes has primarily been determined using 16S rRNA sequence based phylogenetic trees (Paster and Dewhirst, 2000; Paster, 2011a). In these trees, the four families with the phylum branch into distinct monophyletic clades separated by long branches. However, the interrelationships of members of the family *Spirochaetaceae* are not reliably resolved (Paster, 2011b) (Figure 2). Phylogenetic trees derived from large numbers of conserved genes/proteins provide greater resolving power than those based on any single gene or protein (Rokas et al., 2003; Ciccarelli et al., 2006; Wu et al., 2009; Gao and Gupta, 2012a). In this study, we have constructed phylogenetic trees of the genome sequenced members of the phylum Spirochaetes listed in Table 1 using 22 conserved housekeeping and ribosomal proteins. The trees were constructed using both the NJ and ML methodologies and branching patterns generated by both methodologies were highly similar (Figure 1).

In the concatenated protein trees, which are rooted using the species *T. elongatus* and *N. flagelliforme*, the members of the three sequenced families of Spirochaetes (viz. *Spirochaetaceae*, *Brachyspiraceae*, and *Leptospiraceae*) formed three distinct monophyletic clades (Figure 1). Additionally, the branching order of members of the family *Spirochaetaceae* is well-resolved in the concatenated protein trees. Within the *Spirochaetaceae* clade, the genera *Treponema*, *Spirochaeta*, and *Sphaerochaeta* formed a well-supported monophyletic clade separated from the members of the genus *Borrelia* by a long branch. The *Treponema*, *Spirochaeta*, and *Sphaerochaeta* clade exhibited a large amount of diversity and consisted of a number of strongly supported subclades. Members of each of the sequenced genera within Spirochaetes formed monophyletic clusters with the exception of the genus *Spirochaeta*, where *Spirochaeta smaragdinae* branched with the genus *Sphaerochaeta*. Another *Spirochaeta* species, *S. caldaria*, which branched within the *Treponema* has recently been reclassified as *Treponema caldaria* (Abt et al., 2013). The remaining *Spirochaeta* (viz. *S*. *thermophila* and *S. africana*) branched deeply within the *Treponema*, *Spirochaeta*, and *Sphaerochaeta* clade (Figure 1). The monophyletic clade containing all the members of the genus *Borrelia* consisted of two highly distinct subclades, one containing *Borrelia burgdorferi*, and related species of *Borrelia* and the other containing *Borrelia recurrentis* related species.

The 16S rRNA tree shown in Figure 2 includes all of the members included in the concatenated protein tree as well as other cultured members of the phylum Spirochaetes which have yet to be genome sequenced. The branching patterns in the 16S rRNA phylogenetic tree were similar to those observed in the concatenated protein tree; all families within the phylum branched distinctly. Within the cluster consisting of members of the family *Spirochaetaceae* the genera *Treponema*, *Sphaerochaeta*, and most members of the genus *Spirochaeta* formed a monophyletic clade. The genera *Borrelia* and *Cristispira* also formed a well-supported monophyletic clade that was distinct from the genera *Treponema*, *Spirochaeta*, and *Sphaerochaeta* within the *Spirochaetaceae* clade. The different sequenced members of the genus *Borrelia* also formed two distinct clusters in the 16S rRNA tree (Figure 2).

### CSI specific for the phylum spirochaetes

CSIs that are restricted to a group of related species are a novel class of molecular marker with high utility for evolutionary studies (Gupta, 1998; Rokas et al., 2003; Gupta, 2009; Gao and Gupta, 2012a). The co-occurrence of multiple CSIs in different species may be due to shared evolutionary history, convergent evolution, lateral gene transfer. However, the unique shared presence of multiple CSIs in a diverse range by a related group of species is most parsimoniously explained by the occurrence of the rare genetic changes that resulted in these CSIs in a common ancestor of the group, followed by vertical transmission of these CSIs to various descendant species (Gupta, 1998; Rokas and Holland, 2000; Gogarten et al., 2002; Gupta and Griffiths, 2002; Gao and Gupta, 2012a). Hence, these CSIs represent molecular synapomorphies of common evolutionary descent and they provide useful markers for identifying different groups of organisms in molecular terms and for understanding their interrelationships independently of phylogenetic trees (Gupta, 1998; Gupta and Griffiths, 2002; Gao and Gupta, 2012a,b). The CSI-based approach has recently been used to propose important taxonomic changes for a number of groups of bacteria (viz. Chloroflexi, *Coriobacateriia*, *Neisseriales*, and *Bacillus*) at different taxonomic ranks (Gupta et al., 2012, 2013; Adeolu and Gupta, 2013; Bhandari et al., 2013). In the present work, we have completed comprehensive genomic analyses to identify CSIs that are primarily restricted to the phylum Spirochaetes or its subgroups. Information regarding the species specificities of these CSIs and their evolutionary significances are discussed below.

**Figure 3:**
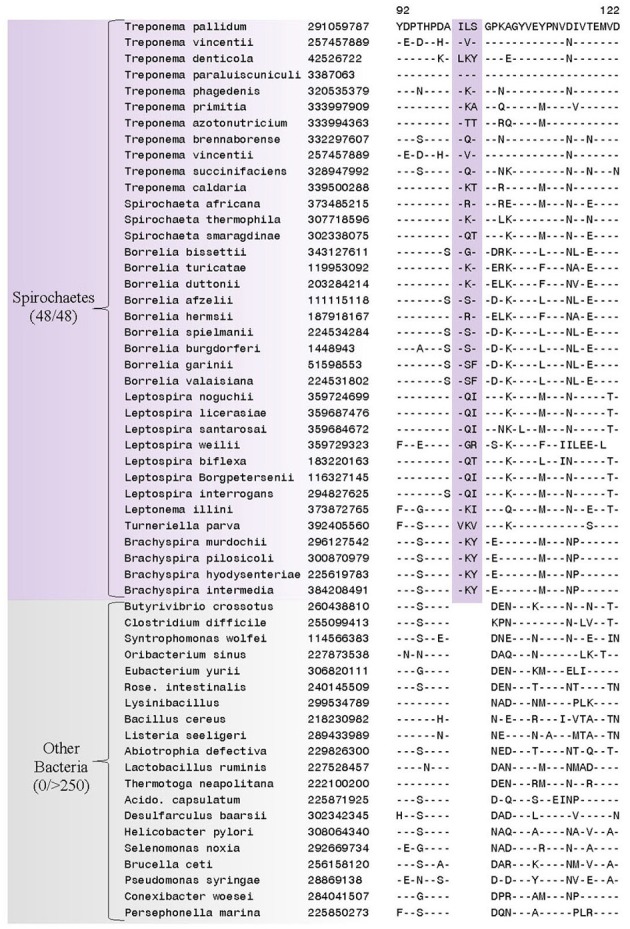
**A partial sequence alignment of the flagellar basal-body rod protein FlgC, showing a CSI (boxed) that is uniquely present in all members of the phylum Spirochaetes**. Sequence information for only a limited number of species from the Spirochaetes and other bacteria is shown here, but unless otherwise indicated similar CSIs were detected in all members of the indicated group and not detected in any other bacterial species in the top 250 Blastp hits. The dashes (−) in the alignments indicate identity with the residue in the top sequence. GenBank identification (GI) numbers for each sequence are indicated in the second column. Sequence homologs for this protein were not identified from members of the genus *Sphaerochaeta*.

Our analyses have identified 38 CSIs in diverse and important proteins that are specific for members of the Spirochaetes. One CSI has been identified that is specifically found in all of the sequenced members of the phylum Spirochaetes and not found in homologous proteins from any other bacterial species (in the top 250 Blast hits) (Figure 3). This CSI consists of a 3 amino acid (aa) insertion located in the flagellar basal-body rod protein FlgC, a component of the basal body which comprises a large portion of the flagella (Macnab, 2003). This CSI represents a unique molecular characteristic of the phylum Spirochaetes and may be related to the characteristic flagellar morphology shared by members of the phylum.

### CSIs that are specific for different families of spirochaetes

**Figure 4:**
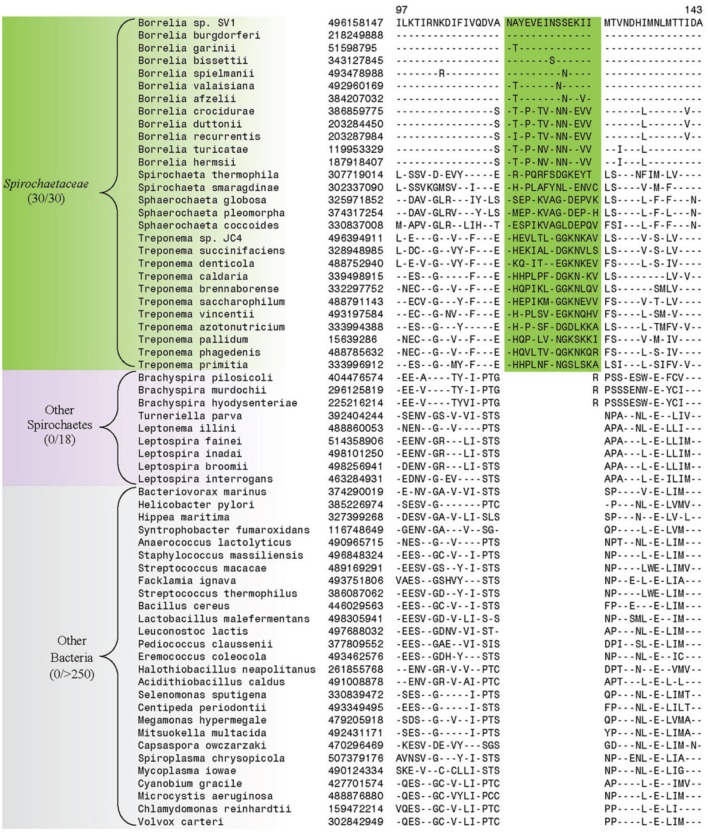
**A partial sequence alignment of the protein alanyl-tRNA synthetase showing a two amino acid insertion (boxed) identified in homologs from the family *Spirochaetaceae*, but not found in the sequence homologs of any other sequenced bacteria**. Sequence information for other *Spirochaetaceae* specific CSIs is presented in Supplemental Figures 3–6 and summarized in Table 2.

Many of the CSIs identified by our analyses are specific for the different sequenced families within the phylum Spirochaetes (viz. *Spirochaetaceae*, *Brachyspiraceae*, and *Leptospiraceae*) allowing us to demarcate these families in clear molecular terms. Seven of the CSIs identified by our analyses are specific for the family *Spirochaetaceae*. One example of a CSI that is specific for the species from the family *Spirochaetaceae* is a 15 aa insertion in a highly conserved region of the protein phosphoribosylpyrophosphate synthetase, which is uniquely found in all members of the family *Spirochaetaceae* but not in any other sequenced bacterial groups (Figure 4). Sequence information for 6 other CSIs in diverse proteins (viz. Alanyl-tRNA synthetase, phosphoribosylpyrophosphate synthetase, preprotein translocase SecY, peptide chain release factor 2, DNA mismatch repair protein MutS, and DNA mismatch repair protein MutL) that are also specifically present in members of the family *Spirochaetaceae* is presented in Supplementary Figures 1–6 and some of their characteristics are summarized in Table 2.

**Table 2:** **Conserved signature Indels that are specific for members of the family *Spirochaetaceae***.

**Protein name**	**Gene name**	**GI number**	**Figure number**	**Indel size**	**Indel position**
Phosphoribosylpyrophosphate synthetase	prsA	496158147	Figure 4	15 aa ins	97–143
Alanyl-tRNA synthetase	alaS	386859446	Supplemental Figure 1	2 aa ins	277–306
Phosphoribosylpyrophosphate synthetase	prsA	387827445	Supplemental Figure 2	8 aa ins	256–297
Preprotein translocase	secY	15639201	Supplemental Figure 3	1 aa del	340–373
Peptide chain release factor 2	prfB	257457828	Supplemental Figure 4	1 aa del	137–176
DNA mismatch repair protein MutS	mutS	224532424	Supplemental Figure 5	2 aa del	720–751
DNA mismatch repair protein MutL	mutL	338706271	Supplemental Figure 6	4 aa del	494–520

**Figure 5:**
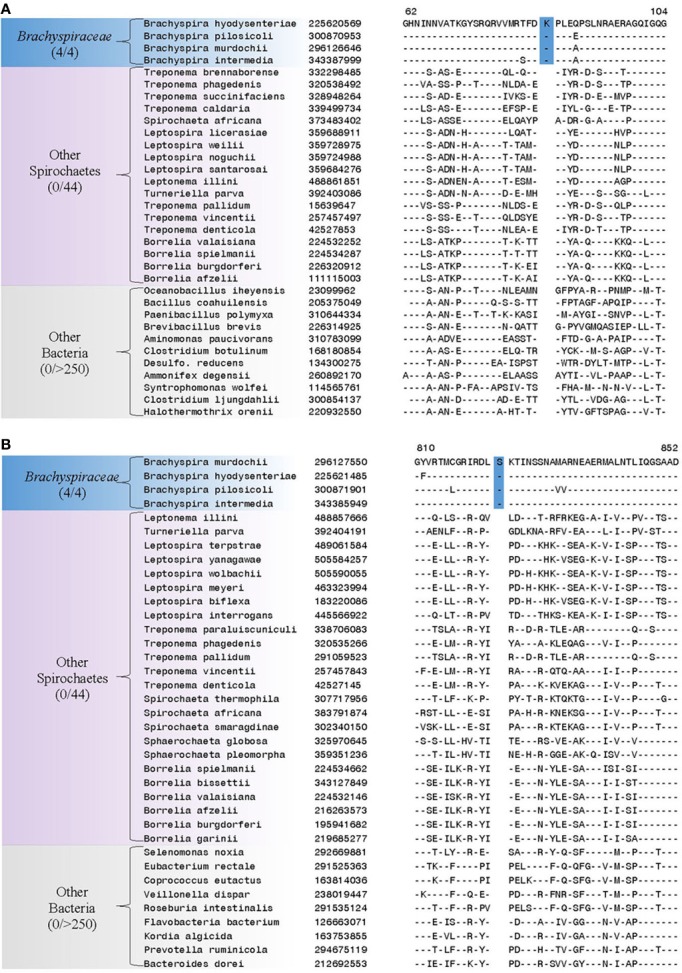
**Partial sequence alignments of (A) Flagellar hook-associated protein FlgK and (B) DNA polymerase I, showing two CSIs that are specific for the family *Brachyspiraceae*, but not found in the sequence homologs of any other sequenced bacteria**. Sequence homologs for flagellar hook-associated protein FlgK were not identified from members of the genus *Sphaerochaeta*. Sequence information for other *Brachyspiraceae* specific CSIs is presented in Supplemental Figures 7–10 and summarized in Table 3.

Our analyses have also identified 6 CSIs in diverse proteins that are specifically found in members of the family *Brachyspiraceae* and absent in all other bacterial groups. One of these *Brachyspiraceae*-specific CSIs, a 1 aa insertion, is present in the flagellar hook-associated protein FlgK, a protein involved in flagellar hook morphogenesis (Figure 5A) (Homma et al., 1990). Another *Brachyspiraceae*-specific CSI, a 1 aa insertion, is found in a highly conserved region of DNA polymerase I (Figure 5B). These proteins represent highly conserved and essential components of members of the family *Brachyspiraceae* which contain conserved molecular changes not found in any other sequenced bacterial group. Sequence information for 4 other CSIs in three other proteins (viz. valyl-tRNA synthetase, ATP-dependent protease La, and glutamyl-tRNA amidotransferase subunit B) that are also specifically present in members of the family *Brachyspiraceae* is presented in Supplemental Figures 7–10 and some of their characteristics are summarized in Table 3.

**Table 3:** **Conserved signature Indels that are specific for members of the family *Brachyspiraceae***.

**Protein name**	**Gene name**	**GI number**	**Figure number**	**Indel size**	**Indel position**
Flagellar hook-associated protein FlgK	flgK	225620569	Figure 5A	1 aa ins	62–104
DNA polymerase I	polA	296127550	Figure 5B	1 aa ins	810–852
Valyl-tRNA synthetase	valS	300871449	Supplemental Figure 7	1 aa ins	225–263
Valyl-tRNA synthetase	valS	300871449	Supplemental Figure 8	2 aa del	660–703
ATP-dependent protease La	lon	225620632	Supplemental Figure 9	1 aa ins	760–793
Glutamyl-tRNA amidotransferase subunit B	gatB	300871379	Supplemental Figure 10	1 aa ins	325–361

**Figure 6:**
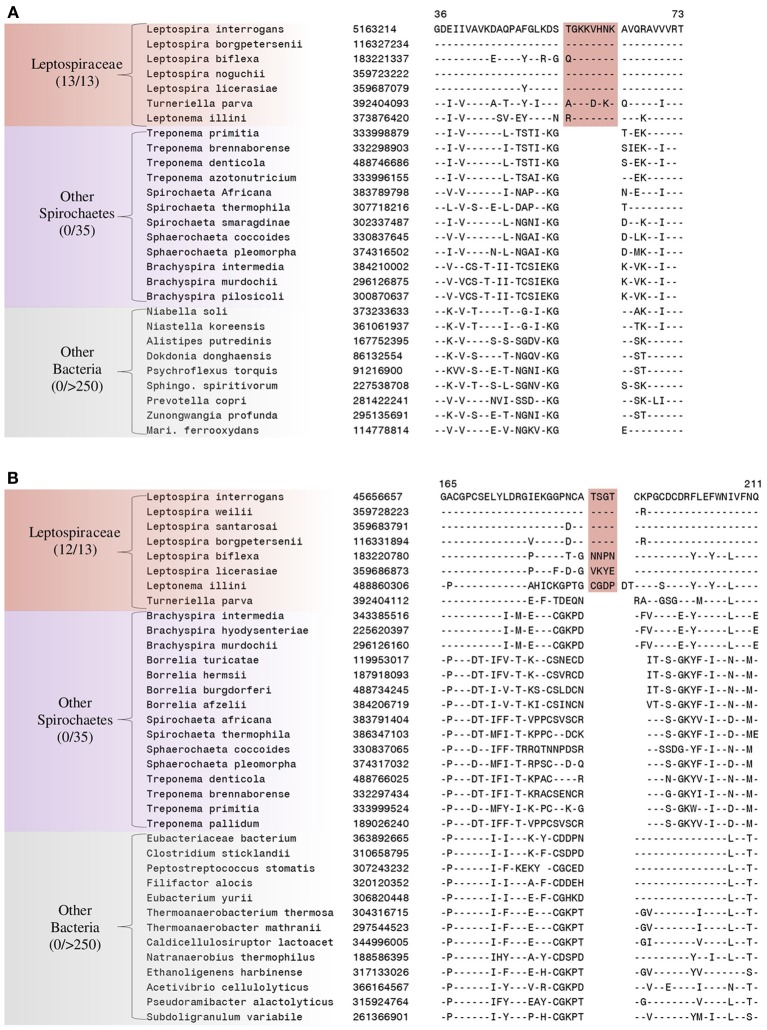
**Partial sequence alignments of (A) 50S Ribosomal protein L14 and (B) Alanyl-tRNA synthetase, showing two CSIs that are specific for the family *Leptospiraceae*, but not found in the sequence homologs of any other sequenced bacteria**. Sequence information for other *Leptospiraceae* specific CSIs is presented in Supplemental Figures 11–13 and summarized in Table 4.

We have also identified 5 CSIs that are uniquely present in members of the family *Leptospiraceae*. Two examples of such CSIs are shown in Figure 6. The first of these CSIs, an 8 aa insertion in the 50S ribosomal protein L14, is shown in Figure 6A, and the other CSI, a 4 aa insert in alanyl-tRNA synthetase, is shown in Figure 6B. Both of these CSIs are found in members of the the family *Leptospiraceae* and absent in every other sequenced bacterial group. Sequence information for 4 other CSIs in diverse proteins (viz. 30S Ribosomal protein S2, flagellar basal-body rod protein FlgG, and flagellar filament core protein FlaB) that are also specifically present in members of the family *Leptospiraceae* is presented in Supplemental Figures 11–14 and some of their characteristics are summarized in Table 4.

**Table 4:** **Conserved signature Indels that are specific for members of the family *Leptospiraceae***.

**Protein name**	**Gene name**	**GI number**	**Figure Number**	**Indel Size**	**Indel Position**
50S Ribosomal protein L14	rplN	5163214	Figure 6A	8 aa ins	36–73
Alanyl-tRNA synthetase	alaS	45656657	Figure 6B	4 aa ins	165–211
30S Ribosomal protein S2	rpsB	116330588	Supplemental Figure 11	2 aa ins	108–141
Flagellar filament core protein FlaB	flaB	12657818	Supplemental Figure 12	4 aa del	130–168
Flagellar basal-body rod protein FlgG	flgG	294828153	Supplemental Figure 13	1 aa ins	80–123

### CSIs distinguishing two clades within the family *spirochaetaceae*

**Figure 7:**
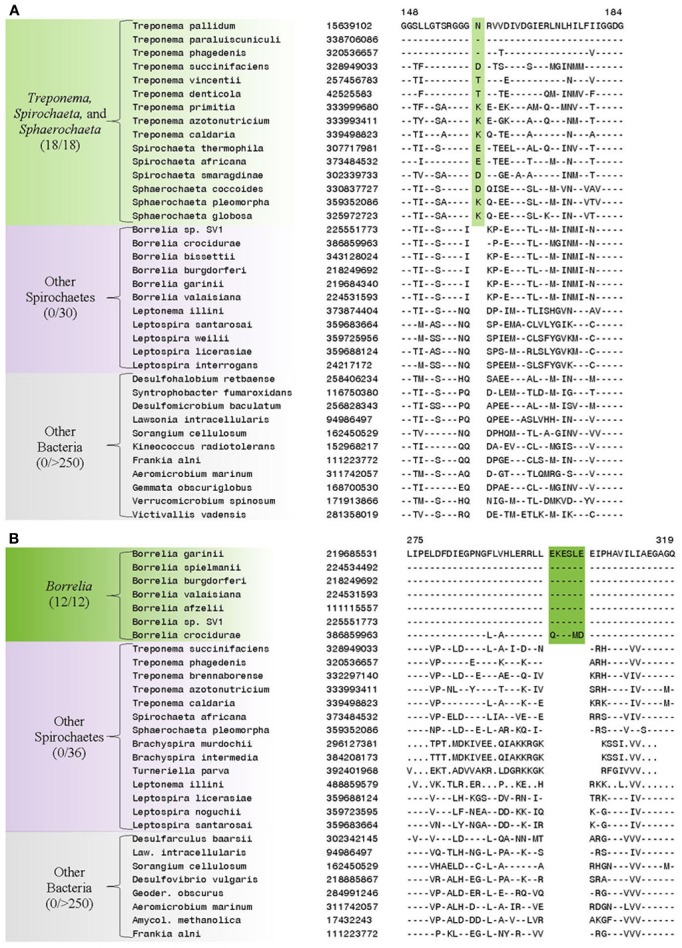
**(A)** Partial sequence alignment of the protein 6-phosphofructokinase (pyrophosphate) containing a 1 amino acid insert in a conserved region that is specifically present in the species from the genera *Treponema, Spirochaeta, and Sphaerochaeta*, but not found in any other sequenced bacteria. **(B)** Partial sequence alignment of phosphofructokinase containing a 6 amino acid insert that is specific for the genera *Borrelia*. Sequence information for other CSIs showing similar specificities is provided in Table 5 and in Supplemental Figures 14–30.

In addition to the numerous CSIs identified in our analyses for the sequenced families within the phylum Spirochaetes, we have also identified a number of CSIs that elucidate the relationship of the genera within the family *Spirochaetaceae*. Three of the identified CSIs are uniquely shared by the genera *Treponema*, *Spirochaeta*, and *Sphaerochaeta*. One example of a CSI specific to these three genera, a 1 aa deletion in the 30S ribosomal protein S13, a component of the protein translation complex, is shown in Figure 7A. Sequence information for 2 other CSIs specifically found in these three genera is provided in Table 5 and Supplemental Figures 14, 15. An additional 16 CSIs were uniquely shared by members of the genus *Borrelia*. One example of a CSI consisting of a 6 aa insertion in the glycolysis related protein, phosphofructokinase, that is specific to the members of the genus *Borrelia* is shown in Figure 7B. Fifteen other CSIs were also specifically found in members of the genus *Borrelia* and information for them is presented in Table 5 and Supplemental Figures 16–30.

**Table 5:** **Conserved Signature Indels that are specific for groups within the family *Spirochaetaceae***.

**Protein name**	**Gene name**	**GI Number**	**Figure Number**	**Specificity**	**Indel size**	**Indel position**
6-phosphofructokinase (pyrophosphate)	pfp	15639102	Figure 7A	*Treponema*, *Spirochaeta* and *Sphaerochaeta*	1 aa ins	148–184
Bifunctional Hpr kinase/phosphatase	hprK	3322886	Supplemental Figure 14	*Treponema*, *Spirochaeta* and *Sphaerochaeta*	1 aa ins	183–221
30S ribosomal protein S13	rpsM	302337499	Supplemental Figure 15	*Treponema*, *Spirochaeta* and *Sphaerochaeta*	1 aa del	1–39
Phosphofructokinase	pfk	219685531	Figure 7B	*Borrelia*	6 aa ins	275–319
50S ribosomal protein L4	rplD	224534698	Supplemental Figure 16	*Borrelia*	1 aa ins	103–136
tRNA pseudouridine 55 synthase	truB	203284699	Supplemental Figure 17	*Borrelia*	2 aa ins	143–178
Translation elongation factor Tu	tuf	203284386	Supplemental Figure 18	*Borrelia*	1 aa del	330–369
Histidyl-tRNA synthetase	hisS	187918014	Supplemental Figure 19	*Borrelia*	1 aa del	273–301
Seryl-tRNA synthetase	serS	187918098	Supplemental Figure 20	*Borrelia*	1 aa del	231–264
Spoiiij-associtated protein	jag	219684344	Supplemental Figure 21	*Borrelia*	3 aa ins	114–154
Nicotinate phosphoribosyltransferase	pncB	187918492	Supplemental Figure 22	*Borrelia*	1 aa del	134–159
Ribose 5-phosphate isomerase	rpiA	119953435	Supplemental Figure 23	*Borrelia*	1 aa ins	86–110
Ribonuclease Z	rnz	195941574	Supplemental Figure 24	*Borrelia*	2 aa ins	64–94
Hypothetical protein BGAFAR04_0762	–	386859948	Supplemental Figure 25	*Borrelia*	1 aa ins	206–236
Signal recognition particle, subunit FFH/SRP54	–	119953471	Supplemental Figure 26	*Borrelia*	1 aa ins	374–412
Hypothetical protein BSV1_0075	–	15594416	Supplemental Figure 27	*Borrelia*	1 aa del	52–97
Aspartyl/glutamyl-tRNA amidotransferase subunit A	gatA	119953137	Supplemental Figure 28	*Borrelia*	1 aa ins	364–402
Ribosomal RNA methyltransferase	rlmE	203284234	Supplemental Figure 29	*Borrelia*	1 aa ins	15–48
LysM domain/M23/M37 peptidase domain protein	–	224534310	Supplemental Figure 30	*Borrelia*	1 aa ins	320–365

## Discussion

**Figure 8:**
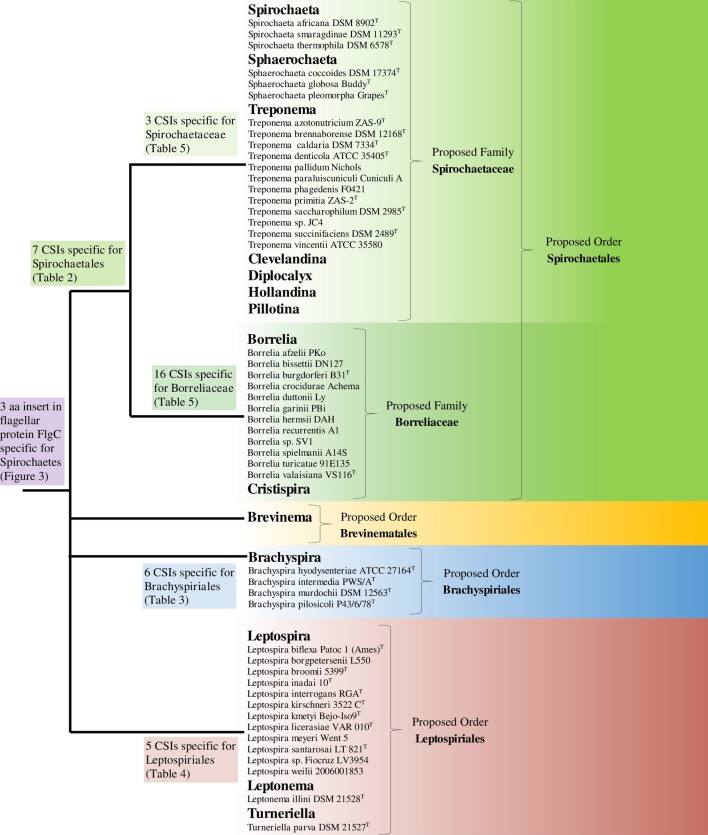
**A summary diagram depicting the distribution of identified CSIs and the proposed reclassification of the groups within the phylum Spirochaetes**. A representative strain is listed for each genome sequenced species. The letter ^T^ refers to the type strain of the species.

The phylum Spirochaetes is currently distinguished from other bacteria on the basis of both branching in 16S rRNA sequence based phylogenies and the presence of the endoflagella that characterizes the phylum (Paster, 2011a; Euzéby, 2013). Apart from the presence of endoflagella, no reliable morphological, biochemical, or molecular characteristics are known that are specifically shared by all members of the phylum. Additionally, the phylum contains four divergent lineages, contained within a single class/order, that are demarcated largely on the basis of 16S rRNA sequence based phylogenies (Paster, 2011a). In this work, we have utilized comparative genomic techniques to identify large numbers of novel molecular signatures (CSIs) that are distinctive characteristics of either all members of the phylum Spirochaetes or for its different subgroups at multiple phylogenetic levels and which can be used to demarcate these groups in more definitive molecular terms. A summary diagram depicting the species distribution of the identified CSIs is shown in Figure 8.

The phylum Spirochaetes is rare in having a defining morphological characteristic, the endoflagella, which correlates to the clustering of the members of the phylum in 16S rRNA phylogenetic trees (Ludwig and Klenk, 2001; Cavalier-Smith, 2002; Paster, 2011a). The endoflagella is a unique feature of the phylum and is thought to responsible for the great pathogenic and ecological diversity of its many members (Ren et al., 2003). Of the 38 CSIs we have identified in this study, one was uniquely shared by all 48 members of the phylum Spirochaetes and absent in every other sequenced group of bacteria. The identified CSI is located in the flagellar basal-body rod protein FlgC, a core component of the motor complex of the flagella (Macnab, 2003). This CSI provides a novel means to distinguish the members of the phylum from all other bacteria in molecular terms and provides another delimiting marker for the group in addition to the endoflagella. While the role of this CSI in the function or morphology of the Spirochaetes flagella is currently unknown, the unique presence of this CSI in a flagellar protein in all members of the phylum Spirochaetes suggests that it may be related to the unique flagella ultrastructure of the phylum. Earlier work has established that the CSIs are primarily located on surface loops of proteins which are important in protein-protein interactions (Akiva et al., 2008; Singh and Gupta, 2009; Gupta, 2010). Thus, the CSI identified in FlgC likely plays an important role in the cellular functions of the flagellar basal-body.

The phylum Spirochaetes contains 4 main lineages (viz. *Spirochaetaceae*, *Brachyspiraceae*, *Leptospiraceae*, and *Brevinemataceae*). These lineages have historically been distinguished from each other by their biochemical characteristics and their 16S rRNA gene sequences (Harwood and Canale-Parola, 1984; Paster et al., 1991; Paster, 2011a). In this study we have also identified 22 CSIs in a diverse range of proteins that are specific to each of the main sequenced lineages of the phylum Spirochaetes (viz. *Spirochaetaceae*, *Brachyspiraceae*, and *Leptospiraceae*), which serve to distinguish these lineages from themselves and all other bacteria. Seven of these identified CSIs were specific for the family *Spirochaetaceae*, 6 CSIs were identified that were specific for the family *Brachyspiraceae*, and 5 CSIs were identified that were specific to the family *Leptospiraceae*. Each of these lineages also branch distinctly and are separated by long branches in both 16S rRNA based and concatenated protein based phylogenetic trees (Figures 1, 2). This molecular and phylogenetic evidence supports the current division of these lineages. However, the large number of CSIs discovered for each of these groups and their genetic distances suggests that these lineages may represent higher taxonomic divisions (viz. orders or classes) than currently recognized. It is noteworthy that two of the CSIs that are specific for the *Brachyspiraceae* family and one that is specific for the *Leptospiraceae* are again found in flagella-related proteins (viz. FlgK, FlgB, FlgG) indicating that there might be interesting differences in the structures and/or functions of flagella within the Spirochaete families.

The family *Spirochaetaceae*, which contains the genera *Borrelia, Clevelandina, Cristispira, Diplocalyx, Hollandina, Pillotina, Sphaerochaeta, Spirochaeta*, and *Treponema*, is the most diverse of the lineages within the phylum Spirochaetes (Paster, 2011b; Euzéby, 2013). The interrelationships between the genera within this family are not reliably resolved by 16S rRNA sequence analysis (Paster, 2011b) (Figure 2). In this study we have identified 19 CSIs which serve to delineate at least certain relationships within the family *Spirochaetaceae*. Three of the CSIs identified are specifically found in members of the genera *Sphaerochaeta*, *Spirochaeta*, and *Treponema* and 16 additional CSIs were identified that are specifically found in members of the genus *Borrelia*. These CSIs suggest that the genera *Sphaerochaeta*, *Spirochaeta*, and *Treponema* shared a common ancestor distinct from the members of the genus *Borrelia*. In our concatenated protein phylogenetic tree, the genera *Sphaerochaeta*, *Spirochaeta* and *Treponema* formed a well-supported monophyletic clade, which was separated from the members of the genus *Borrelia* by a long branch, supporting the relationship delineated by these CSIs. Both of these two clades also exhibit considerable phylogenetic diversity. The clade consisting of genera *Sphaerochaeta*, *Spirochaeta*, and *Treponema* contains a number of distinct smaller subclades while the members of the genus *Borrelia* form two highly distinct clades in the phylogenetic trees. However, further work to identify molecular markers will be required to determine the significance of the branching of these subclades. The genus *Cristispira* has not had its genome sequenced, but it branches with the members of the genus *Borrelia* reliably in 16S rRNA based phylogenetic trees suggesting that some, if not all, of the *Borrelia* specific CSIs identified in this study may also be found in *Cristispira* (Paster, 2011b) (Figure 2). The remaining members of the family *Spirochaetaceae* (viz. *Clevelandina, Diplocalyx, Hollandina*, and *Pillotina*) have been identified in the hindguts of termite and cockroaches but have yet to be isolated and grown in pure or mixed culture. The current placement of the identified members of *Clevelandina, Diplocalyx, Hollandina*, and *Pillotina* in distinct genera within the family *Spirochaetaceae* is ambiguous and based largely on analyses of morphological characteristics (Bermudes et al., 1988). No genome or 16S rRNA sequences are currently available from these genera for phylogenetic analysis. However, the observations presented in this report suggest that the family *Spirochaetaceae* contains at least two distinct monophyletic groups: one consisting of the genera *Sphaerochaeta*, *Spirochaeta*, and *Treponema* and another consisting of the genera *Borrelia* and *Cristispira*.

### Taxonomic implications

The results presented here show that the main lineages of the phylum Spirochaetes are evolutionarily distinct. The families *Spirochaetaceae*, *Brachyspiraceae*, and *Leptospiraceae* are distinguished from each other and all other bacteria by large numbers of identified CSIs in widely distributed proteins. Additionally, these three families branch distinctly in both 16S rRNA based and concatenated protein based phylogenetic trees. The results presented here also show that the family *Spirochaetaceae* consists of two distinct monophyletic groups. The distinctiveness of these groups is supported by both molecular evidence, in the form of the large numbers of discovered CSIs, and phylogenetic analyses. Additionally, both of these distinct groups exhibit a large amount of phylogenetic diversity which is currently not reflected in their taxonomy. The current taxonomic organization of the phylum Spirochaetes places all of the main lineages (viz. *Spirochaetaceae*, *Brachyspiraceae*, *Leptospiraceae*, and *Brevinemataceae*) into a single order. However, to adequately recognize both distinctiveness of the main lineages within the phylum Spirochaetes and the distinctiveness and diversity of the two main groups within the family *Spirochaetaceae*, the main lineages of the phylum Spirochaetes would have to have their taxonomic rank increased. To recognize the distinctiveness of both the main lineages within the phylum Spirochaetes and the two main groups within the family *Spirochaetaceae* we are proposing a taxonomic rearrangement of the phylum as follows: We propose that the family *Leptospiraceae* be transferred to the novel order *Leptospiriales* ord. nov. within the class *Spirochaetia*, the family *Brachyspiraceae* be transferred to the novel order *Brachyspiriales* ord. nov. within the class *Spirochaetia*, the family *Brevinemataceae* be transferred to the novel order *Brevinematales* ord. nov. within the class *Spirochaetia*, and that the genera *Borrelia* and *Cristispira* be transferred to the novel family *Borreliaceae* fam. nov. within the order *Spirochaetales* (Figure 8). The emended descriptions of the order *Spirochaetales* and the family *Spirochaetaceae*, as well as a description of the new taxonomic groups *Leptospiriales* ord. nov., *Brachyspiriales* ord. nov., *Brevinematales* ord. nov., and *Borreliaceae* fam. nov. are provided below.

### Emended description of the order *Spirochaetales* (Buchanan, 1917)

The order contains two families, *Spirochaetaceae* and *Borreliaceae*, of which *Spirochaetaceae* is the type family. Organisms are helical or coccoid, 0.1–75 μm in diameter and 3.5–250 μm in length. Cells do not have hooked ends. Cells may possess flagella. Periplasmic flagella overlap in the central region of the cell. The diamino acid component of the peptidoglycan is L-ornithine. Anaerobic, facultatively anaerobic, or microaerophilic. Organisms are Chemo-organotrophic and utilize carbohydrates or amino acids as carbon and energy sources. Both free living and host associated members. The G + C content of the DNA is 27–66 (mol%). The type genus is *Spirochaeta* (Ehrenberg, 1835).

Organisms from this order are distinguished from all other Bacteria by the conserved signature indels (CSIs) described in this report in the following proteins: Alanyl-tRNA synthetase, Phosphoribosylpyrophosphate synthetase, SecY preprotein translocase, peptide chain release factor 2, DNA mismatch repair protein MutS, and DNA mismatch repair protein MutL.

### Emended description of the family *Spirochaetaceae* (Swellengrebel 1907 emend. Abt et al., 2012)

The family contains seven genera, *Clevelandina, Diplocalyx, Hollandina, Pillotina, Sphaerochaeta*, *Spirochaeta*, and *Treponema* of which *Spirochaeta* is the type genus. Organisms are helical or coccoid, 0.1–75 μm in diameter and 5–250 μm in length. Cells do not have hooked ends. Cells may possess flagella. Periplasmic flagella overlap in the central region of the cell. Cells can be anaerobic or facultatively anaerobic. The diamino acid component of the peptidoglycan is L-ornithine. Organisms are chemo-organotrophic and utilize carbohydrates or amino acids as carbon and energy sources. Both free living and host associated members. The G + C content of the DNA is 36–66 (mol%).

Organisms from this family are distinguished from all other bacteria by the CSIs described in this report in the following proteins: 6-phosphofructokinase (pyrophosphate), bifunctional Hpr kinase/phosphatase, and 30S ribosomal protein S13.

### Description of *Borreliaceae* fam. nov.

*Borreliaceae* (Bor.re'li.a'ce.ae. N.L. fem. n. *Borrelia* type genus of the family; -aceae ending to denote a family; M.L. fem. pl. n. *Borreliaceae* the *Borrelia* family).

The family contains two genera, *Borrelia* and *Cristispira* of which *Borrelia* is the type genus. Organisms are helical, 0.2–3 μm in diameter and 3–180 μm in length. Cells do not have hooked ends. Periplasmic flagella overlap in the central region of the cell. Cells are motile, host-associated, and microaerophilic. The diamino acid component of the peptidoglycan is L-ornithine. Organisms are chemo-organotrophic and utilize carbohydrates or amino acids as carbon and energy sources. The G + C content of the DNA is 27–32 (mol%).

Organisms from this family are distinguished from all other Bacteria by the CSIs described in this report in the following proteins: Phosphofructokinase, 50S ribosomal protein L4, tRNA pseudouridine 55 synthase, Translation elongation factor-Tu, Histidyl-tRNA synthetase, Seryl-tRNA synthetase, Spoiiij-associtated protein, Nicotinate phosphoribosyltransferase, Ribose 5-phosphate isomerase, Ribonuclease Z, Hypothetical protein BGAFAR04_0762, Signal recognition particle subunit FFH/SRP54, Hypothetical protein BSV1_0075, Aspartyl/glutamyl-tRNA amidotransferase subunit A, Ribosomal RNA methyltransferase, and a LysM domain/M23/M37 peptidase domain protein.

### Description of *Brachyspiriales* ord. nov.

*Brachyspiriales* (Bra.chy.spi.ra'les. N.L. fem. n. Brachyspira type genus of the order; suff. -*ales* ending to denote an order; N.L. fem. pl. n. *Brachyspiriales* the order of *Brachyspira*).

The order contains the type family *Brachyspiraceae*. Organisms are helical, 0.2–0.4 μm in diameter and 2–11 μm in length. Cell ends may be blunt or pointed and do not have hooked ends. Periplasmic flagella overlap in the central region of the cell. Cells are motile, host-associated, and obligately anaerobic and aerotolerant. The diamino acid component of the peptidoglycan is L-ornithine. Organisms are Chemo-organotrophic and utilize monosaccharides, disaccharides, the trisaccharide trehalose, and amino sugars as carbon and energy sources. The G + C content of the DNA is 24–28(mol%). The type genus is *Brachyspira* (Hovind-Hougen et al., 1982).

Organisms from this order are distinguished from all other bacteria by the CSIs described in this report in the following proteins: Flagellar hook-associated protein FlgK, DNA polymerase I, Valyl-tRNA synthetase, ATP-dependent protease La, and Glutamyl-tRNA amidotransferase subunit B. The description of the family *Brachyspiraceae* is the same as that of the order *Brachyspiriales*.

### Description of *Brevinematales* ord. nov.

*Brevinematales* (Bre.vi.ne.ma.ta'les. N.L. fem. n. *Brevinema* -*atos* type genus of the order; suff. -*ales* ending to denote an order; N.L. fem. pl. n. *Brevinematales* the order of *Brevinema*).

The description of the order is the same as the description of the type family, *Brevinemataceae*.

### Description of *Leptospiriales* ord. nov.

*Leptospiriales* (Lep.to.spi.ra'les. N.L. fem. n. *Leptospira* type genus of the order; suff. -*ales* ending to denote an order; N.L. fem. pl. n. *Leptospiriales* the order of *Leptospira*).

The order contains the type family *Leptospiraceae*. Organisms are helical, 0.1–0.3 μm in diameter and 2–11 μm in length. Cell have hooked ends. Periplasmic flagella do not overlap in the central region of the cell. Cells are motile. The diamino acid component of the peptidoglycan is α,ε-diaminopimelic acid. Obligately aerobic or microaerophilic. Organisms are Chemo-organotrophic and long-chain fatty acids or long-chain fatty alcohols as carbon and energy sources. Both free living and host associated members. The G + C content of the DNA is 33–55 (mol%). The type genus is *Leptospira* (Noguchi, 1917).

Organisms from this order are distinguished from all other Bacteria by the CSIs described in this report in the following proteins: 50S Ribosomal protein L14, 30S Ribosomal protein S2, Alanyl-tRNA synthetase, Flagellar basal-body rod protein FlgG, and Flagellar filament core protein FlaB. The description of the family *Leptospiraceae* is the same as that of the order *Leptospiriales*.

### Conflict of interest statement

The authors declare that the research was conducted in the absence of any commercial or financial relationships that could be construed as a potential conflict of interest.

## References

[B1] Abt B. Göker M. Scheuner C. Han C. Lu M. Misra M. (2013). Genome sequence of the thermophilic fresh-water bacterium *Spirochaeta caldaria* type strain (H1 ^T^), reclassification of *Spirochaeta caldaria* and *Spirochaeta stenostrepta* in the genus *Treponema* as *Treponema caldaria* comb. nov., *Treponema stenostrepta* comb. nov., and *Treponema zuelzerae* comb. nov., and emendation of the genus *Treponema*. Stand. Genomic Sci. 8 [Epub ahead of print]. 10.4056/sigs.3096473 PMC3739177 23961314

[B2] Abt B. Han C. Scheuner C. Lu M. Lapidus A. Nolan M. (2012). Complete genome sequence of the termite hindgut bacterium *Spirochaeta coccoides* type strain (SPN1^T^), reclassification in the genus *Sphaerochaeta* as *Sphaerochaeta coccoides* comb. nov. and emendations of the family *Spirochaetaceae* and the genus *Sphaerochaeta*. Stand. Genomic Sci. 6, 194 10.4056/sigs.2796069 22768363 PMC3388779

[B3] Adeolu M. Gupta R. S. (2013). Phylogenomics and molecular signatures for the order *Neisseriales*: proposal for division of the order *Neisseriales* into the emended family *Neisseriaceae* and *Chromobacteriaceae* fam. nov. Antonie Van Leeuwenhoek 104, 1–24 10.1007/s10482-013-9920-6 23575986

[B4] Adler B. de la Peña Moctezuma A. (2010). *Leptospira* and leptospirosis. Vet. Microbiol. 140, 287–296 10.1016/j.vetmic.2009.03.012 19345023

[B5] Akiva E. Itzhaki Z. Margalit H. (2008). Built-in loops allow versatility in domain? Domain interactions: lessons from self-interacting domains. Proc. Natl. Acad. Sci. U.S.A. 105, 13292 10.1073/pnas.0801207105 18757736 PMC2533183

[B6] Altschul S. F. Madden T. L. Schäffer A. A. Zhang J. Zhang Z. Miller W. (1997). Gapped, BLAST and PSI-BLAST: a new generation of protein database search programs. Nucleic Acids Res. 25, 3389–3402 10.1093/nar/25.17.3389 9254694 PMC146917

[B7] Anthony N. E. Blackwell J. Ahrens W. Lovell R. Scobey M. W. (2013). Intestinal spirochetosis: an Enigmatic disease. Dig. Dis. Sci. 58, 202–208 10.1007/s10620-012-2305-2 22851039

[B8] Baldauf S. L. Palmer J. D. (1993). Animals and fungi are each other's closest relatives: congruent evidence from multiple proteins. Proc. Natl. Acad. Sci. U.S.A. 90, 11558–11562 10.1073/pnas.90.24.11558 8265589 PMC48023

[B9] Bellgard M. I. Wanchanthuek P. La T. Ryan K. Moolhuijzen P. Albertyn Z. (2009). Genome sequence of the pathogenic intestinal spirochete *Brachyspira hyodysenteriae* reveals adaptations to its lifestyle in the porcine large intestine. PloS ONE 4:e4641 10.1371/journal.pone.0004641 19262690 PMC2650404

[B10] Bermudes D. Chase D. Margulis L. (1988). Morphology as a basis for taxonomy of large Spirochetes symbiotic in wood-eating cockroaches and termites: *Pillotina* gen. nov., nom. rev.; *Pillotina calotermitidis* sp. nov., nom. rev.; *Diplocalyx* gen. nov., nom. rev.; *Diplocalyx calotermitidis* sp. nov., nom. rev.; *Hollandina* gen. nov., nom. rev.; *Hollandina pterotermitidis* sp. nov., nom. rev.; and *Clevelandina reticulitermitidis* gen. nov., sp. nov. Int. J. Syst. Bacteriol. 38, 291–302 10.1099/00207713-38-3-291 11542253

[B11] Bhandari V. Ahmod N. Z. Shah H. N. Gupta R. S. (2013). Molecular signatures for the *Bacillus* species: demarcation of the *Bacillus subtilis* and *Bacillus cereus* clades in molecular terms and proposal to limit the placement of new species into the genus *Bacillus*. Int. J. Syst. Evol. Microbiol. 63, 2712–2726 10.1099/ijs.0.048488-0 23475340

[B78] Buchanan R. E. (1917). Studies in the nomenclature and classification of bacteria. II. The primary subdivisions of the Schizomycetes. J. Bacteriol. 2, 155–164 16558735 10.1128/jb.2.2.155-164.1917 PMC378699

[B12] Bulach D. M. Zuerner R. L. Wilson P. Seemann T. McGrath A. Cullen P. A. (2006). Genome reduction in *Leptospira borgpetersenii* reflects limited transmission potential. Proc. Natl. Acad. Sci. U.S.A. 103, 14560–14565 10.1073/pnas.0603979103 16973745 PMC1599999

[B13] Casjens S. R. Fraser-Liggett C. M. Mongodin E. F. Qiu W. G. Dunn J. J. Luft B. J. (2011). Whole genome sequence of an unusual *Borrelia burgdorferi* sensu lato isolate. J. Bacteriol. 193, 1489–1490 10.1128/JB.01521-10 21217002 PMC3067611

[B14] Castresana J. (2000). Selection of conserved blocks from multiple alignments for their use in phylogenetic analysis. Mol. Biol. Evol. 17, 540–552 10.1093/oxfordjournals.molbev.a026334 10742046

[B15] Cavalier-Smith T. (2002). The neomuran origin of archaebacteria, the negibacterial root of the universal tree and bacterial megaclassification. Int. J. Syst. Evol. Microbiol. 52, 7–76 11837318 10.1099/00207713-52-1-7

[B16] Chaconas G. (2005). Hairpin telomeres and genome plasticity in *Borrelia*: all mixed up in the end. Mol. Microbiol. 58, 625–635 10.1111/j.1365-2958.2005.04872.x 16238614

[B17] Chaconas G. Kobryn K. (2010). Structure, function, and evolution of linear replicons in *Borrelia*. Annu. Rev. Microbiol. 64, 185–202 10.1146/annurev.micro.112408.134037 20536352

[B18] Chou L. F. Chen Y. T. Lu C. W. Ko Y. C. Tang C. Y. Pan M. J. (2012). Sequence of *Leptospira santarosai* serovar Shermani genome and prediction of virulence-associated genes. Gene. 511, 364–370 10.1016/j.gene.2012.09.074 23041083

[B19] Ciccarelli F. D. Doerks T. Von Mering C. Creevey C. J. Snel B. Bork P. (2006). Toward automatic reconstruction of a highly resolved tree of life. Science 311, 1283–1287 10.1126/science.1123061 16513982

[B20] Cutler S. J. (2010). Relapsing fever: a forgotten disease revealed. J. Appl. Microbiol. 108, 1115–1122 10.1111/j.1365-2672.2009.04598.x 19886891

[B21] Dai Q. Restrepo B. I. Porcella S. F. Raffel S. J. Schwan T. G. Barbour A. G. (2006). Antigenic variation by *Borrelia hermsii* occurs through recombination between extragenic repetitive elements on linear plasmids. Mol. Microbiol. 60, 1329–1343 10.1111/j.1365-2958.2006.05177.x 16796672 PMC5614446

[B22] Dworkin M. S. Schwan T. G. Anderson D. E. Jr. Borchardt S. M. (2008). Tick-borne relapsing fever. Infect. Dis. Clin. North Am. 22, 449 10.1016/j.idc.2008.03.006 18755384 PMC3725823

[B79] Ehrenberg C. G. (1835). Dritter Beitrag zur Erkenntniss grosser Organisation in der Richtung des kleinsten Raumes, in Abhandlungen der Preussischen Akademie der Wissenschaften (Berlin) aus den Jahre 1833–1835, 143–336

[B23] Elbir H. Gimenez, G, Robert C. Bergström S. Cutler S. Raoult D. (2012). Complete genome sequence of *Borrelia crocidurae*. J. Bacteriol. 194, 3723–3724 10.1128/JB.00118-12 22740657 PMC3393517

[B24] Ellen R. P. Galimanas V. B. (2005). Spirochetes at the forefront of periodontal infections. periodontology 2000 38, 13–32 10.1111/j.1600-0757.2005.00108.x 15853935

[B25] Euzéby J. P. (2013). List of Prokaryotic names with Standing in Nomenclature. Available online at: www.bacterio.net

[B26] Fraser C. M. Casjens S. Huang W. M. Sutton G. G. Clayton R. Lathigra R. (1997). Genomic sequence of a Lyme disease spirochaete, *Borrelia burgdorferi*. Nature 390, 580–586 10.1038/37551 9403685

[B27] Gao B. Gupta R. S. (2012a). Microbial systematics in the post-genomics era. Antonie Van Leeuwenhoek 101, 45–54 10.1007/s10482-011-9663-1 22048742

[B28] Gao B. Gupta R. S. (2012b). Phylogenetic framework and molecular signatures for the main clades of the phylum Actinobacteria. Microbiol. Mol. Biol. Rev. 76, 66–112 10.1128/MMBR.05011-11 22390973 PMC3294427

[B29] Gerbase A. C. Rowley J. T. Heymann D. H. L. Berkley S. F. B. Piot P. (1998). Global prevalence and incidence estimates of selected curable STDs. Sex. Transm. Infect. 74, S12 10023347

[B30] Glöckner G. Lehmann R. Romualdi A. Pradella S. Schulte-Spechtel U. Schilhabel M. (2004). Comparative analysis of the *Borrelia garinii* genome. Nucleic Acids Res. 32, 6038–6046 10.1093/nar/gkh953 15547252 PMC534632

[B31] Gogarten J. P. Doolittle W. F. Lawrence J. G. (2002). Prokaryotic evolution in light of gene transfer. Mol. Biol. Evol. 19, 2226–2238 10.1093/oxfordjournals.molbev.a004046 12446813

[B32] Goodner B. Hinkle G. Gattung S. Miller N. Blanchard M. Qurollo B. (2001). Genome sequence of the plant pathogen and biotechnology agent *Agrobacterium tumefaciens* C58. Science 294, 2323–2328 10.1126/science.1066803 11743194

[B33] Griffiths E. Gupta R. S. (2004). Signature sequences in diverse proteins provide evidence for the late divergence of the order *Aquificales*. Int. Microbiol. 7, 41–52 15179606

[B34] Gupta R. S. (1998). Protein phylogenies and signature sequences: a reappraisal of evolutionary relationships among archaebacteria, eubacteria, and eukaryotes. Microbiol. Mol. Biol. Rev. 62, 1435 9841678 10.1128/mmbr.62.4.1435-1491.1998 PMC98952

[B35] Gupta R. S. (2009). Protein signatures (molecular synapomorphies) that are distinctive characteristics of the major cyanobacterial clades. Int. J. Syst. Evol. Microbiol. 59, 2510 19622649 10.1099/ijs.0.005678-0

[B36] Gupta R. S. (2010). Molecular signatures for the main phyla of photosynthetic bacteria and their subgroups. Photosyn. Res. 104, 357–372 10.1007/s11120-010-9553-9 20414806

[B37] Gupta R. S. Chander P. George S. (2012). Phylogenetic framework and molecular signatures for the class *Chloroflexi* and its different clades; proposal for division of the class *Chloroflexi* class. nov. into the suborder *Chloroflexineae* subord. nov., consisting of the emended family *Oscillochloridaceae* and the family *Chloroflexaceae* fam. nov., and the suborder *Roseiflexineae* subord. nov., containing the family *Roseiflexaceae* fam. nov. Antonie Van Leeuwenhoek 103, 99–119 10.1007/s10482-012-9790-3 22903492

[B38] Gupta R. S. Chen W. J. Adeolu M. Chai Y. (2013). Molecular signatures for the class *Coriobacteriia* and its different clades; Proposal for division of the class *Coriobacteriia* into the emended order *Coriobacteriales*, containing the emended family *Coriobacteriaceae* and *Atopobiaceae* fam. nov., and *Eggerthellales* ord. nov., containing the family *Eggerthellaceae* fam. nov. Int. J. Syst. Evol. Microbiol. [Epub ahead of print]. 10.1099/ijs.0.048371-0 23524353

[B39] Gupta R. S. Griffiths E. (2002). Critical issues in bacterial phylogeny. Theor. Popul. Biol. 61, 423–434 10.1006/tpbi.2002.1589 12167362

[B40] Håfström T. Jansson D. S. Segerman B. (2011). Complete genome sequence of *Brachyspira intermedia* reveals unique genomic features in *Brachyspira* species and phage-mediated horizontal gene transfer. BMC Genomics 12:395 10.1186/1471-2164-12-395 21816042 PMC3163572

[B41] Han C. Gronow S. Teshima H. Lapidus A. Nolan M. Lucas S. (2011). Complete genome sequence of *Treponema succinifaciens* type strain (6091^T^). Stand. Genomic Sci. 4, 361 10.4056/sigs.1984594 21886863 PMC3156407

[B42] Harris J. K. Kelley S. T. Spiegelman G. B. Pace N. R. (2003). The genetic core of the universal ancestor. Genome Res. 13, 407–412 10.1101/gr.652803 12618371 PMC430263

[B43] Harwood C. S. Canale-Parola E. (1984). Ecology of spirochetes. Annu. Rev. Microbiol. 38, 161–192 10.1146/annurev.mi.38.100184.001113 6388490

[B44] Homma M. DeRosier D. J. Macnab R. M. (1990). Flagellar hook and hook-associated proteins of *Salmonella typhimurium* and their relationship to other axial components of the flagellum. J. Mol. Biol. 213, 819–832 10.1016/S0022-2836(05)80266-9 2193164

[B80] Hovind-Hougen K. Birch-Andersen A. Hendrik-Nielsen R. Orholm M. Pedersen J. O. Teglbaerg P. S. (1982). Intestinal spirochetosis: morphological characterization and cultivation of the spirochete Brachyspira aalborgi gen. nov., sp. nov. J. Clin. Microbiol. 6, 1127–1136 6186689 10.1128/jcm.16.6.1127-1136.1982 PMC272552

[B45] Jeanmougin F. Thompson J. D. Gouy M. Higgins D. G. Gibson T. J. (1998). Multiple sequence alignment with Clustal, X. Trends Biochem. Sci. 23, 403 10.1016/S0968-0004(98)01285-7 9810230

[B46] Jones D. T. Taylor W. R. Thornton J. M. (1992). The rapid generation of mutation data matrices from protein sequences. Comput. Appl. Biosci. 8, 275–282 1633570 10.1093/bioinformatics/8.3.275

[B47] Kobryn K. (2007). The linear hairpin replicons of Borrelia burgdorferi, in Microbial Linear Plasmids, eds Meinhardt F. Klassen R. (Heidelberg: Springer), 117–140

[B48] Lescot M. Audic S. Robert C. Nguyen T. T. Blanc G. Cutler S. J. (2008). The genome of *Borrelia recurrentis*, the agent of deadly louse-borne relapsing fever, is a degraded subset of tick-borne *Borrelia duttonii*. PLoS Genet. 4:e1000185 10.1371/journal.pgen.1000185 18787695 PMC2525819

[B49] Li C. Wolgemuth C. W. Marko M. Morgan D. G. Charon N. W. (2008). Genetic analysis of spirochete flagellin proteins and their involvement in motility, filament assembly, and flagellar morphology. J. Bacteriol. 190, 5607–5615 10.1128/JB.00319-08 18556797 PMC2519375

[B50] Lin C. den Bakker H. C. Suzuki H. Lefébure T. Ponnala L. Sun Q. (2013). Complete genome sequence of the porcine strain *Brachyspira pilosicoli* P43/6/78^T^. Genome Announc. 1 [Epub ahead of print]. 10.1128/genomeA.00215-12 23469345 PMC3587939

[B51] Ludwig W. Klenk H. P. (2001). Overview: a phylogenetic backbone and taxonomic framework for prokaryotic systematics, in Bergey's Manual of Systematic Bacteriology, ed Garrity G. M. (New York, NY: Springer), 49–65

[B52] Macnab R. M. (2003). How bacteria assemble flagella. Annu. Rev. Microbiol. 57, 77–100 10.1146/annurev.micro.57.030502.090832 12730325

[B53] Mavromatis K. Yasawong M. Chertkov O. Lapidus A. Lucas S. Nolan M. (2010). Complete genome sequence of *Spirochaeta smaragdinae* type strain (SEBR 4228^T^). Stand. Genomic Sci. 3, 136 10.4056/sigs.1143106 21304743 PMC3035371

[B54] Nau R. Christen H. J. Eiffert H. (2009). Lyme disease: ?current state of knowledge. Dtsch. Arztebl. Int. 106, 72 10.3238/arztebl.2009.0072 19562015 PMC2695290

[B55] NCBI. (2013). NCBI Genome Database. Available online at: http://www.ncbi.nlm.nih.gov/genome/

[B81] Noguchi H. (1917). Spirochaeta icterohaemorrhagiae in American wild rats and its relation to the Japanese and European strains. J. Exp. Med. 25, 755–763 19868121 10.1084/jem.25.5.755 PMC2125512

[B56] Olsen I. Paster B. J. Dewhirst F. E. (2000). Taxonomy of spirochetes. Anaerobe 6, 39–57 10.1006/anae.1999.0319

[B57] Paster B. J. (2011a). Phylum XV. Spirochaetes Garrity and Holt (2001), in Bergey's Manual of Systematic Bacteriology, eds Brenner D. J. Krieg N. R. Garrity G. M. Staley J. T. (New York, NY: Springer), 471

[B58] Paster B. J. (2011b). Family I. Spirochaetaceae Swellengrebel 1907, 581AL, in Bergey's Manual of Systematic Bacteriology, eds Brenner D. J. Krieg N. R. Garrity G. M. Staley J. T. (New York, NY: Springer), 473–531

[B59] Paster B. J. Dewhirst F. E. (2000). Phylogenetic foundation of spirochetes. J. Mol. Microbiol. Biotechnol. 2, 341–344 11075904

[B60] Paster B. J. Dewhirst F. E. Weisburg W. G. Tordoff L. A. Fraser G. J. Hespell R. B. (1991). Phylogenetic analysis of the spirochetes. J. Bacteriol. 173, 6101–6109 1917844 10.1128/jb.173.19.6101-6109.1991 PMC208357

[B61] Pati A. Sikorski J. Gronow S. Munk C. Lapidus A. Copeland A. (2010). Complete genome sequence of *Brachyspira murdochii* type strain (56–150^T^). Stand. Genomic Sci. 2, 260 21304710 10.4056/sigs.831993 PMC3035287

[B62] Picardeau M. Bulach D. M. Bouchier C. Zuerner R. L. Zidane N. Wilson P. J. (2008). Genome sequence of the saprophyte *Leptospira biflexa* provides insights into the evolution of *Leptospira* and the pathogenesis of leptospirosis. PloS ONE 3:e1607 10.1371/journal.pone.0001607 18270594 PMC2229662

[B63] Quast C. Pruesse E. Yilmaz P. Gerken J. Schweer T. Yarza P. (2013). The, SILVA ribosomal, RNA gene database project: improved data processing and web-based tools. Nucleic Acids Res. 41, D590–D596 10.1093/nar/gks1219 23193283 PMC3531112

[B64] Ren S. X. Fu G. Jiang X. G. Zeng R. Miao Y. G. Xu H. (2003). Unique physiological and pathogenic features of *Leptospira interrogans* revealed by whole-genome sequencing. Nature 422, 888–893 10.1038/nature01597 12712204

[B65] Rokas A. Holland P. W. H. (2000). Rare genomic changes as a tool for phylogenetics. Trends Ecol. Evol. 15, 454–459 10.1016/S0169-5347(00)01967-4 11050348

[B66] Rokas A. Williams B. L. King N. Carroll S. B. (2003). Genome-scale approaches to resolving incongruence in molecular phylogenies. Nature 425, 798–804 10.1038/nature02053 14574403

[B67] Schutzer S. E. Fraser-Liggett C. M. Qiu W. G. Kraiczy P. Mongodin E. F. Dunn J. J. (2012). Whole-genome sequences of *Borrelia bissettii*, *Borrelia valaisiana*, and *Borrelia spielmanii*. J. Bacteriol. 194, 545–546 10.1128/JB.06263-11 22207749 PMC3256645

[B68] Seshadri R. Myers G. S. A. Tettelin H. Eisen J. A. Heidelberg J. F. Dodson R. J. (2004). Comparison of the genome of the oral pathogen *Treponema denticola* with other spirochete genomes. Proc. Natl. Acad. Sci. U.S.A. 101, 5646–5651 10.1073/pnas.0307639101 15064399 PMC397461

[B69] Singh B. Gupta R. S. (2009). Conserved inserts in the Hsp60 (GroEL) and Hsp70 (DnaK) proteins are essential for cellular growth. Mol. Genet. Genomics 281, 361–373 10.1007/s00438-008-0417-3 19127371

[B70] Smajs D. Norris S. J. Weinstock G. M. (2012). Genetic diversity in Treponema pallidum: implications for pathogenesis, evolution and molecular diagnostics of syphilis and yaws. Infect. Genet. Evol. 12, 191–202 10.1016/j.meegid.2011.12.001 22198325 PMC3786143

[B71] Smajs D. Zobaníková M. Strouhal M. Cjková D. Dugan-Rocha S. Pospísilová P. (2011). Complete genome sequence of *Treponema paraluiscuniculi*, strain Cuniculi A: the loss of infectivity to humans is associated with genome decay. PLoS ONE 6:e20415 10.1371/journal.pone.0020415 21655244 PMC3105029

[B72] Tamura K. Peterson D. Peterson N. Stecher G. Nei M. Kumar S. (2011). MEGA5: molecular evolutionary genetics analysis using maximum likelihood, evolutionary distance, and maximum parsimony methods. Mol. Biol. Evol. 28, 2731–2739 10.1093/molbev/msr121 21546353 PMC3203626

[B73] Tavaré S. (1986). Some probabilistic and statistical problems in the analysis of, DNA sequences, in Lectures on Mathematics in the Life Sciences, ed Miura R. M. (Providence, RI: American Mathematical Society), 57–86

[B74] Visser M. B. Ellen R. P. (2011). New insights into the emerging role of oral spirochaetes in periodontal disease. Clin. Microbiol. Infect. 17, 502–512 10.1111/j.1469-0691.2011.03460.x 21414084

[B75] Whelan S. Goldman N. (2001). A general empirical model of protein evolution derived from multiple protein families using a maximum-likelihood approach. Mol. Biol. Evol. 18, 691–699 10.1093/oxfordjournals.molbev.a003851 11319253

[B76] Wu D. Hugenholtz P. Mavromatis K. Pukall R. Dalin E. Ivanova N. N. (2009). A phylogeny-driven genomic encyclopaedia of Bacteria and Archaea. Nature 462, 1056–1060 10.1038/nature08656 20033048 PMC3073058

[B77] Zhong J. Barbour A. G. (2004). Cross species hybridization of a *Borrelia burgdorferi* DNA array reveals infection and culture associated genes of the unsequenced genome of the relapsing fever agent *Borrelia hermsii*. Mol. Microbiol. 51, 729–748 10.1046/j.1365-2958.2003.03849.x 14731275

