# Erratum: A phylogenomic and molecular signature based approach for characterization of the Phylum Spirochaetes and its major clades: proposal for a taxonomic revision of the phylum

**DOI:** 10.3389/fmicb.2013.00322

**Published:** 2013-10-31

**Authors:** Radhey S. Gupta, Sharmeen Mahmood, Mobolaji Adeolu

**Affiliations:** Department of Biochemistry and Biomedical Sciences, McMaster UniversityHamilton, ON, Canada

**Keywords:** spirochaetes phylogeny and taxonomy, molecular signatures, spirochaetales, spirochaetaceae, borreliaceae, brachyspirales, leptospirales, conserved signature indels

Dr. Aharon Oren, the Editor and one of the List Editors of the Journal IJSEM, has informed us of a few minor errors in the protologues present in this publication which would prevent valid publication of the proposed names. The nomenclatural types we proposed for the new orders *Brachyspirales* ord. nov., *Brevinematales* ord. nov. and *Leptospirales* ord. nov. and the emended description we proposed for the order *Spirochaetales* (Buchanan, 1917) (Approved Lists 1980) were not in accordance with Rule 21a of the Bacteriological code and the etymology of the names of the family *Borreliaceae* fam. nov. and the orders *Brachyspirales* ord. nov., *Brevinematales* ord. nov. and *Leptospirales* ord. nov. were not in accordance with Rule 9 and Recommendation 6 of the Bacteriological code (Lapage et al., 1992). Hence, we are resubmitting revised protologues of the emendations and newly proposed taxa, correcting the minor errors present in the original submission.

## Emended description of the order *Spirochaetales* Buchanan, 1917 (approved lists 1980)

The order contains two families, *Spirochaetaceae* and *Borreliaceae*. Organisms are helical or coccoid, 0.1–75 μm in diameter and 3.5–250 μm in length. Cells do not have hooked ends. Cells may possess flagella. Periplasmic flagella overlap in the central region of the cell. The diamino acid component of the peptidoglycan is L-ornithine. Anaerobic, facultatively anaerobic, or microaerophilic. Organisms are chemo-organotrophic and utilize carbohydrates or amino acids as carbon and energy sources. Both free living and host associated members. The G+C content of the DNA is 27–66 (mol%). The type genus is *Spirochaeta* Ehrenberg 1835 (Approved Lists 1980) Skerman et al. (1980).

Organisms from this order are distinguished from all other bacteria examined to date by the conserved signature indels described in this report in the following proteins: alanyl-tRNA synthetase, phosphoribosylpyrophosphate synthetase, SecY preprotein translocase, peptide chain release factor 2, DNA mismatch repair protein MutS, and DNA mismatch repair protein MutL.

## Emended description of the family *Spirochaetaceae* Swellengrebel, 1907 (approved lists 1980) emend. Abt et al. (2013)

The family contains seven genera, *Clevelandina, Diplocalyx, Hollandina, Pillotina, Sphaerochaeta*, *Spirochaeta*, and *Treponema*. Organisms are helical or coccoid, 0.1–75 μm in diameter and 5–250 μm in length. Cells do not have hooked ends. Cells may possess flagella. Periplasmic flagella overlap in the central region of the cell. Cells can be anaerobic or facultatively anaerobic. The diamino acid component of the peptidoglycan is L-ornithine. Organisms are chemo-organotrophic and utilize carbohydrates or amino acids as carbon and energy sources. Both free living and host associated members. The G+C content of the DNA is 36–66 (mol%). The type genus is *Spirochaeta* Ehrenberg 1835 (Approved Lists 1980) Skerman et al. (1980).

Organisms from this family are distinguished from all other bacteria examined to date by the conserved signature indels described in this report in the following proteins: 6-phosphofructokinase (pyrophosphate), bifunctional Hpr kinase/phosphatase and 30S ribosomal protein S13.

## Description of *Borreliaceae* fam. nov.

*Borreliaceae* (Bor.re'li.a'ce.ae. N.L. fem. n. *Borrelia* type genus of the family; -aceae ending to denote a family; N.L. fem. pl. n. *Borreliaceae* the family whose nomenclatural type is the genus *Borrelia*).

The family contains two genera *Borrelia* and *Cristispira*. Organisms are helical, 0.2–3 μm in diameter and 3–180 μm in length. Cells do not have hooked ends. Periplasmic flagella overlap in the central region of the cell. Cells are motile, host-associated and microaerophilic. The diamino acid component of the peptidoglycan is L-ornithine. Organisms are chemo-organotrophic and utilize carbohydrates or amino acids as carbon and energy sources. The G+C content of the DNA is 27–32 (mol%). The type genus is *Borrelia* (Swellengrebel, 1907) (Approved Lists 1980).

Organisms from this family are distinguished from all other bacteria examined to date by the conserved signature indels described in this report in the following proteins: phosphofructokinase, 50S ribosomal protein L4, tRNA pseudouridine 55 synthase, translation elongation factor-Tu, histidyl-tRNA synthetase, seryl-tRNA synthetase, spoiiij-associtated protein, nicotinate phosphoribosyltransferase, ribose 5-phosphate isomerase, ribonuclease Z, hypothetical protein BGAFAR04_0762, signal recognition particle subunit FFH/SRP54, hypothetical protein BSV1_0075, aspartyl/glutamyl-tRNA amidotransferase subunit A, ribosomal RNA methyltransferase and a LysM domain/M23/M37 peptidase domain protein.

## Description of *Brachyspirales* ord. nov.

*Brachyspirales* (Bra.chy.spi.ra'les. N.L. fem. n. *Brachyspira* type genus of the order; suff. -*ales* ending to denote an order; N.L. fem. pl. n. *Brachyspirales* the order whose nomenclatural type is the genus *Brachyspira*).

The order contains one family, *Brachyspiraceae*. Organisms are helical, 0.2–0.4 μm in diameter and 2–11 μm in length. Cell ends may be blunt or pointed and do not have hooked ends. Periplasmic flagella overlap in the central region of the cell. Cells are motile, host-associated and obligately anaerobic and aerotolerant. The diamino acid component of the peptidoglycan is L-ornithine. Organisms are chemoorganotrophic and utilize monosaccharides, disaccharides, the trisaccharide trehalose, and amino sugars as carbon and energy sources. The G+C content of the DNA is 24–28 (mol%). The type genus is *Brachyspira* (Hovind-Hougen, 1983).

Organisms from this order are distinguished from all other bacteria examined to date by the conserved signature indels described in this report in the following proteins: flagellar hook-associated protein FlgK, DNA polymerase I, valyl-tRNA synthetase, ATP-dependent protease La and glutamyl-tRNA amidotransferase subunit B.

## Emended description of the family *Brachyspiraceae* Paster, 2012a

The family contains one genus, *Brachyspira*. The description of the family *Brachyspiraceae* (Paster, 2012a) is the same as that of the order *Brachyspirales* ord. nov. The type genus is *Brachyspira* (Hovind-Hougen, 1983).

## Description of *Brevinematales* ord. nov.

*Brevinematales* (Bre.vi.ne.ma.ta'les. N.L. neut. n. *Brevinema* type genus of the order; suff. -*ales* ending to denote an order; N.L. fem. pl. n. *Brevinematales* the order whose nomenclatural type is the genus *Brevinema*).

The order contains one family, *Brevinemataceae*. The description of this order is the same as that of the family *Brevinemataceae* (Paster, 2012b). The type genus is *Brevinema* (Defosse et al., 1995).

## Description of *Leptospirales* ord. nov.

*Leptospirales* (Lep.to.spi.ra'les. N.L. fem. n. *Leptospira* type genus of the order; suff. -*ales* ending to denote an order; N.L. fem. pl. n. *Leptospirales* the order whose nomenclatural type is the genus *Leptospira*).

The order contains one family, *Leptospiraceae*. Organisms are helical, 0.1–0.3 μm in diameter and 2–11 μm in length. Cells have hooked ends. Periplasmic flagella do not overlap in the central region of the cell. Cells are motile. The diamino acid component of the peptidoglycan is α,ε-diaminopimelic acid. Obligately aerobic or microaerophilic. Organisms are chemoorganotrophic and utilize long-chain fatty acids or long-chain fatty alcohols as carbon and energy sources. Both free living and host-associated members. The G+C content of the DNA is 33–55 (mol%). The type genus is *Leptospira* (Noguchi, 1917) (Approved Lists 1980).

Organisms from this order are distinguished from all other bacteria examined to date by the conserved signature indels described in this report in the following proteins: 50S Ribosomal protein L14, 30S Ribosomal protein S2, alanyl-tRNA synthetase, flagellar basal-body rod protein FlgG, and flagellar filament core protein FlaB.

## Emended description of the family *Leptospiraceae* Hovind-Hougen, 1979 (approved lists 1980) emend. Levett et al. (2005)

The family contains three genera, *Leptonema*, *Leptospira*, and *Turneriella*. The description of the family *Leptospiraceae* (Hovind-Hougen, 1979) (Approved Lists 1980) emend. (Levett et al., 2005) is the same as that of the order *Leptospirales* ord. nov. The type genus is *Leptospira* (Noguchi, 1917) (Approved Lists 1980).
