# Supplementary material for: A phylogenomic and molecular signature based approach for characterization of the phylum Spirochaetes and its major clades: proposal for a taxonomic revision of the phylum
Source: Front Microbiol. 2013 Jul 30;4:217. doi: 10.3389/fmicb.2013.00217 (PMC3726837; doi:10.3389/fmicb.2013.00217)
Supplement: Supplementary file 1 [file 59710_Gupta_DataSheet1.PDF]

Supplemental Table 1

16S rRNA sequences used for phylogenetic analysis

Strain Name	Accession #	Gene Length (bp)
Borrelia afzelii DSM 10508^T	FR733687	1524
Borrelia afzelii PKo	CP000395	1537
Borrelia americana SCW-41^T	EU081285	1362
Borrelia anserine ES-1	U42284	1523
Borrelia burgdorferi B31^T	AE000783	1538
Borrelia carolinensis SCW-22^T	EU085407	1362
Borrelia coriaceae Co53^T	U42286	1519
Borrelia crocidurae Achema	CP003426	1525
Borrelia duttonii Ly	CP000976	1539
Borrelia garinii 20047^T	D67018	1367
Borrelia garinii PBi	CP000013	1538
Borrelia hermsii DAH	AY597657	1273
Borrelia hispanica UESV/246	U42294	1523
Borrelia japonica HO14^T	L40597	1493
Borrelia latyschewii S	JF681793	1423
Borrelia lusitaniae PotiB2^T	X98228	1523
Borrelia miyamotoi HT31^T	D45192	1368
Borrelia parkeri 6232	AF307100	1523
Borrelia persica H1039	HM161645	1526
Borrelia recurrentis A1	CP000993	1539
Borrelia sinica CMN3^T	AB022101	1502
Borrelia sp. SV1	ABJZ02000005	1538
Borrelia spielmanii A14S	ABKB02000009	1537
Borrelia spielmanii DSM 16813^T	HE582779	1523
Borrelia tanukii Hk501^T	D67023	1367
Borrelia turcica IST7^T	AB473539	1539
Borrelia turdi Ya501^T	D67022	1366
Borrelia turicatae 91E135	CP000049	1230
Borrelia valaisiana VS116^T	ABCY02000001	1514
Brachyspira aalborgi NCTC 11492^T	Z22781	1450
Brachyspira alvinipulli C1^T	EF455559	1433
Brachyspira hyodysenteriae ATCC 27164^T	U14930	1433
Brachyspira innocens B256^T	U14920	1433
Brachyspira intermedia PWS/A^T	CP002874	1495
Brachyspira murdochii DSM 12563^T	CP001959	1503
Brachyspira pilosicoli P43/6/78^T	AY155458	1462
Brevinema andersonii ATCC 43811^T	GU993264	1490
Cristispira sp. CP1	U42638	1491

Exilispira thermophila RASEN^T	AB364473	1456
Leptonema illini DSM 21528^T	AHKT01000009	1486
Leptospira alexanderi L60^T	AHMT01000154	1490
Leptospira alstoni 79601^T	AY631881	1431
Leptospira biflexa Patoc 1 (Ames)^T	CP000777	1500
Leptospira borgpetersenii L550	CP000348	1511
Leptospira borgpetersenii Veldrat Bataviae 46^T	AY887899	1431
Leptospira broomii 5399^T	AHMO01000010	1489
Leptospira fainei BUT 6^T	AKWZ01000004	1489
Leptospira inadai 10^T	AHMM01000015	1489
Leptospira interrogans RGA^T	Z12817	1396
Leptospira kirschneri 3522 C^T	AHMN01000021	1489
Leptospira kmetyi Bejo-Iso9^T	AHMP01000041	1490
Leptospira licerasiae VAR 010^T	AHOO02000011	1509
Leptospira meyeri ATCC 43287^T	AY631878	1422
Leptospira noguchii CZ214^T	AKWY01000041	1490
Leptospira santarosai LT 821^T	ADOR01000100	1346
Leptospira terpstrae ATCC 700639^T	AY631888	1422
Leptospira vanthielii ATCC 700522^T	AY631897	1422
Leptospira weilii 2006001853	AFLV02000062	1509
Leptospira weilii Celledoni^T	AY631877	1431
Leptospira wolbachii CDC^T	AY631879	1422
Leptospira wolffii Khorat-H2^T	AKWX01000041	1489
Leptospira yanagawae ATCC 700523^T	AY631882	1422
Sphaerochaeta coccoides DSM 17374^T	CP002659	1523
Sphaerochaeta globosa Buddy^T	CP002541	1535
Sphaerochaeta pleomorpha Grapes^T	AF357917	1530
Spirochaeta africana DSM 8902^T	X93928	1474
Spirochaeta alkalica DSM 8900^T	X93927	1475
Spirochaeta Americana ASpG1^T	AF373921	1467
Spirochaeta asiatica Z-7591^T	X93926	1469
Spirochaeta aurantia J1^T	FR749896	1528
Spirochaeta bajacaliforniensis DSM 16054^T	AJ698859	1521
Spirochaeta cellobiosiphila SIP1^T	EU448140	1435
Spirochaeta dissipatitropha ASpC2^T	AY995150	1459
Spirochaeta halophile RS1^T	M88722	1565
Spirochaeta isovalerica DSM 2461^T	FR749931	1534
Spirochaeta litoralis DSM 2029^T	FR733665	1533
Spirochaeta perfilievii P^T	AY337318	1428
Spirochaeta smaragdinae DSM 11293^T	CP002116	1530
Spirochaeta thermophila DSM 6578^T	CP002903	1534

Treponema amylovorum HA2P^{T}	Y09959	1443
Treponema azotonutricium ZAS-9^{T}	CP001841	1463
Treponema berlinense 7CPL208^{T}	AY230217	1440
Treponema brennaborense DSM 12168^{T}	CP002696	1531
Treponema bryantii RUS-1^{T}	FR749895	1535
Treponema caldaria DSM 7334^{T}	CP002868	1531
Treponema denticola ATCC 35405^{T}	AE017226	1516
Treponema isoptericolens SPIT5^{T}	AM182455	1464
Treponema lecithinolyticum PFB4G^{T}	X87139	1479
Treponema maltophilum BR^{T}	X87140	1494
Treponema medium G7201^{T}	D85437	1381
Treponema pallidum Nichols	AE000520	1495
Treponema paraluiscuniculi Cuniculi A	CP002103	1537
Treponema parvum OMZ 833^{T}	AF302937	1485
Treponema pectinovorum ATCC 33768^{T}	GU562449	1438
Treponema pedis T3552B^{T}	EF061268	1417
Treponema phagedenis F0421	AEFH01000172	1537
Treponema porcinum 14V28^{T}	AY518274	1426
Treponema primitia ZAS-2^{T}	CP001843	1463
Treponema putidum JZC3^{T}	AJ543428	1408
Treponema saccharophilum DSM 2985^{T}	AGRW01000033	1531
Treponema socranskii ATCC 35536^{T}	AF033306	1543
Treponema sp. JC4	JQ783348	1479
Treponema stenostrepta DSM 2028^{T}	FR733664	1528
Treponema succinifaciens DSM 2489^{T}	CP002631	1531
Treponema vincentii ATCC 35580	ACYH01000036	1518
Treponema zuelzerae DSM 1903^{T}	FR749929	1532
Turneriella parva DSM 21527^{T}	CP002959	1506
Nostoc flagelliforme Sunitezuoqi	GU810186	1479
Thermosynechococcus elongatus BP-1	BA000039	1491

^{T} Type strain

Group	Species	GI	277		306
Spirochaetaceae (30/30)	Borrelia crocidurae	386859446	RIIADHIKASCFILAD	NF	SVLPSNVGQGYV
	Borrelia recurrentis	203287678	----------------	--	------------
	Borrelia duttonii	203284140	----------------	--	------------
	Borrelia turicatae	119953017	----------------	--	A-----------
	Borrelia hermsii	187918093	----------------	--	A-----I-----
	Borrelia spielmanii	224534195	---S--V--A------	SS	GIF---------
	Borrelia afzelii	216264058	---S-----A------	SS	G-F---L-----
	Borrelia bissettii	343127539	---S--V--A------	SS	V-F---L-----
	Borrelia burgdorferi	387827136	---S--V--A------	SS	V-F---L-----
	Borrelia sp. SV1	225552345	---S--V--A------	SS	V-F---L-----
	Borrelia valaisiana	224532246	---S--V--A------	SS	V-F---L-----
	Borrelia garinii	386853627	---S--V--A------	SS	V-S---L-----
	Spirochaeta thermophila	386347103	---S-----AT---G-	ER	G-T---------
	Spirochaeta africana	383791404	---S----TAV-LIG-	EA	G-V---L-----
	Spirochaeta smaragdinae	302339632	------VRTAV---G-	ER	G-K---L----I
	Sphaerochaeta coccoides	330837065	-------RT-V---G-	QK	G-A--------I
	Sphaerochaeta globosa	325971945	--L----RT-V---G-	QR	G-A--------I
	Sphaerochaeta pleomorpha	374317032	------VRT-V---G-	PK	GMA--------I
	Treponema caldaria	339499545	---C--GR-AT---G-	PK	A-S-----A---
	Treponema denticola	325474209	------VRTA----G-	PK	TT----I-A---
	Treponema brennaborense	332297434	---C--VR-AT---G-	PK	G-------A---
	Treponema phagedenis	320536417	---C--VR-AT---S-	PK	--A-----A---
	Treponema primitia	374814755	---S--VR--T-V-G-	PK	A-T-----A---
	Treponema azotonutricium	333995407	---S--LR--S---G-	PK	A-S-----A---
	Treponema saccharophilum	381179175	------SR--V---G-	QK	G-S-----A---
	Treponema paraluiscuniculi	338706964	--VC---R-AT---G-	PV	P-R-----A---
	Treponema pallidum	15640001	--VC---R-AT---G-	PV	P-R-----A---
	Treponema vincentii	257456350	---C---R-ATV--G-	PK	A-V-----A---
	Treponema succinifaciens	328947373	------SRSAV---G-	QK	G-S-----A---
	Treponema sp. JC4	384108417	------TRS-V---G-	QR	G-T-DR--A---
Other Spirochaetes (0/18)	Leptonema illini	374585996	-VLT--ARS-T-A-S-		GI--D-T-R---
	Turneriella parva	392404112	-VLT---RTLT-AMS-		GIF---ESR---
	Leptospira interrogans	458567507	-VVT---RSVL-SIG-		GIY-DRT-R---
	Leptospira santarosai	421084391	-VVT---RSVL-SIG-		GIY-DRT-R---
	Leptospira noguchii	464177692	-VVT---RSVL-SIG-		GIY-DRT-R---
	Leptospira borgpetersenii	116331894	-VVT---RSVL-SIG-		GIY-DRT-R---
	Leptospira kirschneri	464212977	-VVT---RSVL-SIG-		GIY-DRT-R---
	Leptospira inadai	398342628	-V-T---RSAL-AVS-		GIY-DRT-R---
	Leptospira weilii	417779829	-VVT---RSVL-SIG-		GIY-DRT-R---
	Leptospira licerasiae	359686873	-V-T---RSVL-TVS-		GIY-DRT-R---
	Leptospira alstoni	463328226	-VVT---RSVL-SIG-		GIY-DRT-R---
	Leptospira biflexa	183220780	-V-T--SRSVF-S-G-		GIY-DRT-R---
	Leptospira meyeri	408793986	-V-T--SRSVF-S-G-		GIY-DRT-R---
	Brachyspira murdochii	296126160	NL----LR-LV-V--E		GCV---E-R---
	Brachyspira intermedia	384207866	NL----LR-LV-VISE		GCT---E-R---
	Brachyspira hampsonii	429123255	NL----LR-LV-VISE		GCT---E-R---
	Brachyspira hyodysenteriae	225620397	NL----LR-LV-VISE		GCT---E-R---
	Brachyspira pilosicoli	431808332	NLL---LR-LV-V--E		GCK---E-R---
Other Bacteria (0/>250)	Rhodospirillum photometricum	384260715	-V----LR----LI--		G---A-E-R---
	Brucella melitensis	17987072	-V----LR--S-LI--		G-----E-R---
	Rhizopus oryzae	384490438	K-V---FR-M--LIG-		G-I-----R---
	Shewanella baltica	353535042	-V-----RSCA-LI--		G-M---E-R---
	Vibrio splendidus	84390150	-V-----RSCS-LI--		G-M---E-R---
	Jonquetella anthropi	260654150	KV-S---RSV--M---		GI----E-P---
	Neisseria weaveri	343969894	KV-----R-CS-LI--		G-----E-R---
	Leptotrichia goodfellowii	262038170	KV-----R--V-LIG-		G-----E-R--I
	Pelobacter carbinolicus	116878605	-V----SR-TA-LI--		G-----E-R---
	Clostridium bartlettii	164688540	---T---R-MS-MI--		GI----E-R---
	Granulicella mallensis	374312679	------AR--T-LIS-		G---A-E-R---
	Deferribacter desulfuricans	291279844	-V----AR-TT-LIS-		G-----E-R---

Supplemental Figure 01: A partial sequence alignment of conserved region of Alanyl-tRNA synthetase showing a 2 amino acid insertion specific for the family *Spirochaetaceae*.

Group	Species	GI	256	Insert	297
Spirochaetaceae (30/30)	Borrelia burgdorferi	387827445	PLALLYKERDYSRVSN	DVADSNIS	VTKLLGDVEGKNVFMSDD
	Borrelia garinii	51598795	----------------	--------	------------------
	Borrelia sp. SV1	496158147	----------------	--------	------------------
	Borrelia valaisiana	492960169	----------------	--S-----	------------------
	Borrelia bissettii	343127845	----------------	--V-----	------------------
	Borrelia spielmanii	493478988	----------------	--N-----	------------------
	Borrelia afzelii	384207032	---------------S	-IT-----	------------------
	Borrelia hermsii	187918407	------------K-TH	N-N-----	------------------
	Borrelia recurrentis	203287984	------------K-TH	N-T-----	------------------
	Borrelia duttonii	203284450	------------K-TH	N-T-----	------------------
	Borrelia crocidurae	386859775	------------K-TH	N-T-----	------------------
	Borrelia turicatae	119953329	------------K-TH	N-N-----	------------------
	Spirochaeta thermophila	386346567	---VI-------K--T	-ASN----	SL----N-Q--T---A--
	Spirochaeta smaragdinae	302337090	---------------R	-ASS---T	NIR-----K--I---A--
	Sphaerochaeta pleomorpha	374317254	---M--------I--K	-AKH---K	SIN-----H--T-LIA--
	Sphaerochaeta globosa	325971852	---M--------I--K	-AKH---K	SIN-----S--T-LIA--
	Sphaerochaeta coccoides	330837008	---M--------M--R	-AKH---K	SIN-----K--T-LIA--
	Treponema caldaria	339498915	--------------TQ	NAL-N--A	EI----N-Q--T---A--
	Treponema primitia	333996912	--------------TK	-AM-N---	EI----N-S--T---A--
	Treponema azotonutricium	333994388	------------K--R	NALEN--A	EI----N-RD-T---A--
	Treponema denticola	42528223	---MI-------V-TQ	NAKQ---I	SI----------A-IA--
	Treponema succinifaciens	328948985	---MI-------I-TQ	-AKNT--K	SI------K--VA-LA--
	Treponema saccharophilum	488791143	---MI-------V-TQ	-AKNT--K	SI------K--VA-LA--
	Treponema brennaborense	332297752	---MI-------V-TQ	NAK-T--K	SV------H--AA-LA--
	Treponema sp. JC4	496394911	---MI-------I-TQ	NANET--K	SV------K--VA-LA--
	Treponema pallidum	15639286	---MI--V----V-AQ	NAKQ---V	EIN--------TA-IA--
	Treponema vincentii	493197584	---MI-------V-TQ	NAKQT--V	NIN-----N--TA-LA--
	Treponema phagedenis	488785632	---MI-------VITQ	NAKQ---I	SIN-----K--TA-IA--
Other Spirochaetes (0/18)	Brachyspira hyodysenteriae	225216214	DI-IID-R--RANECE		-MNII---N--DAIII--
	Brachyspira murdochii	296125819	DI-IID-R--RANECE		-MNII---N--DAIII--
	Brachyspira intermedia	384208208	DI-IID-R--RANECE		-MNII---N--DAIII--
	Leptonema illini	488860053	G--IID-R-PKAN-AE		IMHVI---KD--CILY--
	Turneriella parva	392404244	T--IID-R-PKAN--E		-MHVI-EI----CILL--
	Leptospira wolffii	514354150	T--IID-R-PKAN--E		-MNVI--I----CILL--
	Leptospira inadai	498101250	T--IID-R-PKAN--E		-MHVI-EI----CILL--
	Leptospira interrogans	446935455	S--IID-R-PKAN--E		-MNVI-EI----CILL--
Other Bacteria (0/>250)	Alkaliphilus oremlandii	158321665	-I-IID-R-PKAN-AE		-MNII--I-----ILI--
	Thermoanaerobacter wiegelii	345018833	---IID-R-PKAN-AE		IMNII---R--K-ILV--
	Clostridium leptum	490600469	---IID-R-QRAN-CE		-MNII---K--K-ILV--
	Peptoniphilus harei	492764066	-I-IIE-R-PKAN--E		-MNII------S-ILV--
	Dictyoglomus thermophilum	206900384	---II--R-PAPE-AE		-EEII-----RD-IIC--
	Chthonomonas calidirosea	512552276	-I-IIA-R-PAPNKVE		IMEII------TCV-I--
	Lactobacillus oris	489810073	-I-IID-R-PKAN-AQ		-MNII---K--TCI-I--
	Bacillus bataviensis	494143805	---IID-R-PRAN-AE		IMNII---Q--KCILI--
	Enterococcus moraviensis	498461095	-I-IID-R-PKAN-AE		-MNII-H----TCVLI--
	Facklamia ignava	493751806	-I-IVD-R-PKAN-AE		-MHIV---K--RCIII--
	Thermotoga thermarum	338730625	---I-D-R-PADNMAE		-VNVI---K--IAL-F--
	Thermosipho africanus	490205698	---I-D-R-PNDN-AE		ILNII-E----TAIIV--
	Helicobacter mustelae	291276443	D-VIVD-K-EKAN--E		-MNII-E-Q--D-ILV--
	Wolinella succinogenes	34557951	D-VIVD-K-EKAN--E		-MNII---Q--D-ILV--
	Caminibacter mediatlanticus	494738649	DMVIVD-R-EKAN--E		-MNII---K--D-ILI--
	Stigmatella aurantiaca	310823075	---IID-R-PRPNS-E		-MN-I---K--DAILV--

Supplemental Figure 02: A partial sequence alignment of conserved region of Phosphoribosylpyrophosphate synthetase showing a 8 amino acid insertion specific for the family *Spirochaetaceae*. Sequences from some members of the class Clostridia which have additional 4 amino acid and 1 amino acid inserts in this region are not shown.

Group	Species	GI	340		373
Spirochaetaceae (30/30)	Treponema pallidum	15639201	RENGGTIPGIRADKTEE		YLQGILNRLVLPGSLYL
	Treponema paraluiscuniculi	338706183	-----------------		-----------------
	Treponema vincentii	257456912	-----S-----T-----		---R-----I-------
	Treponema brennaborense	332298893	-----S-----T-----		---KV------------
	Treponema phagedenis	320538146	-----S-----T-----		---R-------------
	Treponema saccharophilum	381181181	-----S-----I-----		-M-RV----I-------
	Treponema denticola	42526299	-----S-----T-----		---K-----I-------
	Treponema succinifaciens	328949257	-----S-----T-----		---KV----I-------
	Treponema sp. JC4	384108321	-----S---V-T-----		--TK-----------F-
	Treponema primitia	374814283	-----S-----T--I--		--TK----I--------
	Treponema azotonutricium	333996145	-----S-----SERI--		--TK----I--------
	Treponema caldaria	339499042	-----S-----T--I--		--MA----II-------
	Spirochaeta smaragdinae	302337497	-----S-----SE-M--		--ST----I----AIF-
	Spirochaeta africana	383789808	-----SV----SE-M--		-FTK----II---A-F-
	Spirochaeta thermophila	386346286	--H--SV--V--E-L--		-FTN----II---A-F-
	Sphaerochaeta pleomorpha	374316512	-----SV--V-SE-L--		--SKV---V-----IF-
	Sphaerochaeta globosa	325972641	-----SV--V-TE-L--		--TRV---I------F-
	Sphaerochaeta coccoides	330837635	-----SV--V-TE-L--		--TR----I----AIF-
	Borrelia hermsii	187918364	-K--------K--EM-G		--DK-M--TLFS--IF-
	Borrelia recurrentis	203287942	LK--------K--EM-T		--DK-M--TLFS--IF-
	Borrelia duttonii	203284408	LK--------K--EM-A		--DK-M--TLFS--IF-
	Borrelia turicatae	119953287	-K--------K--EM-G		--DR-M--TLFS--IF-
	Borrelia valaisiana	224531848	-K--------KS-EM-K		--DE-M-KTLFS--IF-
	Borrelia garinii	219684447	-K--------KS-EM-K		--DE-M-KTLFS--IF-
	Borrelia burgdorferi	195941635	-K--------KS-EM-K		--DE-M-KTLFS--IF-
	Borrelia spielmanii	224534664	-K--------KS-EM-K		--DE-M-KTLFS--IF-
	Borrelia afzelii	111115327	-K--------KS-EM-K		--DE-M-KTLFS-AIF-
	Borrelia sp. SV1	225551928	-K---A----KS-EM-K		--DE-M-KTLFS--VF-
	Borrelia bissettii	343127805	-K---V----KS-EM-K		--DE-M-KTLFS--IF-
Other Spirochaetes (0/18)	Leptospira interrogans	328488365	KKY--F-----P-SHTK	E	-IEKV---IT---AMF-
	Leptospira borgpetersenii	317160322	KKY--F-----PGSHTK	E	-IEKV---IT---AMF-
	Leptospira inadai	188038371	KKY--F-----PGSHTK	E	-IEKV---IT---AMF-
	Leptospira noguchii	188038335	KKY--F-----PGSHTK	E	-IEKV---IT---AMF-
	Leptospira weilii	188038453	KKY--F-----PGSHTK	E	-IEKV---IT---AMF-
	Leptospira santarosai	188038493	KKY--F-----PGSHTK	E	-IEKV---IT---AMF-
	Leptospira meyeri	188038455	KKY--F-----PGSHTK	E	-IEKV---IT---AMF-
	Leptospira alstoni	463330366	KKY--F-----PGSHTK	E	-IEKV---IT---AMF-
	Leptospira kirschneri	328488641	KKY--F-----PGSHTK	E	-IEKV---IT---AMF-
	Leptospira broomii	398348408	-KYN-F-----PGSHTK	E	-IEKV---IT---AIF-
	Leptospira licerasiae	359687069	-KYN-F-----PGSHTK	E	-IEKV---IT---AVF-
	Leptospira biflexa	183221328	KKY--F---V-PGSQ-K	E	MIEK----IT--GALFL
	Leptospira meyeri	408790999	KKY--F---V-PGSQ-K	D	MIEK----IT--GALFL
	Leptonema illini	374587569	KKY--F-----PGANTK	E	-VEKT---II---AVF-
	Turneriella parva	392404103	KK---Y---V-PGAHT-	E	T-ER----IT-S-AIF-
	Brachyspira pilosicoli	434383102	KKQ--F---Y-PGTQTA	E	--KTV-S-ITIG---F-
	Brachyspira hampsonii	445062100	KKS--F---Y-PGTQTA	E	--KTV-S-ITIG--IF-
	Brachyspira murdochii	296126885	KKS--F---Y-PGTQTA	E	--KTV-S-ITIG--IF-
	Brachyspira hyodysenteriae	225621047	KKS--F---Y-PGTQTA	E	--KTV-S-ITIG--IF-
	Brachyspira intermedia	384209992	KKS--F---Y-PGTQTA	E	--KTV-S-ITIG--IF-
Other Bacteria (0/>250)	Chloroflexus aggregans	219849867	QR---F-----PG-KT-	E	--MKV---IT-A-A-F-
	Streptomyces auratus	398784950	KKY--F------GRPTA	E	--SYV---ITW------
	Persephonella marina	225850755	-KS-AF------GSQTA	E	--NHV-S-I-F---IF-
	Peptoniphilus lacrimalis	282882522	QQ---F-----PG-PTS	E	---RS--------AIA-
	Odoribacter laneus	374386792	KK---F---VKPG-KTM	E	F-DT-MS-IT----IF-
	Bacillus methanolicus	387588912	KKQ--Y-----PG-NTQ	E	--TRV-Y--T-----F-
	Thermotoga lettingae	157363462	-KY--F-----PG-ST-	E	-I-R-I--VTFM-A-F-
	Synechococcus elongatus	56751892	KKM-SS---V-PGRATS	Q	-V--V----TIL-AVF-
	Chlorobaculum parvum	193211871	-RQ--F---V-PG-ST-	E	FIDN--T-IT---AIA-
	Campylobacter jejuni	57790395	KKQ--F-----PGEGTS	N	--NEVAS--T-S--I--
	Dictyoglomus thermophilum	206900395	KKY--F---V-PG-PT-	E	---RT----NFF-GVF-
	Thermus aquaticus	218294774	--Y--F-----PGEPTV	K	F-EH-VS--T-W-A-F-
	Dehalococcoides ethenogenes	57234767	QRQ--FV----PG-MTD	Q	--S-VIG-ITWA-A-F-
	Selenomonas sputigena	260886896	KKY--F-----PG-PT-	E	--DTVMT-IT-A-AFF-
	Nodularia spumigena	119511161	KKM-SS-----PG-ATS	E	--ERVI---TFL-AIF-

Supplemental Figure 3: A partial sequence alignment of conserved region of Preprotein translocase secY showing a one amino acid deletion specific for genera *Spirochaetaceae*.

Group	Organism	Accession	137		176
Spirochaetaceae (30/30)	Treponema vincentii	257457828	RMYLRWAERRGFSVETLDELEA		DEGLKSITLQIRGDYAYG
	Treponema brennaborense	332297634	---V--------K---V-M---		EG-I--V----S-E----
	Treponema sp. JC4	384108691	---------H-YKT-VV-----		EG-I----I--T---V--
	Treponema primitia	333998623	------------AA-E--R-D-		EG-I--A-CR-D------
	Treponema azotonutricium	333995193	------------TLTEV-R---		EG-I--A-VK-E------
	Treponema phagedenis	320535245	---------NDYKT----M---		-G-I-----KVS-E--F-
	Treponema denticola	325474491	------C-S---KT--I-VV--		EG-I--A-F--S-EF-F-
	Treponema saccharophilum	381180503	---I-----H-YKL--V-LQ-D		EG-I--T-I--E---V--
	Treponema paraluiscuniculi	338706540	---T------S-C-HIV-L--S		EG-V--V--K-C-SH-F-
	Treponema pallidum	15639564	---T------S-C-HIV-L--S		EG-V--V--K-C-SH-F-
	Treponema succinifaciens	328948955	---Q-----H-YK--V-SQE--		EG-----NMK-S-P-V--
	Treponema caldaria	339499853	-------------I-EV-M---		EG-I--V-VK-E------
	Spirochaeta smaragdinae	302338432	---T------E---Q---L---		EG-V--V----N--FVF-
	Spirochaeta thermophila	386347018	------L--K---TQIV-MV-D		EG-I--V-VEVK----F-
	Spirochaeta africana	383790275	---S--V-Q--YTSTV--L---		EG-V--V-IEVV-P--F-
	Sphaerochaeta coccoides	330836506	------C--N--KS-I--MQ-D		EG-I--T-IRVS-Q--F-
	Sphaerochaeta globosa	325971169	-----YC--N--KS-V--L--D		EG-I--A-IRVS-P-SF-
	Sphaerochaeta pleomorpha	374314901	-----YC--N--KS-I--LQ-D		EG-I--V-IRVS-Q-SF-
	Borrelia hermsii	187917953	-I---YS----YKT-LI-L---		EG-I--V-IE-K-K----
	Borrelia duttonii	203284000	-T---YS----YKI-LI-L--S		EG-I--V-IE-K-K----
	Borrelia crocidurae	386859303	-T---YS----YKI-LI-L--S		EG-I--V-IE-K-K----
	Borrelia recurrentis	203287543	-T---YS----YKI-LI-L--S		EG-I--V-IE-K-K----
	Borrelia turicatae	119952877	-I---YS--H-YKT-LI-L---		EG-I--V-IE-K-E----
	Borrelia burgdorferi	216264821	---S-Y---KKYKT-LI-L---		EG-I--V-IE-K-E----
	Borrelia afzelii	111114896	---S-Y---KKYKT-LI-L---		EG-I--V-IE-K-E----
	Borrelia garinii	51598335	---S-Y---KKYKT-LI-L---		EG-I----IE-K-E----
	Borrelia spielmanii	224534879	---S-Y---KKYKT-LI-L---		EG-I--V-IE-K-E----
	Borrelia bissettii	343127396	---S-Y---KKYKT-LI-L---		EG-I--V-IE-K-E----
	Borrelia sp. SV1	225552175	---S-Y---KKYKT-LI-L---		EG-I--V-IE-K-E----
	Borrelia valaisiana	224532320	---S-YV--KKYKT-LI-L---		EG-I--V-IE-K-E----
Other Spirochaetes (0/18)	Brachyspira hyodysenteriae	225621552	---V-FC--H--T---T---PG	D	EA-I-Q-SFYVQ-L----
	Brachyspira intermedia	384208365	---V-FC--H--T---T---PG	D	EA-I-Q-SFYVQ-L----
	Brachyspira pilosicoli	300869767	---V-FC--H--T---T---PG	D	EA-I-Q-SFYVQ-L----
	Brachyspira murdochii	296127692	---V-FC--H--T--IT---PG	D	EA-I-Q-SFYVQ-L----
	Brachyspira hampsonii	445064386	---V-FC--H--T---T---PG	D	EA-I-Q-SFYVQ-L----
	Leptonema illini	374587319	---T--F-K--YD-----FQ--	D	EA-I--AS-LVK-EN-F-
	Turneriella parva	392403518	-A-M-YG-KA--N--LI-YQ-G	E	EA-I-NA-ILVS-PN---
	Leptospira interrogans	464380401	---T-YF-KK-YQYSLI-IQAG	D	GA-I-NA--HVI--F-F-
	Leptospira kmetyi	398338096	---M-YF-KK-YQYSLI-IQAG	D	GA-I-NA--HVI--F-F-
	Leptospira kirschneri	421130238	---T-YF-KK-YQYSLI-IQAG	D	GA-I-NA--HVI--F-F-
	Leptospira interrogans	459450376	---T-YF-KK-YQYSLI-VQAG	D	GA-I-NA--HVI--F-F-
	Leptospira noguchii	464368208	---T-YF-KK-YQYSLI-VQAG	D	GA-I-NA--HVI--F-F-
	Leptospira borgpetersenii	421100863	---I-YF-KK-YRYSLI-IQAG	D	GA-I-NA--HVA--F-F-
	Leptospira weilii	417778830	---I-YF-KK-YQYSLI-IQAG	D	GA-I-NA--HVI--F-F-
	Leptospira licerasiae	359690548	----KYFDKK-YQYSVV-FQ-G	D	GA-I-NA-IHVI--F---
	Leptospira inadai	398344616	----KYFDKK-YQYSVV-FQ-G	E	GA-I-NA-IHVI--F---
	Leptospira broomii	398349614	----KYFDKK-YQYSVV-FQ-G	E	GA-I-NA-IHVI--F---
	Leptospira santarosai	464372999	---V-YF-KK-YKYSLI-VQAG	D	GA-I-NA--HVV--F-F-
Other Bacteria (0/>250)	Symbiobacterium thermophilum	51891274	---V--------K--E--R-DG	D	EA-I--V-IAV--P----
	Clostridium perfringens	168215798	---T--C-KK-Y-L--I-Y-PG	D	EA-V--V--KVK-EF---
	Bacillus mojavensis	398308472	---T--------K-----Y-PG	D	EA-I--V--L-K-HN---
	Paenibacillus vortex	315649817	---T----K---K-----Y-PG	D	EA-I--V--L-K-YN---
	Desulfurivibrio alkaliphilus	297568438	--------KK---T-I--H-PG	D	EA-T--V-VLVK-RW---
	Escherichia coli	331674376	--------S---KT-IIE-S-G	E	VA-I--V-IK-S------
	Planctomyces maris	149177843	------C-----N--L--RSD-	E	EA-IR-A-IR-E------
	Thermanaerovibrio velox	365873548	---V-----N--K-KL----PD	Q	EA-I--V-I-VS-E----
	Fervidobacterium nodosum	154250427	---M-----K-----LV-YQ-G	E	EA-I--A--Y-K--F---
	Methylobacillus flagellatus	91775149	-----YC-NK--K--V-E-S-G	D	VA-I--AS-K-S------
	Magnetococcus marinus	117924338	-----YC-KS--KT-EV-Y-AG	D	EA-I--V-IKVE------
	Dehalococcoides ethenogenes	57234653	--FM----KK--GM-V--QSPG	E	EA-I--A----E-E----
	Thermomicrobium roseum	221633612	-------Q-A--AA-VV-L--G	E	EA-I--A-VEV--P----
	Sphaerobacter thermophilus	269836751	-------SN---QAQV--VT-G	E	EA-I--A-IE---P----
	Frankia alni	111220808	---------H-YPT-VF-TS--	E	EA----A-F-VKAP----
	Veillonella atypica	303229877	--------KT-----LM--QPG	D	EA-I--A-FL-K-EN-F-
	Leptotrichia hofstadii	260889274	---D---N-HD-K--I--S-AG	E	EA-I--V--N-K-N----
	Chloroflexus aurantiacus	163846312	---T-------YT-NLI-MS-G	E	EA-I--A-IE---P----
	Akkermansia muciniphila	187734757	---I--C-------TY-ESTDG	D	-A-IR-V--KVD-E----

Supplemental Figure 4: Partial sequence alignment of conserved region of Peptide chain release factor 2 prfB showing a one amino acid deletion specific for genera *Spirochaetaceae*.

Group	Organism	Accession	720		751
Spirochaetaceae (30/30)	Borrelia burgdorferi	224532424	ATHFHELSSINH		QAFINLSMKIEKQGNDLVFL
	Borrelia sp. SV1	225551681	------------		K-------------------
	Borrelia spielmanii	224534129	--------A---		E-------------------
	Borrelia garinii	51599049	--------A---		K-------------------
	Borrelia valaisiana	224531774	--------A---		K----------------I--
	Borrelia bissettii	343128094	------------		K-------------S-----
	Borrelia afzelii	216263544	--------A---		K--------------E----
	Borrelia hermsii	187918651	--------A---		DS-V-------R---E-I--
	Borrelia crocidurae	386860036	--------A---		DS-V-------Q--DE-I--
	Borrelia duttonii	203284693	--------A---		DS-V-------Q--DE-I--
	Borrelia recurrentis	203288226	--------A---		DS-V-------Q--DE-I--
	Borrelia turicatae	119953574	--------A-K-		DS-V-------R--DE-I--
	Spirochaeta africana	383790736	---Y-Q--M-E-		PD-T---LSVVEN-S-I---
	Spirochaeta thermophila	386346893	-------TK-T-		P-VF----AVREDRDGI---
	Spirochaeta smaragdinae	302338534	-------TELQ-		DSIQK-RLMVKEE-DRVI--
	Sphaerochaeta globosa	325971227	---Y---AR-DT		S-LQL-TLQVSEM-GQVR--
	Sphaerochaeta coccoides	330836559	---Y---TMLDT		SQIQL-TLEVAQKARKII-M
	Sphaerochaeta pleomorpha	374314845	---Y---AQLDT		SLVQL-TLQVLEK-GEIL-V
	Treponema caldaria	339499969	---Y----R-E-		PRLA-R--EVLE-DGRIL--
	Treponema primitia	333997850	---Y---ALLS-		PHLA-R--EVLN--GEI---
	Treponema succinifaciens	328948686	---Y---TRLE-		KSLKR-C-AVAEN-S-I---
	Treponema azotonutricium	333994679	---Y----Q-S-		PRMS-R--EVSDNNGEI---
	Treponema denticola	42528103	---Y---TRLE-		EKI---KLDVLEAEGKI---
Other Spirochaetes (0/18)	Leptonema illini	374584203	---Y---TALEE	R	TG-V--T-DVRESDGRVI--
	Leptospira licerasiae	359689895	---Y---TELSR	L	PGVW--H-ETVEKDDKVI--
	Leptospira broomii	398347395	---Y---TELSR	L	PGVW-IH-ETLEKEDKVL--
	Leptospira inadai	398345415	---Y---TELSR	L	PGVW-IH-ETLEKEDKVL--
	Leptospira biflexa	183220240	---Y---TELEK	G	AGIF--YLDTFEKDGEIL--
	Leptospira meyeri	408793270	---Y---TELEK	G	NGIF--YLDTFEKEGEIL--
	Leptospira alstoni	463330954	---Y---TELSR	L	SGIF--YLETLEKEDKVL--
	Leptospira weilii	464295206	---Y---TELSR	L	SGIF--YLETLEKEDKVL--
	Leptospira alexanderi	398332805	---Y---TELSR	L	SGIF--YLETLEKEDKVL--
	Leptospira borgpetersenii	418738801	---Y---TELSR	L	SGIF--YLETLEKEDKVL--
	Leptospira kirschneri	464409192	---Y---TELSR	L	GGIF--YLETLEKEDRVL--
	Leptospira noguchii	410940934	---Y---TELSR	L	GGIF--YLETLEKEDRVL--
	Leptospira interrogans	458653907	---Y---TELSR	L	GGIF--YLETLEKEDRVL--
	Leptospira santarosai	421110175	---Y---TELSR	L	GGIF--YLETLEKEDKVL--
	Brachyspira pilosicoli	434380887	---Y---TMLED	L	EGVK-YKVLV-EYKDEII-M
	Brachyspira hyodysenteriae	225620061	---Y---TMLED	L	EGVK-YKVLV-EYKDEII-M
	Brachyspira murdochii	296127255	---Y---TMLED	L	EGVK-YKVLV-EYKDEII-M
	Brachyspira hampsonii	429123686	---Y---TMLED	L	EGVK-YKVLV-EYKDEII-M
	Brachyspira intermedia	384209630	---Y---TMLED	L	EGVK-YKVLV-EYKDEII-M
	Turneriella parva	392403868	---YS--AHLIS	QE	RGIAG-TVSVVEKDGHV--M
Other Bacteria (0/>250)	Peptoniphilus indolicus	350566699	---Y---TDLSN	IY	DEVE--TIAVD---E-I---
	Desulfosporosinus meridiei	354561670	---Y---TQLQD	NF	PGLV--HVGVKER-E-I---
	Methylosinus trichosporium	296444520	-------TQLTK	RL	PRLV--T--VKDHAGEV---
	Brucella melitensis	17988084	------MTALSE	KL	ERLS-VT-RVKEWD--VI--
	Caulobacter crescentus	16124268	---Y---ATLET	RM	AFVS---LRAKEWNG-----
	Rickettsia australis	383501920	---Y---TVM-N	FL	P-LQ-YTIA--ES-K-IL--
	Rickettsia akari	157825535	---Y---TVM-N	FL	P-LQ-YTIA--ES-K-IL--
	Thermobaculum terrenum	269926487	---Y---T-LEE	FL	PRVK-FR-EVLEE---V---
	Pelosinus fermentans	392960659	---Y---TELAD	YH	KTVK-Y-VAVKER-S-V---
	Prochlorococcus marinus	33862352	---Y---NGLSQ	EL	TNVA-FQVLV-ET-D-----
	Dictyoglomus turgidum	217967464	---Y---TELEK	EL	KHLK---VAVQEK-K-II--

Supplemental Figure 5: A partial sequence alignment of DNA mismatch repair protein mutS showing a two amino acid deletion specific for genera *Spirochaetaceae*.

Group	Species	GI	494		520
Spirochaetaceae (30/30)	Treponema paraluiscuniculi	338706271	DQHAAHERIIFDTLQRNLGTA		QILLIP
	Treponema pallidum	15639295	---------------------		------
	Treponema denticola	325474471	---------L-EG-KKS--PS		-E----
	Treponema vincentii	257457433	------------Q-KH-T-GV		-E----
	Treponema sp. JC4	384109565	----V----L--K-MN-Q-KS		-P----
	Treponema phagedenis	320535178	---------L-NE-KQS--IS		-E----
	Treponema saccharophilum	381181462	----V----L--EMMGAERI-		-T----
	Treponema succinifaciens	328948043	--------M-YNQIMAEA-QK		-S--V-
	Treponema brennaborense	332298473	---------LYNAFIAQA-RK		-A--V-
	Treponema primitia	333997998	---------LY-RFLSKPIL-		-E--V-
	Treponema caldaria	339500256	---------L--S-TTQPIQN		-D----
	Borrelia garinii	219685877	----V--K--YEK-RNSKK-V		-K----
	Borrelia spielmanii	224534215	----V--K--YEQ-RNSKK-V		-K----
	Borrelia valaisiana	224531620	----V--K--YEQ-RNSKK-V		-K----
	Borrelia bissettii	343127530	----V--K--YEK-RNSKK-I		-K----
	Borrelia afzelii	111115035	----V--K--YEK-RNSKK-I		-K----
	Borrelia crocidurae	386859437	----L--K--YQ--INSEK-I		-K--V-
	Borrelia recurrentis	203287669	----L--K--YQ--INSEK-I		-K--V-
	Borrelia duttonii	203284131	----L--K--YQ--INSEK-I		-K--V-
	Borrelia burgdorferi	387827127	----V--K--YEK-RNSKKNV		-K--V-
	Borrelia turicatae	119953008	----L--K--YQ--INSEKIT		-K----
	Borrelia sp. SV1	225551982	----V--K--YEK-RNSKKIV		-K--V-
	Spirochaeta smaragdinae	302338864	---------LYEQFVAGASRK		-Q----
	Spirochaeta africana	383790648	-M--G---LRY--- -HA-D-		-K-VV-
	Sphaerochaeta pleomorpha	374314579	---------L--EI -GKKEV		-A-M--
	Sphaerochaeta globosa	325971464	--------L---EV -EKKNI		-Q-M--
Other Spirochaetes (0/18)	Leptospira inadai	398342440	---T-----RYEEVLKK-KRK	NYGI	-P--T-
	Leptospira broomii	398348019	---T-----RYEEVLRK-KRK	NYGI	-P--T-
	Leptospira licerasiae	359687950	---T-----RYEEVLRK-KKK	NYGI	-P--T-
	Leptospira borgpetersenii	421099993	---T-----RYEEVLRK-ERK	NYGI	-P--T-
	Leptospira santarosai	469839967	---T-----RYEEVLRK-EKK	NYGI	-P--T-
	Leptospira weilii	417781228	---T-----RYEEVLRK-EKK	NYGI	-P--T-
	Leptospira meyeri	408790829	---T-----RYEEVLRD-KSK	AYKS	-S--T-
	Leptospira biflexa	183221533	---T-----RYEEVLRD-KSK	AYKS	-S--T-
	Leptospira alstoni	463332188	---T-----RYEEVLRK-EKK	NYGI	-P--T-
	Leptospira kmetyi	398336310	---T-----RYEEVLRK-EKK	NYGI	-P--T-
	Leptospira noguchii	470025988	---T-----RYEEVLRK-EKK	NYGI	-P--T-
	Leptospira interrogans	470048520	---T-----RYEEVLRK-EKK	NYGI	-P--T-
	Leptospira kirschneri	463329343	---T-----RYEEVL-K-EKR	NYGI	-P--T-
	Brachyspira hampsonii	445064025	-----Y--LNYERIYKT-MSK	KIEY	EK----
	Brachyspira hyodysenteriae	225619059	-----Y--LNYERIYKT-MSK	KIEY	EK----
	Brachyspira intermedia	384209368	-----Y--LNYERIYKT-MSK	KIEY	EK----
	Brachyspira pilosicoli	431807712	-----Y--LNYERIYKT-MSG	KLEY	EK----
	Brachyspira murdochii	296125279	-----Y--LNYERIYKT-ISK	KIEY	EK----
	Turneriella parva	392404678	---T-----NYERFL-R-AEK	RDMA	-Q-AT-
	Leptonema illini	374587101	---T-----N-EKKRRE-EAR	RFQR	----H-
Other Bacteria (0/>250)	Desulfitobacterium hafniense	89894335	---------NYER-LAEHQNN	PGNS	-M----
	Clostridium thermocellum	256003774	---------R-EE-KEKYARN	ESLA	-Y--T-
	Rhodobacterales bacterium	254466092	--------LVYEK-K-QMAEN	GVAA	-A----
	Phaeobacter gallaeciensis	163739395	--------LVYEK-K-QMAET	GVAA	-A----
	Rhodopseudomonas palustris	90422900	--------LVYEK-KAS-A-N	GVQR	------
	Sagittula stellata	126729914	--------LVYEK-K-QMAEN	GVPA	-A----
	Oceanibulbus indolifex	163745482	--------LVYEK-K-QMNDN	GVAA	-A----
	Agrobacterium radiobacter	222085025	--------LV-EEMRKA-HSK	RLSS	-V----
	Rhizobium etli	86356494	--------LV-EAMRKA-HSK	RLAS	-V----
	Sinorhizobium medicae	150395653	--------LV-E-MRTA-HAR	PVPA	-A----
	Roseobacter denitrificans	110678039	--------LVYEK-K-QMAEN	GVAS	-A----
	Bradyrhizobium japonicum	27382604	---------VYEG-KAS-AAN	GVQR	------
	Nitrobacter hamburgensis	92118635	---------VYEK-KAA-ERD	GVQR	------
	Rhodobacterales bacterium	84684369	--------LVYEK-K-QMAEN	GVAS	-A----
	Agrobacterium tumefaciens	159184423	--------LV-EEMRNA-HSR	RPPS	-V----
	Rhodopseudomonas palustris	283843048	---------VYER-KAS-EAN	GVQR	------
	Silicibacter lacuscaerulensis	260432213	--------LVYEK-KKQMAEN	GVAA	-A----
	Nitrobacter winogradskyi	75676732	---------VYER-KTA-ARD	GVQR	------

Supplemental Figure 6: A partial sequence alignment for DNA mismatch repair protein mutL showing a four amino acid deletion specific for the *Spirochaetaceae*.

Group	Species	GI number	225	Insert	263
Brachyspiraceae (4/4)	Brachyspira pilosicoli	300871449	ATTRPETILADVAIAVHPDDERYAHLTEQ	D	VLILPIVGR
	Brachyspira intermedia	343386875	------------------------N----	-	---------
	Brachyspira hyodysenteriae	225619103	-----------------------------	-	I--------
	Brachyspira murdochii	296127588	----------------------------E	N	---------
Other Spirochaetes (0/44)	Treponema vincentii	257457527	-------L-G-T-V------P--T--VGK		K-V--LT--
	Treponema phagedenis	488788316	-------L-G-T-V----E-------VGK		L-E--LT--
	Treponema azotonutricium	333995473	-------L-G-T-V----E-------IGK		M-K--LTD-
	Treponema denticola	488748126	-------L-G-T------E-P---SIIGK		E----LAN-
	Treponema succinifaciens	328948525	-------L-G-T-V--N-E----KSVVGK		K-K--LT--
	Spirochaeta thermophila	386346193	-------M-G-T-V----G----R--VGK		E--V-L-N-
	Spirochaeta africana	383791644	-------M-G-T-V----E-------VG-		E-E--LT--
	Sphaerochaeta coccoides	330836357	-------MFG---V--N-------AVVGK		K-R--LTD-
	Sphaerochaeta pleomorpha	374314559	-------MFG---V--N------TS-IGT		M-D--LTD-
	Borrelia recurrentis	203288168	-------MFG------N-N----KS-IGR		E--I--AN-
	Borrelia duttonii	203284634	-------MFG------N-N----KS-IGR		E--I--AN-
	Borrelia garinii	490931452	-------MFG-T----N-N----KS-VGK		E-TI-LT
	Borrelia crocidurae	386859975	-------MFG------N-N----KS-IGR		E--I--AD-
	Leptospira inadai	498103604	-------MFG---V-A-------KN-KGA		-VE--LTN-
	Leptospira licerasiae	495868683	-------MFG---V-A-------KS-KGA		E-E--LTD-
	Leptospira wolbachii	505589918	-------MFG---VCA----T---A-KGK		F-Y---A-K
	Leptospira interrogans	446666658	-------M-G---VCAN-E----TS-KDV		--D--LTN-
	Leptonema illini	488861362	-------M-G-T--------A--K--IGK		TCK--F-N-
	Turneriella parva	392401702	---------G-E-L----E----K--KGA		KAKV-FLNK
Other Bacteria (0/>250)	Fusobacterium mortiferum	237736668	--S----M-----V----E----K--VGK		K----L---
	Carboxydibrachium pacificum	254478609	-------M-G---V----E----K--IGK		T----L---
	Thermoanaerobacter tengcongens	20807263	-------M-G---V----E----R--IGK		T----L---
	Clostridiales genomosp.	289449837	-------M-G-T-V---------K--IGK		EV---L-N-
	Acetivibrio cellulolyticus	303239683	-------M-G-T-V---------Q--IGK		MV---L-N-
	Coprothermobacter proteolyticu	206895183	-------M-G-T-----------KNVVGK		HV---L---
	Anaerostipes caccae	167745430	-------M-G-T-V---------T--VGK		M-E--LC--
	Filifactor alocis	291171057	--------VG-T-V----E----R--IGK		M-VV-FI--
	Megamonas hypermegale	291532689	-------MFG-TGV---------SD-VGK		T--------
	Coprococcus catus	291521943	-------L-G-T-V----E----MD-IGK		NV---L---
	Syntrophothermus lipocalidus	297616869	-------M-G-TGV---------R--VGR		YA----I--
	Anaerotruncus colihominis	167770372	-------L-G-T-V---------K--IGK		MVL----NK
	Mitsuokella multacida	255659387	-------MFG-TGV---------KD-VGK		T--------
	Natranaerobius thermophilus	188585735	-------M-G-T-V---------S--IGK		TVM--LMD-
	Thermoanaerobacter ethanolicus	256751768	-------M-G---V--N------KDVVGK		T----L---
	Pelotomaculum thermopropionicu	147677147	-------M-G---V------G--KEMVGK		T----L---
	Ruminococcus torques	153814976	-------L-G-T-----------QDIIGK		NV---L-N-
	Caldicellulosiruptor saccharol	146297043	-------M-G-T-V--N-N----K--IGK		TVV----N-
	Mesorhizobium loti	13471197	-------M-G-T-V---------R--VGK		NVV------
	Asticcacaulis excentricus	241774675	-------M-G-T-V---------QA-IGK		EV----T--
	Gluconacetobacter hansenii AT	296115214	-------M-G-M-V------A---D-VG-		SV---LT--
	Erythrobacter litoralis	85373086	-------M---M-V----S-----SVVGK		HVV--LT--
	Rhodobacter sphaeroides	77462536	-------M-G-TG---N-A-------IGK		EVV--L---
	Magnetospirillum gryphiswalden	144899871	-------M-G-S-V----E----T--VGK		MVR------
	Rhodopseudomonas palustris	192291231	-------M-G-T-V--N-E----T--VGK		HV---L---
	Agrobacterium vitis	222148981	-------M-G-TG----------QSIIGK		HV-------
	Gluconobacter oxydans	58040911	-------M-G---V----E-----DMIGK		TV---LT--
	Moraxella catarrhalis	296112744	-------L-G-S-V------------IGK		TIV---S--
	Thiomicrospira crunogena	78485844	-------MFG-Q-V---------Q--IG-		TIT--L---
	Pseudomonas aeruginosa	254236738	-------L-G-A-V----------K-IG-		FAE------
	Idiomarina loihiensis	56461050	-------M-G--CV--------F---VGK		F-E----N-
	Marinobacter algicola	149378126	-------M-G-T-V---------Q--IGK		FVD--L---
	Shewanella amazonensis	119773927	-------M-G-S-V----E-----S-IGK		EI-----N-

Supplemental Figure 7: A partial sequence alignment of Valyl --tRNA synthetase showing a one aa insert specific for the *Brachyspiraceae*.

			660		703
Brachyspiraceae (4/4)	Brachyspira pilosicoli	300871449	WYIEISKFDLKDESKKEK		TIAVLLYVLEESMAMIHPFMPFITEE
	Brachyspira hyodysenteriae	225619103	------------------		--------------------------
	Brachyspira murdochii	296127588	------------------		--------------------------
Other Spirochaetes (1/44)	Turneriella parva	392401702	---M--VA---D-MRDG		ALYT-YT-FRAALRLL--A-------
	Leptonema illini	488861362	--V-LI-SR-YEKEMTPS	KE	AALQTAF---RSILNVL-----H----
	Leptospira wolbachii	505589918	--L-LT-ARVYGNVSP--	QE	KARL--IS--KK-LGLL----------
	Leptospira biflexa	183219675	--L-LT-ARVYGNVTP--	AK	E-ARQ--VS--KK-LGLL---------
	Leptospira interrogans	488108805	----LV-PRAYGKVSHRS	AE	VAKQ--SD--IRALGLL------L---
	Borrelia turicatae	119953516	-------IN-NSDDINLQ	NM	--SK-IFF-K--LLIM---I------
	Borrelia spielmanii	224534475	-------I--NS-DVNIQ	NM	A-SK--FF-KKALVIL---I--V--K
	Borrelia duttonii	203284634	-------IN-CSDDFDLQ	NI	--SK-IFF-K--LLIM---I------
	Borrelia recurrentis	203288168	-------IN-CSDDFDLQ	NI	--SK-IFF-K--LLIM---I------
	Borrelia hermsii	187918595	-------IN-NSDDINLQ	NM	I-SK-VFF-K--LRIM---I------
	Spirochaeta smaragdinae	302338655	----A--LS-YSDDDD--	NR	AVTL-MQ------RLA---LS-----
	Spirochaeta thermophila	386346193	----SA-LS-NGD--QE-	DR	IVSL--F---QALKLL---L--V---
	Sphaerochaeta coccoides	330836357	----A--HN-ESN-PVVQ	DR	AVSL--DI----LRLL---L----Q-
	Treponema vincentii	257457527	--V-GT-LSY-NGDEA--	NR	ITS---A-----LRLL--VL--V---
	Treponema denticola	42526872	--V-GT-LSF-YGDE---	DR	AAS---A-----LRLL---LA-V---
	Treponema pallidum	189026258	--V-A--CS-QKP--QE-	DR	AAS--CTL---TLRLL---L--V---
Other Bacteria (0/>250)	Oceanobacillus iheyensis	23099516	----MA-LS-YG-DENK-	KT	-RS--AH--DQT-R-L----------
	Paenibacillus larvae	167465172	----F--LS-YGDDEQA-	KK	-QS--V---DHT-RL--------S--
	Gemella haemolysans	241889659	-----A-IS-NG-DEAA-	KT	-KSI-V---DA-LK-L------V---
	Staphylococcus carnosus	224476754	----M--IPMNG-DEAQ-	QV	-RS--S----RT-R-L--Y---V---
	Lysinibacillus sphaericus	169829404	----MA-LP-YG-DEAA-	KT	-RSI-A---DQT-RLL----------
	Geobacillus thermodenitrifican	138896204	----MA-LP-YGDDETA-	KT	-RS--A---DNT-RLL----------
	Anoxybacillus flavithermus	212638460	----MA-LP-YGDDEQA-	KT	-RS--A---DQT-RLL------V---
	Bacillus clausii	56964386	----MA-LA-YG-NEQA-	QA	-RS--AH--DQT-R-L----------
	Paenibacillus curdlanolyticus	304407500	----FA-LN-YGSDAAA-	KA	-QS--A---DRTQRL--------S--
	Staphylococcus aureus	282916924	----M--IPMNGNDEAQ-	QI	-RS--S-T-DNI-R-L------V--K
	Alicyclobacillus acidocaldariu	258511798	----FA-IN-YGDREEK-	RQ	-QS---T--SRVL-LL--YI--V---
	Bacillus pseudofirmus	288553603	----MA-LP-NSDNEEA-	RT	-RS--A---DQT-RLL--I-------
	Macrococcus caseolyticus	222151545	----M--IPMNG-DEVQ-	QM	-RS--A-T-DSI-R-L------V--H
	Exiguobacterium sibiricum	172058142	----MA-LP-NG-DEAA-	LT	-RSI-A-T-DQI-RLM----------
	Granulicatella elegans	260584693	----M--ET-AGNDEAA-	LT	-RSI-V---DQTLRLL--I---V---
	Lactobacillus ruminis	227526685	----MA-E--NGSDE-L-	HN	-QNI-C---DQTLRLM--I---V--K
	Pediococcus pentosaceus	116493039	----M--EI-NG-DEQA-	VN	-QN--A---DQTLRLL--V---V--K
	Streptococcus salivarius	228478388	----LT-EV-YSDNED--	VI	-RS----T-DQILRLL--I---V---
	Anaerococcus hydrogenalis	212697050	----FA-ER-NSDDENK-	ST	VKK------KDMISLL----------
	Thermosediminibacter oceani	302389257	----MA-I--YGDDENA-	KR	-QR--YT---RVLRLL---V------
	Ruminococcus albus	304401635	----LA-NRFNG-DAQA-	KN	VEN--A---TDILK-L----------
	Thermoanaerobacterium thermosa	304316504	----L--PV-YGDDLEA-	KK	-KS--R---DNTLRLL----------
	Moorella thermoacetica	83589396	----LI-PR-YG-NRR-R	QV	AQE--VT--GK-LQLL----------
	Subdoligranulum variabile	282600751	-----C-SR-NSDDAVQA	DT	ARK--V---DKALKLL----------
	Anaerococcus lactolyticus	227486662	----FA-IR-YGDDEDA-	AN	VKK------KSMLILL----------
	Clostridium bartlettii	164686738	-----V-PR-YG-D-EA-	QT	ALY--T----KILKLL--Y-------
	Thermoanaerobacter tengcongens	20807263	----L--PV-YSDNEEA-	KV	-KS--R---DNTLRLL----------
	Anaerococcus prevotii	257066730	----FA-IR-YGDD-EA-	AN	VKK------KNMLVLL----------
	Carboxydibrachium pacificum	254478609	----L--PV-YSDNEEA-	KV	-KS--R---DNTLRLL----------

Supplemental Figure 8: A partial sequence alignment of Valyl --tRNA synthetase showing a two aa deletion in the *Brachyspiraceae*. This deletion is also found in *Turneriella parva*.

Group	Species	Accession	760		793
Brachyspiraceae (4/4)	Brachyspira hyodysenteriae	225620632	IGGLKEKTIAAKRLG	F	IKHIIIPHENIRDLDEIP
	Brachyspira murdochii	296126569	---------------	-	-------Y----------
	Brachysppria pilosicoli	300870590	---------------	-	-------F-K--------
	Brachyspira intermedia	343385315	---------------	-	-------Y----------
Other Spirochaetes (0/44)	Turneriella parva	392403479	-------VL--F--G		-T-V-C-KD-QK-FE---
	Leptonema illini	488861717	V--I---VL--RAA-		-NTVLL-ER-RA-FE---
	Leptospira interrogans	488011445	----R--IV----V-		-HK--Y-KD-LQH-Q---
	Leptospira licerasiae	495870264	----R--IV----V-		VRK--F-SD-KPQ-----
	Leptospira borgpetersenii	488829383	----R--IV----V-		IY---Y-KD-LQH-E---
	Leptospira weilii	488691420	----R--IV----V-		IY---Y-KD-LQH-E---
	Borrelia burgdorferi	387827169	-------I-----S-		VE---V-KA-RV--E---
	Borrelia afzelii	384206750	----R--I-----S-		VE---V-RA-KV--E---
	Borrelia garinii	490931949	----R--I-----S-		VE---V-KA-KV--E---
	Treponema pallidum	15639514	-----------R-G-		--E--M-KA-V-------
	Treponema denticola	42526185	-----------R-N-		--EV---QA-T-------
	Treponema primitia	333996820	-----------R-NK		AQ-----KQ-L-------
	Treponema succinifaciens	328947911	----R---V----NK		--T----KA-V-------
	Treponema azotonutricium	333995321	-----------Q-NK		AR-----KQ-M----D--
	Treponema brennaborense	332298163	----R---V----NK		--S----KA-V----D--
	Treponema paraluiscuniculi	338706484	-----------R-G-		--E--M-KA-V-------
	Treponema phagedenis	320535218	-----------R-N-		--E-----A-L---E---
	Treponema vincentii	257458481	-----------R-N-		--E----AA-----EK--
	Treponema caldaria	339500549	-----------Q-NK		A------KK-L-------
	Spirochaeta smaragdinae	302338652	-----------R-NK		--Q----KA-E---K---
	Spirochaeta thermophila	307719319	-------V-----AR		V-E----KS-EK--E---
	Sphaerochaeta coccoides	330836360	-------IL--R-NK		-QT-LF-KQ-Q----KL
	Sphaerochaeta globosa	325971485	-------VL--R-NK		-DT----QF-K----KL
Other Bacteria (0/>250)	Pasteurella multocida	15603843	-------LL--H-G-		--TV---K--VK--E---
	Haemophilus somnus	170718066	-------LL--H-G-		--TV---K--VK--E---
	Acinetobacter haemolyticus	294649847	-------LL--H-G-		--LVF--QD-V-------
	Mannheimia succiniciproducens	52425899	-------LL--H-G-		--TV---KD-VK--E---
	Actinobacillus succinogenes	152978446	-------LL--H-G-		--TV---KD-VK--E---
	Congregibacter litoralis	88703269	-------LL--R-G-		--TV---K--E---Q-V-
	Legionella pneumophila	54294746	-------LL--H-G-		---V---E--VK--E---
	Haemophilus influenzae	145640434	-------LL--H-G-		--TVL--K--VK--E---
	Pseudomonas putida	170720929	-------LL--H-G-		--TV---E--V---K---
	Grimontia hollisae	262276276	-------LL--H-G-		--TV---K--E---E---
	Photobacterium profundum	90410784	-------LL--H-G-		--TV---K--E---E---
	Hahella chejuensis	83644981	-------LL--H-G-		--TVV--Q--V---K---
	Shewanella denitrificans	91793845	-------LL--H-G-		---VL--K--E---E---
	Aggregatibacter actinomycetemc	293391758	-------LL--H-G-		--TV---KD-VK--ED--
	Aliivibrio salmonicida	209694400	-------LL--H-G-		--TV---KD-E---E---
	Pseudoalteromonas atlantica	109899439	-------LL--H-G-		--TV---K--E---Q---
	Providencia rettgeri	268592034	-------LL--H-G-		--TVL--K--E---E---
	Marinobacter aquaeolei	120554756	-------LL--H-G-		--TV---D--V---K---
	Aeromonas hydrophila	117621379	-------LL--H-G-		--RVL--K--E---E---
	Alteromonas macleodii	239995519	-------LL--H-G-		--TVV--KD-E---E---
	Vibrio angustum	90579276	-------LL--H-G-		--TVL--K--E---E---
	Ferrimonas balearica	308050306	-------LL--H-G-		--RVL--K--E---E---
	Moraxella catarrhalis	296113021	-------LL--H-G-		---VL--KS-E---I---

Supplemental Figure 9: Partial sequence alignment of ATP-dependent protease La showing a one aa insert specific for the *Brachyspiraceae*.

Group	Organism	GI	325		361
Brachyspiraceae (4/4)	Brachyspira pilosicoli	300871379	LTEDAALADYYEAAVKAY	P	KQPKKISNWIMVEVNAYL
	Brachyspira hyodysenteriae	225620599	------------S-----	-	------------------
	Brachyspira murdochii	296126183	------------D-----	-	------------------
Other Spirochaetes (0/41)	Turneriella parva	392404480	--A-RST-L---DVIR-G		APA--A---V---M
	Leptonema illini	488860131	-AR-RET-A-F-KVAELS		GDA--S---VKD--LGII
	Leptospira broomii	498257551	--AEREI-----E-LQVS		GD--RT---VKD--
	Leptospira licerasiae	495869152	--AEREI-----G-L-VS		GDA--T---VKD--
	Leptospira noguchii	490585039	--AEREI---F-E-L-VS		EDA--T---VKD--
	Treponema vincentii	257457942	--AERS--EWF---AVKT		-D---VA--VLA--L-V-
	Treponema denticola	42526091	--SERE--EWF-E-A-KS		SS---CA---LA--L-I-
	Treponema phagedenis	488786306	--AEKE---WF-Q---SA		HD---TA---LA--L-V-
	Spirochaeta africana	383792008	I--EKSG--F-----QHG		AD-GAAAA-FVSDIKKQ-
	Spirochaeta thermophila	386347987	-I-EK-R--FF-E--RRG		VA-STAAA-LAGD-AKL-
	Borrelia recurrentis	203287795	--S-KN-VK-F-E-ALTS		SE---VA---LS--LSV-
	Borrelia duttonii	203284258	--S-KN-VK-F-E-ALTS		SE---VA---LS--LSV-
	Borrelia burgdorferi	216264831	--A-KH-LK-F-E--INS		SD---VA---LS--LSV-
	Borrelia spielmanii	224534315	--S-KH-LR-F-E--INS		SD---V---VLS--LSV-
Other Bacteria (0/>250)	Bacillus selenitireducens	297583061	--QQK-MS-FF-Q-IAQG		GP--QL---L-G------
	Lactobacillus sakei	81429159	--NTKEMS-FF----ANG		AA--QV---L-G------
	Aerococcus viridans	295397188	--QTLEMS-FFD-T---G		AD--QA---L-G-----M
	Anoxybacillus flavithermus	212638109	--LTKEM--FF--T-ANG		AD--LA---L----S---
	Pediococcus acidilactici	270291084	--QTKEMS-FF---IAFD		AD--LVA-YL-GD-----
	Enterococcus gallinarum	257869257	--LTKEMS-FF--TID-G		AE--QA---L-G--S---
	Leuconostoc mesenteroides	227432077	--QTL-MS-F-D-T-ASG		AD--RAA-YLIGD----M
	Listeria seeligeri	289435091	--LTKEMS-FF--TI--G		ADA-QA---L-G--S---
	Geobacillus stearothermophilus	20454922	--LTKEM--FFX-T-ANG		AD--LA---L----SG--
	Oceanobacillus iheyensis	23098221	--ASKQMS-FF-E-TAEG		ADM-QV---L-G--S--M
	Pediococcus pentosaceus	116493113	--QTKEMS-FF--T-AQG		AD--LTA-YL-GD-----
	Weissella paramesenteroides	241896422	--QTL-M-NFFDET-SFG		AD--RTA-YL-GD---F-
	Gemella haemolysans	241890109	--LTKEMS-MF-ET-ALG		AD--LA--YL--D-----
	Acidovorax delafieldii	241766795	--QSK-M-A-F--VA--C		S---LA-----G--SRR-
	Leptothrix cholodnii	171060715	M-TSR-I-AF-Q---E-C		G---LVA--L-G-LSRR-
	Alicycliphilus denitrificans	299129136	--QSQ-M-A-F-D-A--C		GA--LA---V-G-ISRR-
	Microcoleus chthonoplastes	254416508	----KLV-E-F----T-N		AN--QVA--V-GDIA---
	Trichodesmium erythraeum	113477297	--D-FNV-K-F----A-G		GNS-QVA--V-GDIT-F-
	Arthrospira platensis	284054466	--D-RTV-E-F--T-A-G		ADT-QAA--L-GDIT---
	Desulfomicrobium baculatum	256828112	--AEKEV---F-T-LAT-		TE-------V-G--MRE-
	Lawsonia intracellularis	94987449	--SSRE---FF-K---L-		PK----A-L-KILLLHM-
	Desulfatibacillum alkenivorans	218778531	--SAREM------C-EIF		-D--QVC--VTGALA-L-
	Desulfococcus oleovorans	158520030	--GSR-----F--CAAEA		-T--PCA----GDLLGL-
	Thermodesulfovibrio yellowston	206889686	---EK--SEWF-E---LG		GK--EVA------LLRL-
	Natranaerobius thermophilus	188585104	I-A-KS--EFFDEC--T-		DE----A----G-ISRV-
	Ammonifex degensii	260892976	--ATPEV---F-ETL-F-		P--RQVA--V-G-LARC-

Supplemental Figure 10: A partial sequence alignment of Glutamyl-tRNA amidotransferase subunit B showing a one aa insert specific for the *Brachyspiraceae*. Homologs for this sequence were not found in members of the *Sphaerochaeta* in our BLAST search.

Group	Species	Accession	108	Insert	141
Leptospiraceae (13/13)	Leptospira borgpetersenii sero	116330588	RLKKLEAMEADNSFEK	EV	KTKKEVLTLRRELEKL
	Leptospira santarosai str- 200	359685178	----------------	--	-----I----------
	Leptospira interrogans serovar	45656741	------G---------	--	----------------
	Leptospira licerasiae serovar	359689293	------G--T------	--	-----I-S-----D--
	Leptospira biflexa serovar Pat	183221976	---R--Q--EN----Q	-A	R----A-S-K------
	Leptonema illini	488856648	---------ETGT-DQ	-A	R----I-M-Q---D--
	Turneriella parva	392402013	--R---EAF-T-TVHE	I-	-----I-Q-D--IAR-
Other Spirochaetes (0/35)	Brachyspira hyodysenteriae	225619456	----I-KE-V-GT-D-		LP----IL-LK-K-R-
	Brachyspira intermedia	384208873	----I-KE-V-GT-D-		LP----IL-LK-K-R-
	Borrelia burgdorferi	497944348	K-----K--V-GT-DM		IS---ISQ-N--KS--
	Borrelia bissettii	343127445	K-----K--V-GT-DM		IS---ISQ-N--KS--
	Borrelia garinii	51572957	K-----K--V-GT-DM		IS---ISQ-N--KF--
	Spirochaeta smaragdinae	302339355	----I-K--V-GT--S		L----IAK-NK-RSR-
	Spirochaeta africana	383791169	------K--V-GT--S		LS----AK-NK-KAR-
	Sphaerochaeta coccoides	330836657	---I-K--V-GT-DS		L----ISL-TK-KT--
	Treponema pallidum	189025832	----I-R--I-GT--H		LS----AS--K-HA--
	Treponema denticola	498381120	----I-R--I-GA--H		LS----AS--K-HA--
	Treponema paraluiscuniculi	338706567	----I-R--I-GA--H		LS----AS--K-HA--
Other Bacteria (0/>250)	Thioalkalimicrobium cyclicum A	334144061	---E--S--Q-GT---		I--R-----T--M---
	Xanthomonas oryzae pv. oryzae	58426194	---E---A-T-GT-D-		LV-H---S----R---
	Pseudoalteromonas sp. BSi20429	359440425	---D---QSQ-GT---		L----T---N--M---
	Pseudoxanthomonas spadix BD-a5	357417898	---E---A-T-GT---		LV-H---G----RD--
	Glaciecola sp. HTCC2999	221133314	---E---QSQ-GT-D-		L------M-Q--MT--
	Stenotrophomonas maltophilia R	194365044	---E---G-T-GT---		LV-H---G----RD--
	Thioalkalivibrio sp. K90mix	289208671	---D--T-D--G---V		LN-R-A--R---KD--
	Rhodanobacter sp. 2APBS1	352086338	---E---A-T-G----		LV-H---AR---RD--
	Pseudoalteromonas sp. BSi20439	359446683	---D---QSQ-GT---		L----A-M-T--M---
	Alteromonadales bacterium TW-7	119471167	---D--IQSQ-GT---		L----T-M-N--M---
	Alkalilimnicola ehrlichii MLHE	114321014	---D---QAE-GT-D-		L--R-A-S-S--M---
	Halomonas boliviensis LC1	359394092	--RD----RE-GT---		L------VAT--Q---
	Glaciecola nitratireducens FR1	348029862	---D--VQST-GT-D-		I------M-K--MT--
	Idiomarina loihiensis L2TR	56459953	---D--T-SQ-GT---		L----A-VNT--M---
	Shewanella denitrificans OS217	91792911	---E--SQSV-GT-D-		L----A-MRT------
	Mitsuokella multacida DSM 2054	260881835	---E-------GT--V		L-----QG--H-M---
	Megamonas hypermegale ART12/1	291533219	---E-----E-GT--V		LS-----A-K--Q---
	Phascolarctobacterium succinat	323141025	--R--------GT-DL		LP----IS-KH-M---
	Veillonella dispar ATCC 17748	238019369	---Q----AE-GT--V		LP----IG--H-M---
	Selenomonas sp. oral taxon 137	313896175	---E-----ENGT--V		L-----QG--H-M---
	Ruminococcus obeum A2-162	295108498	---QI------GT--V		LP----IE--K--A--
	Lachnospiraceae bacterium 6_1_	331083047	---EI------GT-DV		LP----IN-KK-----
	Clostridium lentocellum DSM 54	326791510	---Q--T--Q-GT--V		LP----IE--K-MD--
	Blautia hansenii DSM 20583	260589615	---EI------GT-DV		LP----IN-KK-----
	Bryantella formatexigens DSM 1	255280807	---QI-T----GT-DV		LP----IA--K-K---
	Butyrivibrio crossotus DSM 287	293115676	---EI------GT-DV		LP----IQ-KK-Q---
	Parvimonas micra ATCC 33270	160947372	------L--E-GT-QR		LP----IK-K--A---

Supplemental Figure 11: A partial sequence alignment of 30S Ribosomal protein S2showing a two aa insert specific for the *Leptospiraceae*.

Group	Species	GI	130		168
Leptospiraceae (13/13)	Leptospira biflexa	12657818	FNKMKLLTGAFARLNP		TASMWFHIGANMHQRERVYIETM
	Leptospira interrogans	45657404	----------------		-----------------------
	Leptospira borgpetersenii	116328165	----------------		-------M---------------
	Leptospira licerasiae	359689797	----------------		-------M---------------
	Leptospira santarosai	359686203	----------------		-------M---------------
	Leptospira noguchii	359724085	----------------		-------M---------------
	Turneriella parva	392401819	.........S......		.......M..........F....
	Leptonema illini	488859423	.....M.......M..		S..................VN..
Other Spirochaetes (0/20)	Treponema socranskii	13560140	--G-NM---S-G-WDG		AKT-Q--V---VD-NV----G--
	Treponema vincentii	257456318	--G-NM---R---EGG	ENAV	-G----------D--I----G--
	Treponema pedis	116805592	--G-NM---R---ETG	ENVV	------------D--M----G--
	Treponema primitia	333999967	--G-N----R-G-IVG	ENVV	------------D---Q-F-G--
	Treponema medium	13560154	--G-NM---R---EGG	ENAV	-G----------D--I----G--
	Treponema azotonutricium	333993151	--G-N----R-G--IG	ENVV	-----L------D---Q-F-G--
	Treponema phagedenis	116805588	--G-NM---R---ETG	ENTV	------------D--T-A--G--
	Treponema denticola	13560138	--G-NM---R---ETG	ENVV	-G----------D--M----G--
	Treponema azotonutricium	333994878	--G-NM---R----TG	QNIA	------------D---Q-F-G--
	Treponema brennaborense	332299029	--G-NM---R---QAG	DNTV	-----L------D--SQ-F-G--
	Treponema succinifaciens	328949180	--G-NM---R---PTG	ENSV	-----L------D--TQ---G--
	Treponema maltophilum	15214031	--G-NM---R---ATG	ENTV	-G---L------D--MQ-F-G--
	Treponema lecithinolyticum	13560146	--G-NM---R---ATG	ENTI	-----L------D--MQ-F-G--
	Treponema pectinovorum	13560156	--G-NM---R--QSTG	ENVV	-G----------D--MQ-F-G--
	Treponema amylovorum	13560158	--G-NM---R--MPTG	ENVV	-G----------D--MQI--G--
	Treponema pallidum	15639779	--G-NM---R---QGG	ENTV	------------D--T-A--G--
	Treponema denticola	13560136	--G-NM---R--QETG	ENTV	------------D--T-A--G--
	Treponema medium	116805566	--G-NM---R---ETG	ENTV	------------D--V-A-VG--
	Treponema phagedenis	116805582	--G-NM---R---ETG	ENTV	-G----------D--T-A--G--
	Treponema caldaria	339498655	--G-NM---R---ATG	ENVV	------------D-------G--
	Spirochaeta smaragdinae	302338405	--G-NM---R---ETG	ENSV	-----L------D----IF-G--
	Spirochaeta thermophila	306532480	--G-N----R---ETG	ENVV	------------D-------G--
	Brachyspira murdochii	296125671	---LNM---R---STG	ENTP	-----L------DE-K----G--
	Brachyspira hyodysenteriae	511892	---LNM---R---STG	ENTP	-----L------DE-K----G--
	Brachyspira pilosicoli	300869853	---LNM---R---STG	ENTP	-----L------DE-K----G--

Supplemental Figure 12: A partial sequence alignment of Flagellar filament core protein flaB protein showing a four aa deletion specific for the *Leptospiraceae*. Homologs for this sequence were not found in members of the *Sphaerochaeta* or *Borrelia* in our BLAST search.

Group	Species	ID	80		123
Leptospiraceae (13/13)	Leptospira interrogans serovar	294828153	FEIGSFQATGNKLDMAITGE	M	GFFKIQMPDGSFAFTRDGSFKID
	Leptospira borgpetersenii	116327820	--------------------	-	--------------S--------
	Leptospira weilii	359726120	--------------------	-	--------------S--------
	Leptospira biflexa serovar	183220919	--------------L-L---	-	A---------T-SYS--------
	Leptospira santarosai	359686429	-------S------------	-	--------------S--------
	Leptospira licerasiae	359689089	--------------L---S-	-	-------------------Y---
	Leptonema illini	488856803	--M--L------F-L-V-S-	V	-----L----T-SY---------
	Turneriella parva	392404077	-QM--L-S--H------SSD	V	-----L----T-GYQ-N-D----
Other Spirochaetes (0/32)	Brachyspira pilosicoli	300871597	-DQ--L--------L-LE--		---QVL-----VSY-------V-
	Brachyspira intermedia	343386079	-DQ--L--------L-LE--		---QVL-----VSY---------
	Brachyspira hyodysenteriae	225619854	-DQ--L--------L-LE--		---QVL-----VSY---------
	Brachyspira murdochii	296127366	-DQ--L--------L-LE--		---QVL-----VSY---------
	Spirochaeta smaragdinae	302338304	-TQ-AL-N---VT-L--Q-D		---RVLLI---YGYS--------
	Spirochaeta thermophila	306532153	-TQ-AL-H-D-VS-L--Q--		---RVLLY--TY-Y---------
	Borrelia turicatae	119953549	--Q-NL---NL-T-V--E-D		--Y--LL---TYGY---------
	Borrelia bissettii	343128071	--Q-KM---NLLT-V--E-D		--Y--LL---TY-Y---------
	Borrelia hermsii	187918627	--Q-NL---NLNT-V--E-N		--Y--LL---TYGY---------
	Borrelia recurrentis	203288201	--Q-NL-V-NLNT-V--E-D		--Y-VLLS--TYGY---------
	Borrelia duttonii	203284668	--Q-NL-V-NLNT-V--E-D		--Y-VLLS--TYGY---------
	Borrelia garinii	219684180	--Q-KM-S-NLLT-V--E-D		--Y--L----TY-Y---------
	Borrelia burgdorferi	218249434	--Q-KM-S-NLLT-V--E-D		--Y--LL---TY-Y---------
	Borrelia spielmanii	224534580	--Q-KM-S-NLLT-I--E-D		--Y--LL---TY-Y---------
	Borrelia afzelii	111115606	--Q-KM-S-NLLT-V--E-D		--Y--LL---TY-Y---------
	Treponema denticola	42526518	--Q--L-N--VTS-I--Q--		---RVLQY--TY-Y---------
	Treponema vincentii	257457574	--Q--L-Q--VST-V--A--		---RVLQY--TY-Y---------
	Treponema brennaborense	332298514	--L-N--VDT---VV--		---RV-QY-----Y-------V-
	Treponema azotonutricium	333994524	-TQ-AL-N-D-AY----Q-D		---R---Y---WSY-------V-
	Treponema pallidum	15639944	--Q--L-H--VSG-V--A--		---RVLQY--TY-Y-------V-
Other Bacteria (0/>250)	Nitrosococcus watsoni	300114797	-TQ-GLTQ---S-----Q-R		---QVL-----L-Y----T-Q--
	Legionella pneumophila	52841453	-SQ--L-N-Q-A--V--E-R		---TVL----TQSY-------T-
	Pseudomonas syringae	237799211	-TA--L-T-N-P--L-VN-R		---QVTQ---TV-YS---T-HL-
	Halothiobacillus neapolitanus	261856358	Q-NLVT-N-A--L--N-R		---QVLQ----V-Y------SL-
	Alkalilimnicola ehrlichii	114320059	Q-NI-Q-E-S--I--E-D		---Q-L----EI-YS-----NV-
	Pseudomonas aeruginosa	15596279	-TP--L-T-EQP----VN-R		---QVLL---TVSY------HLN
	Legionella drancourtii	254496889	--Q--I-H-N-AY-V--Q-Q		---T-L----TQ-Y--N-A-S--
	Pantoea ananatis	291617022	Q-NLSQ-N-SK-I--N-Q		---Q------TS-Y------QV-
	Nitrococcus mobilis	88811803	-TQ-NIVQ---A--L--N-R		---E-LQ---TL-Y------Q--
	Photobacterium profundum	90411007	-TQ-NT-T-N-AM--M-E--		---QVLL---NIGY--N-Q-T-N
	Acidithiobacillus caldus	255021356	MTQ-NLTS---A--LG-N-Q		---Q-MQ---TI-Y----T-QLN
	Vibrio angustum	90579911	-TQ-NT-T-N-AM--M-E-D		---QVLL---NIGY--N-Q-T-N
	Pectobacterium atrosepticum	50120641	Q-T-SE---SK-V--K-Q		---QV-L---TT-Y----A-QL-
	Chromohalobacter salexigens	92114096	Q-GL-N-D-SK-L--N-Q		---AV-----TS-Y------Q--
	Saccharophagus degradans	90021853	-TP--L-V--QS--V--D-R		--IPVLQ---TVSY----Q-HL-
	Pseudomonas fluorescens	70729006	-TA--L-T-EQP--L--N-R		---Q-LQ---TT-Y----T-HLN
	Erwinia amylovora	292489131	Q--LTQ--RDY----Q-K		---QV-L---TT-Y------SLN
	Thiobacillus denitrificans	74317650	-TQ-NL-Q---AK-V--Q-N		---QVL----TTSY------MA-
	Rhodoferax ferrireducens	89902479	-AQ-NL-QS-----V--Q-N		---QVT----TTNY------QVS
	Gallionella capsiferriformans	302878278	Q-NL-Q---Q--V--N-S		---QVL-----TGY------QR-
	Sideroxydans lithotrophicus	291613040	Q-NL-Q-S-Q--V--Q-A		---QVL----TT-Y------QT-
	Comamonas testosteroni	264676572	-LQ--L-QSS-A--V--N-N		---EVN----TI-Y------EV-
	Verminephrobacter eiseniae	121607559	-VQ--L-ESR-N--V--N-N		---QVML----LGY------QV-
	Delftia acidovorans	160896077	-LQ-AL-QSN-A--V--D-N		---QVN----TT-Y------QV-
	Nitrosospira multiformis	82702455	-TQ-NL-Q---SK-V--Q-Q		---QVLR----T-Y------QT-
	Burkholderia glumae	238028847	YTQ-NL-Q---SR-V--N-Q		---QVL----TT-Y------QAN
	Methylobacillus flagellatus	91776311	Q-NL-Q---NK-V--S--		---QVLL----T-Y----A-QL-
	Cupriavidus metallidurans	94312671	Q-NP-Q---AK-V--I-N		---QVT----TTSY------QV-

Supplemental Figure 13: A partial sequence alignment of Flagellar basal-body rod protein flgG showing a one aa insert specific for the *Leptospiraceae*. Homologs for this sequence were not found in members of the *Sphaerochaeta* in our BLAST search.

Group	Species	ID	183		221
Treponema, Spirochaeta, and *Sphaerochaeta* (18/18)	Treponema pallidum pall Nich	3322886	VADDLVEISCVNGNSLIGRG	V	HKSIGHHMEIRGLGIINI
	Treponema denticola	48374381	----V-----I---T-V---	A	N-I---------------
	Treponema azotonutricium	333993934	----V---H--S--I-M-T-	A	N-I-A------------V
	Treponema vincentii	257457470	-S--V---T-I---------	A	N-M---------------
	Treponema paraluiscuniculi	338706552	--------------------	-	------------------
	Treponema phagedenis	320536053	----------L---------	-	NRI---------------
	Treponema succinifaciens	328949096	----I---R------I--Q-	A	N-L-S-------------
	Treponema primitia	333999608	----V---L-----T---A-	A	N-I---------------
	Treponema brennaborense	332298756	----I--LR-----TVL-Q-	A	N-M-S------------V
	Treponema sp. JC4	384108379	----II--R-----T-L---	A	NTM-S-------------
	Treponema saccharophilum	381179163	----I-NVR----STIL-Q-	-	NAL-S------------V
	Treponema caldaria	339501151	----V---R-M---I-M-S-	A	N-I---------------
	Spirochaeta thermophila	307719865	----A---R--A--Y-L-TS	A	NRLL-------------V
	Spirochaeta smaragdinae	302340520	IS--M---R-M---I-M-A-	R	NPVLA------------V
	Spirochaeta africana	373485299	----A--VRN-S--V-L-S-	A	N-AL--------------
	Spirochaeta thermophila	307719865	----A---R--A--Y-L-TS	A	NRLL-------------V
	Sphaerochaeta coccoides	330837531	IS--T-KLRNIGD-H---S-	E	NPLLA------------V
	Sphaerochaeta globosa	325970610	IS--T-KLRNISD-Y---M-	E	NPLLA------------L
	Sphaerochaeta pleomorpha	359351273	IS--T-RLRNISD-Y---M-	E	NPLLA------------L
Other Spirochaetes (0/18)	Brachyspira murdochii	296125291	IS--T--FRKLRDGRI--KT		NEF-K-N--V--I-VVD-
	Brachyspira pilosicoli	300871210	I---T--FKKLKDGRI--KK		-DV-K-K--V--I--VD-
	Brachyspira intermedia	343387028	IS--T--FKKLKDGRI--RK		NEY-K-N--V--I-VVD-
	Brachyspira hyodysenteriae	225619048	IS--I--FKKLKDGRI---K		NEY-K-N--V--I-VVD-
	Leptonema illini	373874416	----M--LR-LADSY-M-YT		SSI-E-N--L--I---DV
	Leptospira santarosai	359686188	----M---RRLSESY---TC		SDLLR----------L--
	Leptospira noguchii	359724074	----M---RRLSESY---TC		SDLLR----------L--
	Leptospira weilii	359726698	----M---RRLSESY---TC		SDLLR----------L--
	Leptospira licerasiae	359689773	----M---RRLSESY---TC		SDLLR----------L--
	Leptospira borgpetersenii	116331058	----M---RRLSESY---TC		SDLLR----------L--
	Leptospira interrogans	24215103	----M---RRLSESY---TC		SDLLR----------L--
	Turneriella parva	392401991	----A-R-I-RD-SR-Y-YV		SQT-E--L--------S-
Other Bacteria (0/>250)	Staphylococcus aureus	312438812	----N---RQI-KDE---KP		P-L-E-LL---------V
	Leptospira biflexa	183221038	----M---RRLSESY---SC		SDLLR----------L--
	Pelobacter propionicus	118579412	----M-Y-KKKMPAT-V-QS		E---Q-L-----------
	Plesiocystis pacifica	149919230	----V--VTIRPPDTIW-SA		THLHQ-------M--L--
	Myxococcus xanthus	108757669	----I-DVTRRK- AVY-A-		NPV-K-------------
	Chthoniobacter flavus	196232253	-S--MTRFRALE-RE-V-TS		PDLTRN--------V--V
	Clostridiales genomosp.	289450449	I------VRR-SDTT-L--A		PQMLR-LL-L------DV
	Anaerococcus prevotii	257066536	I---M-D-IS-- -R-S-TS		PEN-R-Y----------V
	Ruminococcus flavefaciens	268610015	----A---RK-SNI--V-SS		PDN-R-FL-L--I----
	Anaerococcus lactolyticus	227485076	----M-D-TAID KKIV-EA		PEN-R-F--L-------V
	Acetivibrio cellulolyticus	303241087	----V---RR-SDK--V-TA		PDI-R-FI----I--LDV
	Ethanoligenens harbinense	289639254	I---A---RR-SSKT-V-TS		PEN-R-FV-L--I--V-
	Eubacterium siraeum	291531397	----A---RKASNIT-V-SS		PDN-R-F--L--I----
	Mariprofundus ferrooxydans	114778516	I---M--F-RKSPGVVV--S		PEALRY---------L--
	Candidatus Protochlamydia	46446108	IS--I-KVKKKE-TY-E-S-		VALTR-------I----V
	Waddlia chondrophila	297621646	IS--I-KVKKRE-HY-E-S-		AELTR-------I----V
	Elusimicrobium minutum	187250545	-S--I--VQRRR--I---SC		PNM-R-Y--V------DV

Supplemental Figure 14: A partial sequence alignment of conserved region of Bifunctional Hpr kinase/phosphatase showing a one amino acid insertion specific for *Treponema*, *Spirochaeta*, and *Sphaerochaeta*. Homologs for this sequence were not found in members of the *Borrelia* in our BLAST search.

Group	Species	GI	1		39
Treponema, Spirochaeta, and *Sphaerochaeta* (18/18)	Spirochaeta smaragdinae	302337499	MARIAGIDLP		NKATKISLTYIYGIGRSSAMEICKKAGID
	Spirochaeta africana	373484190	----------		--PVR-A---------T--EK--E-T-V-
	Spirochaeta coccoides	330837633	------V---		---V--A--------NY--AK--EQTAVN
	Spirochaeta thermophila	386346288	------V---		--QIQ-A-----------RK--ERT--P
	Sphaerochaeta globosa	325972639	------V---		---V--A---------F--V---E-TN-N
	Sphaerochaeta pleomorpha	359352158	------V---		---V--A---------V--VD--E-TK-N
	Treponema caldaria	339499044	------V---		--HVN-A---------P--D---A-TK--
	Treponema paraluiscuniculi	338706185	----------		--HVSVAL------S----RT--E--R-S
	Treponema denticola	42526301	------V---		--HVNV--------ST---NK--EATKV-
	Treponema vincentii	257456914	----S-V---		--HVN-A-------SH---KA--E-SK--
	Treponema pallidum	15639203	------V---		--HVSVA-------S----RT--E--R-S
	Treponema succinifaciens	328949255	------V---		--HVN-A---V--------NK--EATNV-
	Treponema phagedenis	320538144	------V---		--HVN-A-------SH---KT--E--K--
	Treponema primitia	333998869	------V---		--HVN-A---V--------D---T--K--
	Treponema azotonutricium	333996144	---LV-V---		--HVN-A---V--------D---V--K--
	Treponema brennaborense	332298891	------V---		--HVN-A-----------D-----TK-N
	Treponema azotonutricium	333996144	---LV-V---		--HVN-A---V--------D---V--K--
	Treponema saccharophilum	381181180	------V---		--HVV-A-----------KL--E-TK--
	Treponema sp. JC4	384108323	------V-I-		--HLGTA----------A-AK--EE-KL-
Other Spirochaetes (0/30)	Borrelia sp. SV1	225552343	----S-----	N	--QL--A--S------TR-L-V-N-SS-S
	Borrelia hermsii	187918366	----------	N	--QLQ-A--S------AR-L---E-T-VL
	Borrelia duttonii	203284410	----------	N	--QLQ-A--S-------R-L-----T--L
	Borrelia turicatae	119953289	----------	N	--QLQ-A--S------AR-L---R-TD-L
	Borrelia afzelii	216263668	----S-----	S	--QL--A--S------TR-L-V-N--D-S
	Borrelia garinii	51598755	----S-----	S	--QL--A--S------TR-L-V-N-SS-S
	Borrelia burgdorferi	221218102	----S-----	N	--QL--A--S------TR-L-V-N-SS-S
	Borrelia spielmanii	224534712	----S-----	S	--QLR-A--S------TR-L-V-NR-S-S
	Borrelia valaisiana	224531523	----S-----	N	--QL--A--S------TR-L-V-NRSN-S
	Brachyspira murdochii	296126888	---LM-VEIR	N	--RIE-A--D------TL-HV--D--N--
	Brachyspira pilosicoli	300870625	---LM-VEIR	N	--RIE-A--D------TL-HV--D--N--
	Turneriella parva	392404107	----E-----	N	Q-RVV-G--------DKV-HD-VT----P
	Leptonema illini	488862859	----S-V---	K	D-RIV-G--------DTR-R--L-A---
	Leptospira biflexa	183221325	------V---	S	--RIV-G----F---KT-SQN-L------
	Leptospira vanthielii	489072990	------V---	S	--RIV-G---VF---KT-SQS-L------
	Leptospira weilii	488704326	----------	R	E-RIV-G----F---N-LSRV-L------
	Leptospira licerasiae	495870971	----------	R	E-RIVVG----------TSRKLLA---V-
Other Bacteria (0/>250)	Ammonifex degensii	260893347	------V---	K	D-RVE-A-----------KK-LE-T-VN
	Eubacterium siraeum	167749413	----------	K	E-RVE-G---V-----KT-ND-LA---VN
	Dorea longicatena	153854995	------V---	R	D-RVE-G---------T--TR-LTE--VN
	Parvimonas micra	160947675	----V-V---	R	E-RVE-G---------KTSND-LRN---N
	Oribacterium sinus	227871777	------V---	R	E-RIE-G---------P--DK-LAET-VN
	Pseudomonas putida	170723884	------VNI-	D	--H-V--------V--TT-QK--AD--VN
	Olsenella uli	302336356	----N-V---	R	E-RVEVG---L----QT--TKV-AET-VN
	Lactobacillus gasseri	300362366	------V---	R	--RVVVA--------E-T-KK---D---S
	Chitinophaga pinensis	256420683	----------	K	--RGE-G----F-----T-QY-LE-S---
	Thermobaculum terrenum	269925856	------V---	R	D-RVEVA--------PT-SKK-LE-T-VN

Supplemental Figure 15: Partial sequence alignment of conserved region of 30S ribosomal protein S13 showing a one amino acid deletion that is common to *Treponema, Spirochaeta,* and *Sphaerochaeta*.

Group	Species	GI	103		136
Borrelia (12/12)	Borrelia spielmanii	224534698	KVKRLAFKSVLSLRAADESS	F	KVVENFNIESGKT
	Borrelia garinii	386853883	------------------NN	-	-------------
	Borrelia valaisiana	224532312	------------------NN	-	--I----------
	Borrelia bissettii	343127786	---K--------------N-	-	--I----------
	Borrelia burgdorferi	15594824	---K--------------N-	-	--I----V-----
	Borrelia afzelii	111115308	---------------S--NN	-	--I----------
	Borrelia hermsii	187918345	-------R-----C-SA-DR	L	-I----T------
	Borrelia duttonii	203284389	-------------C-SVDDR	L	------T-D----
	Borrelia crocidurae	386859711	-------------C-SVDDR	L	------T-D----
	Borrelia turicatae	119953268	-------R-----CVSV-DR	L	-I----T------
Other Spirochaetes (0/36)	Treponema pallidum	378972695	------M--L---K-QGDAL		T-I-D-TV-----
	Treponema paraluiscuniculi	338706164	------M--L---K-QGDAL		T-I-D-TV-----
	Treponema succinifaciens	328949276	-E----M--I---Q-QADRL		T---D-T------
	Treponema primitia	333998888	-A----L-TI---K-QSDTL		----D-S-D----
	Treponema denticola	42526280	-A----M--I---K-QNDRL		V---D-TV-----
	Treponema brennaborense	332298912	------M--I---K-QGDRL		T-I-D-TV-----
	Treponema phagedenis	320538164	-A----M--L--MK-Q-DRL		T---D-T------
	Treponema vincentii	257456893	-A----M--I---K-Q-DRL		V-I-D-TV-T---
	Treponema saccharophilum	381181199	TA----M--I---Q-QADRL		T---D-T------
	Treponema azotonutricium	333996164	-A-Q--I-TI---KVQSDML		-I--D-TV-----
	Treponema sp. JC4	384108302	-E----M-TI--SH-QGDRL		TI--D-TV-----
	Treponema caldaria	339499023	-A----L--I---K-QSDIL		----D-TV-----
	Spirochaeta smaragdinae	302337478	-M----M------K-KEDAI		----D-TV-----
	Spirochaeta thermophila	307718207	------YR-----KLRENAF		R---D-VV-----
	Spirochaeta africana	383789789	-L----Y--I--QKVQQ-DI		V---D-AL-----
	Sphaerochaeta globosa	325972660	-M----M--L---GVKE-RL		V---D-S-D----
	Sphaerochaeta pleomorpha	374316493	------M--L---GIQE-RL		V---D-SP-----
	Sphaerochaeta coccoides	330837654	------M--L---SVQE-RL		V---D-TA-----
	Brachyspira pilosicoli	00870646	-M--K-LL-----KYGNNVL		--F-D-TFDAP--
	Brachyspira hyodysenteriae	225621029	-M--K-LL-----KYGSNVL		--F-D-TFDAP--
Other Bacteria (0/>250)	Bacteroides ovatus	160883063	---T--R--A--YK-QNDAI		V---D-TF-AP--
	Prevotella buccalis	282878760	---N--RR-A--YK-QENAI		VI--D-TLDAP--
	Microscilla marina	124003663	---S--R--A-TYK-KENNI		T---D--L-AP--
	Psychroflexus torquis	91216909	-L----R-T---QKLKNNQL		MI--D-GF-AP--
	Bacteroides plebeius	198274002	---V--R--A-AYK-QTNNI		V---D-TF-AP--
	Dokdonia donghaensis	86132545	GL----RR-A-TMK-N-NAI		T-I-D--FDAP--
	Gramella forsetii	120437173	NL----R--A--IK-N-KAI		M-I-D-SFDTP--
	Prevotella tannerae	258648158	-L-A--RR-A--YK-KEN-I		V-L-D-TM-TP--
	Kordia algicida	163752916	N-----R--A-T-K-KS--I		I-I-D-DFDAP--
	Rhodothermus marinus	268316409	-TQ---RR-A-TYK-QA-AI		R---D-TF-QPS-
	Croceibacter atlanticus	298207728	-Q-Q--RR-A--QK-Q-NAI		VI----SFDAP--
	Persephonella marina	225850736	--RKK-L-G---MKLR-GEL		T-I-D-TFDEP—
	Eubacterium siraeum	291532094	------L--A--AKVQ-G-M		I--DSITADEF--
	Veillonella dispar	238018624	-AR---V--A--DKVNNSEL		Y-L-EITLAAP--
	Haliangium ochraceum	262197187	--RKK-LR-A-----KEQKL		V-LDA-PV-G---
	Staphylococcus aureus	15925239	-MR---LR-A--FK-QENGL		T--DA--F-AP--
	Pediococcus pentosaceus	116493162	-MR---L-----QKVL---L		V--DE-KF-TP--
	Listeria monocytogenes	47094373	--R---I--I--SKVNE-KL		V-L-GLTFDAP--
	Aerococcus viridans	295398289	--R---LR-A--TKV-ENNF		I--DELAF-TP--

Supplemental Figure 16: A partial sequence alignment of conserved region of 50S ribosomal protein L4 showing a one amino acid insertion specific for the genus *Borrelia*. Homologs for this sequence were not found in members of the *Leptospiraceae* in our BLAST search.

			143		178
Borrelia (12/12)	Borrelia duttonii	203284699	INIYDIEILDYNIDF	RI	LKLKIKCSKGTYVRSIARD
	Borrelia crocidurae	386860042	---------------	--	-------------------
	Borrelia recurrentis	203288231	---------------	C-	-------------------
	Borrelia turicatae	119953580	V-----Q--N--V-S	--	---Q---------------
	Borrelia hermsii	187918657	V-----Q--N--V-S	--	---Q---------------
	Borrelia valaisiana	224532106	V-V---Q--N-DFNS	SL	-S---S------I------
	Borrelia spielmanii	224534135	V-V-N-Q--S-DFNS	SL	-S---S------I------
	Borrelia burgdorferi	226320850	VTV-N-QR-S-DFSS	FL	-S---S------I------
	Borrelia bissettii	343128100	VTV-S-QR-N-DFSS	SV	-S----------I------
	Borrelia garinii	219685675	V-V---QR-S-DFSS	SS	-S---T------I------
Other Spirochaetes (0/36)	Treponema saccharophilum	381179451	-T--S-K-V-FMDKY		AL-EVR------I--L---
	Treponema azotonutricium	333994686	VTV-E--LSSWDPPL		AG--VR--A---I--L---
	Treponema caldaria	339499975	-R-HEL-V-H-EEPR		-T-RVH------I-AL---
	Spirochaeta smaragdinae	302338540	VT-HNL--TAFDGEK		AVIRVT------I--L---
	Spirochaeta thermophila	307718802	VE--ALSL-EWRPPY		ATVRVS--G---I-AL---
	Sphaerochaeta coccoides	330836565	VT--SF-PVSFDGCN		-VADVHV-----I--L---
	Brachyspira hyodysenteriae	225619559	VC-EK--L-E-DYPY		F-I-TSV-----I---I--
	Brachyspira intermedia	384209155	VC-EK--L-E-DYPY		F-I-TSV-----I---I-
	Leptospira interrogans	488071099	-K--RY-LGEFSPES		ISFQ-RV-A---I-K-VM-
	Leptospira inadai	498103995	-K--EF-VRNFEESG		-TIRT-V-G---I-KLVM-
	Leptospira broomii	498255112	-K--EF-VRNFEETG		-TIRT-V-G---I-KLVM-
Other Bacteria (0/>250)	Hahella chejuensis	83644103	-I-HEL-L---TPP-		-R-RVL------I-NLVE-
	Alteromonas macleodii	239997159	-EV-ELDV-RIELP-		VEMR--------I---VD-
	Haemophilus somnus	113460966	-S-FELKFI--Q-PY		-T-EVH------I-TLVD-
	Alcanivorax borkumensis	110833192	VT---LR--RIDG-E		-EFEVD---------LVE-
	Haemophilus influenzae	260581411	-T-FELNFIE--AP-		-T-EVH------I-TLVD-
	Pasteurella multocida	15602621	-T-F-LQFIA-DAPY		-T-EVH------I-TLVD-
	Pasteurella dagmatis	260914251	-M-FELNFIE--APY		-T-D-H------I-TLVD-
	Xanthomonas fuscans	294665175	VDVQA--V-G-GAPR		-R-RVT-GS---I--L---
	Sodalis glossinidius	85058357	-HV--LQLHRWDLTK		VE-E-H------I-T-ID-
	Dichelobacter nodosus	146329574	-H-ASCSVIAV-K-Y		AQ-R-R----S-I-AF---
	Vibrio splendidus	218710443	-TV-S--L-RFEG-E		VEMEVH------I-T-TD-
	Anaerococcus vaginalis	256544701	-KV--F----FDFPY		ARF--T------I--LVN-
	Clostridium botulinum	253682681	VT--------IS-PY		I-FMV-------I--LCY-
	Anaerococcus tetradius	227499291	V---------FAFPQ		ARILV-------I-TLID-
	Oribacterium sinus	227872008	-E-FAL---S-DCPK		-RFR-H------I-TLCK-
	Anaerococcus prevotii	257066119	VK---LKLI-FDFPY		A-ISVT------I-TLVD-
	Halothermothrix orenii	220931631	VE-KK-N---IDLPK		V--FVL--P---I------
	Dorea formicigenerans	166030870	VQ-F-----SIALPR		VTM-VH------I-TLCH-
	Coprococcus comes	226323224	-T-HE-R--EI-LPE		V--EVT------I-TLCH-
	Veillonella parvula	282848833	-H-KN--LIA-GFP-		FTVRVT--G---I--LL--
	Alistipes putredinis	167752309	----EM-LME-DLPR		IRIRVR------I--L-
	Psychroflexus torquis	91217024	V--DSFD-IQN-FPK		-NF--Q------I--L-Y-
	Planctomyces maris	149174741	VDV-E--L--FSFPR		FQ-R-V-GS---I---G--
	Methylotenera mobilis	253995586	V--HN--V-SFAG-V		AEITVT------I-TL-E-
	Eikenella corrodens	225024511	-H-H--Q--RFQFPE		AQISVQ------I-TL-E-
	Simonsiella muelleri	294788179	-T--Q-D-IEF-FPK		AVIDVR------I-TLSE-
	Dechloromonas aromatica	71908068	VT-FA-DC--FSG-L		-T-RVA------I-VL-A-
	Neisseria flavescens	225075531	-T--S-D-TEF-APK		AVIDVR------I-TLSE-

Supplemental Figure 17: Partial sequence alignment of conserve region of tRNA pseudouridine 55 synthase showing a two amino acid insertion specific for the genus *Borrelia*.

Group	Organism	Accession	330		369
Borrelia (12/12)	Borrelia duttonii	203284386	PQFFFRTTDVTGMVSLE		GKEMVMPGDNVDIVVELISSIAM
	Borrelia crocidurae	386859708	-----------------		-----------------------
	Borrelia bissettii	343127783	------------V-A--		-------------I---------
	Borrelia turicatae	119953265	-----------------		-----------------------
	Borrelia lonestari	145652262	--------------N--		-----------------------
	Borrelia hermsii	187918342	-----------------		-------------------L---
	Borrelia afzelii	111115305	------------V-A--		-----------------------
	Borrelia spielmanii	224534799	------------V-A--		-----------------------
	Borrelia garinii	219684436	------------V-A--		-----------------------
	Borrelia valaisiana	224532207	------------V-A--		-----------------------
	Borrelia burgdorferi	2688415	------------V-A--		-------------I---------
Other Spirochetes (0/36)	Treponema primitia	333998891	---Y-----I--TI--P	E	-VD--K----TK-IG---HP---
	Treponema pallidum	161579586	---Y-----I--TI--P	E	-VD--K----TK-IG---HP---
	Treponema caldaria	339499020	---Y-----I--T-K-P	E	----------TE-FG---HP---
	Spirochaeta thermophila	386346264	---Y--------S-Y-P	D	D-Q-------AE-T----TPV--
	Spirochaeta smaragdinae	302337475	---Y-----I--T-N-P	A	D-Q-------AELEI---HP---
	Sphaerochaeta coccoides	330837657	---Y-----I--T-L--	E	GK--L---HTELE----HPV--
	Brachyspira intermedia	384210014	--MY--------VIN-A	E	-AQ-I-----ANLTI---TP---
	Brachyspira murdochii	296126863	--MY--------VIN-Q	E	-SQ-I-----ANLTI---TP---
	Turneriella parva	392404081	---Y--------T-N-P	T	-V---N-----T-TA----P---
	Leptonema illini	488862824	---Y--------TIM-P	A	---V-I-----TVD----TP---
	Leptospira interrogans	24213437	---Y--------VCN-P	N	-V---------SLT-----P---
	Leptospira borgpetersenii	116327222	---Y--------VCN-P	N	-V---------SLT-----P---
Other Bacteria (0/>250)	Rhodospirillum centenum	209964023	---Y----------T-P	E	-T---------R-R----AP---
	Acidiphilium cryptum	148260941	---Y--------V-T-P	E	-V---------TVS----AP---
	Gluconobacter oxydans	58038857	---Y--------V-T-P	E	-T---------AMD----AP---
	Agrobacterium vitis	222148347	---Y--------I---P	E	-T---------TVQ----VP---
	Hyphomonas neptunium	114799205	---Y--------I-K-P	E	D----L-----KMD----NP---
	Rhizobium leguminosarum	209548918	---Y--------I-T-P	E	-T---------TVA----VP---
	Stappia aggregata	118591186	---Y--------V---P	E	-T---------SVE----VP---
	Roseomonas cervicalis	296537446	---Y--------V-Q-P	E	-V---------AMD----AP---
	Methylocella silvestris	217976769	---Y--------V-T-P	E	-T---------TMD----AP---
	Oceanicaulis alexandrii	83858568	---Y--------V-I-K	E	-T---------EVS----QP---
	Rhodospirillum rubrum	83594034	---Y--------TIE-P	E	-T--------IGMT-Q--AP---
	Moraxella catarrhalis	296112456	---Y--------AIT-Q	E	-T---------EMS----HP---
	Vibrio vulnificus	27364738	---Y--------DI--P	E	-V--------IQM------P---
	Proteus mirabilis	197287064	---Y--------TIE-P	E	-V---------NMI----HP---
	Enhydrobacter aerosaccus	257455971	---Y--------AIQ-P	E	-T---------EMN----HP---
	Psychrobacter arcticus	71064958	---Y--------AIQ-Q	D	-T---------EMG----HP---
	Grimontia hollisae	262273383	---Y--------TIE-P	E	-V--------IKMI----AP---
	Aliivibrio salmonicida	209693919	---Y--------DIT-P	E	-V---------QMT----AP---
	Yersinia pestis	22124392	---Y--------TIE-P	E	-V---------NM--N--AP---
	Xenorhabdus bovienii	290477024	---Y--------TIE-P	E	-V--------INMI-T--AP---
	Lutiella nitroferrum	224827264	---Y--------A---A	E	-V---------E-T----AP---
	Comamonas testosteroni	264676487	---Y--------SIE-P	E	-----------S-T-K---P---
	Ralstonia pickettii	187930366	---Y--------SIA-P	E	-----------S-T-K--AP---
	Neisseria gonorrhoeae	293398222	---Y--------A-T--	K	-V------E--T-T----AP---
	Kingella oralis	238023037	---Y--------A-T-S	E	-V------E--T-T----AP---
	Simonsiella muelleri	294789160	---Y--------A-T-S	E	-V------E--K-T----AP---
	Taxeobacter ocellatus	1169497	---Y--------II--A	E	-V---------T-S----NAV--
	Pedobacter heparinus	255530754	---Y--------EI--A	E	-T---------T-T-K--NA---
	Bacteroides capillosus	154499816	---Y--------IIT-P	E	-T--C-------MD----TP--I
	Kribbella flavida	284034004	---Y--------V-T-P	E	-T--------T-MS----QP---
	Salinispora arenicola	159039836	---Y--------V-T-P	E	-T--------TTMT-K--QP---
	Solibacter usitatus	116624204	---Y--------VAQ-P	E	-T---------SLE----TPV--
	Eubacterium dolichum	160914563	---Y--------VIT-P	E	-T---------EMT----AP--I
	Clostridium botulinum	148381423	---Y--------SIN-P	E	-V-------HI-MA----TPV--

Supplemental Figure 18: A partial sequence alignment of conserved region of Translation elongation factor TUshowing a one amino acid deletion specific for the genus *Borrelia*.

Group	Species	ID	273		301
Borrelia (12/12)	Borrelia hermsii	187918014	RGLDYYTGLVFEAEMMGI		NMGSICSGGRY
	Borrelia duttonii	203284061	---------------L-V		-----------
	Borrelia recurrentis	203287600	---------------L-V		-----------
	Borrelia crocidurae	386859366	---------------L-V		-----------
	Borrelia turicatae	119952938	--------I------I--		-----------
	Borrelia garinii	219685912	--------I---S-IF-S		----V------
	Borrelia burgdorferi	195942375	--------I---S-VF-S		----V------
	Borrelia valaisiana	224531959	--------I---S-VF-S		----V------
	Borrelia bissettii	343127457	--------I---S-VF-S		----V------
	Borrelia spielmanii	224534329	--------I---S-VF-S		----V------
	Borrelia sp. SV1	225551966	--------I---S-VF-S		----V------
	Borrelia afzelii	111114958	--------I---S-VFDS		----V------
Other Spirochaetes (0/36)	Treponema saccharophilum	381180018	--------V-Y-TFLN-C	P	EI--V------
	Treponema denticola	42526950	--------V---TFLNDL	P	SI--V------
	Treponema phagedenis	320536997	--------V---TFLTEL	P	EI--V------
	Treponema azotonutricium	333994759	--------I-Y-TLLKEM	P	EL--V------
	Treponema succinifaciens	328948006	--------I-Y-TFLEKL	P	SI--V------
	Treponema brennaborense	332297974	--------V-Y-TFLNAL	P	SI--V------
	Treponema vincentii	257456490	--------I-Y-TFLTDL	P	QL--V------
	Treponema caldaria	339499953	--------V-Y-TFLNDL	P	EI--V------
	Spirochaeta thermophila	386347361	--------V---TFLSDL	P	DI--V------
	Sphaerochaeta pleomorpha	374314911	--------I-Y-TFLTDL	P	-F--V------
	Sphaerochaeta globosa	325971158	--------I-Y-TFLTDL	P	-F--V------
	Brachyspira pilosicoli	404475790	-------KTA--VQTNAL	G	SQSA-LG----
	Brachyspira murdochii	296125535	-------KTA--VQTNAL	G	AQSA-LG----
	Turneriella parva	392401903	--F-----M---VFDTHP	E	-RR-LFG----
	Leptonema illini	488863069	--F---------IYDTNP	E	-RRA-FG----
	Leptospira santarosai	490626428	--F-----CI--VFDTNS	E	-KR-LYG----
	Leptospira wolbachii	505588704	--F-----FI--IFDTSP	Q	-KR-LYG----
	Leptospira yanagawae	505584542	--F-----FI--IFDTSP	Q	-KR-LYG----
Other Bacteria (0/>250)	Planctomyces maris	149177824	--------TIY-TFLNQL	P	GI--V------
	Gemmata obscuriglobus	168702989	--------TIY-TFLTDL	P	GI--V------
	Finegoldia magna	302380644	--------S---TFFKDY	P	EI---------
	Parvimonas micra	160946147	--------T-Y-TFLENY	R	QL--V------
	Xylella fastidiosa	28199156	--------T-Y-TALINH	P	QI---------
	Wolinella succinogenes	34557793	---G----I-Y-TTLDAL	P	SI--V------
	Mobiluncus curtisii	298346863	--------M-Y-TFIT-A	E	SY---------
	Kytococcus sedentarius	256824814	--------T-Y-TFLDEH	P	EL---------
	Scardovia inopinata	294790955	--------S-Y-TFLD-A	E	SL--V------
	Bifidobacterium longum	312132333	--------S-Y-TFLD-A	A	SL---------
	Micrococcus luteus	289706220	--------T-Y-TVLV-H	E	QL---------
	Kocuria rhizophila	184201003	--------T-Y-TVLV-H	E	KL--V------
	Rothia dentocariosa	300741588	--------T-Y-TVLV-H	E	SL---------
	Beutenbergia cavernae	229820494	--------S-Y-TVLV-H	E	DL---------
	Sanguibacter keddieii	269795067	--------S-Y-TVLV-H	E	EL---------
	Bacteroides capillosus	154496106	--------T-Y-TV-LDH	P	EV---------
	Lentisphaera araneosa	149199644	--------S-Y-CVIN-L	E	SY--V------
	Nodularia spumigena	119512876	--------T-Y-TTLL-H	E	AL---------
	Nostoc punctiforme	186685474	--------T-Y-TTLL-H	E	AL---------
	Raphidiopsis brookii	282895590	---N----T-Y-TTLI-H	E	SL---------
	Anabaena variabilis	75908477	---N----T-Y-TTLI-H	E	AL---------

Supplemental Figure 19: Partial sequence alignment of conserved region of Histidyl-tRNA synthetase showing a one amino acid deletion specific for the genus *Borrelia*.

Group	Species	Accession	231		264
Borrelia (12/12)	Borrelia hermsii	187918098	EITLGGYYYNTILDLKS		PLRMAGLSHCFRKEAG
	Borrelia crocidurae	386859451	-----------------		--K-------------
	Borrelia turicatae	119953022	-----------------		--K-------------
	Borrelia duttonii	203284145	-----------------		--K-------------
	Borrelia recurrentis	203287683	-----------------		--K-------------
	Borrelia burgdorferi	226321201	----------K-I--TL		--R---F---------
	Borrelia afzelii	216263748	----------K-I--TL		-IK---F---------
	Borrelia spielmanii	224534318	----------K-I--T-		-IK---F---------
	Borrelia valaisiana	224531827	----------K-I--LT		-IK---F---------
	Borrelia bissettii	343127544	----------K-I--TL		-IR---F---------
	Borrelia sp. SV1	225552155	----------K-I--TL		-IR---F---------
	Borrelia garinii	386853634	----------K-I--TL		-I----F---------
Other Spirochaetes (0/36)	Treponema primitia	374815548	--------S----SREK	L	--R---------R---
	Treponema azotonutricium	333995154	--------S-M--PKEK	L	--R---------R---
	Treponema sp. JC4	384108580	-------HKDE---KA-	L	--LYG-------R---
	Treponema succinifaciens	328948174	-------HSGE---KSK	L	--MYC-------R---
	Treponema brennaborense	332297813	-------HSGE---KSK	L	--LYC-------R---
	Treponema phagedenis	320535443	-------HADE--SQTK	L	--LYC-------R---
	Treponema saccharophilum	381181448	------FHSGE--AKEA	L	--YYC-------R---
	Treponema paraluiscuniculi	338706601	-----A-HAGEV-EER-	L	-RLY------------
	Treponema pallidum	15639634	-----A-HAGEV-EER-	L	PRLY------------
	Treponema vincentii	257457271	-------HSDE---KAK	-	--MYC-------R---
	Treponema denticola	42527809	-------HSDE-IKKE-	-	--KYC-------R---
	Treponema caldaria	339499194	--------SG---SKDK	L	--R---------R---
	Spirochaeta africana	383790986	-------HAGKM-TASE	L	-IRL--------R---
	Spirochaeta thermophila	386347714	------M-ADE-IPGQA	L	--RF------------
	Spirochaeta smaragdinae	302339395	-------HAGELIDGQR	-	-IKL--------R---
	Sphaerochaeta pleomorpha	374317524	--------A-Q---QSQ	L	-I--T-------R---
	Sphaerochaeta globosa	325971623	--------ADQ---REQ	L	-I----------R---
	Sphaerochaeta coccoides	330836880	--------SGE---KAN	L	---L--------R---
	Brachyspira pilosicoli	300870425	-VP-TNI-REE-IPENM	L	--YCTAYTP---S---
	Brachyspira murdochii	296126531	-VP-TNI-REE-IPENM	L	-IYATAYTP---S---
	Turneriella parva	392402053	-VP-TNL-ADE--NAAE	L	-IKLTAFTP---R---
	Leptonema illini	488863018	-VP-VNL--DELIKEEE	L	-VAVTAA-S---R---
	Leptospira wolbachii	505589699	-VP-TN--RDE-ISE-E	L	-ISVCAHTP---R---
	Leptospira meyeri	463323142	-VP-TN--RDE-ISE-E	L	-ISVCAHTS---R---
Other Bacteria (0/>250)	Blastopirellula marina	87310730	------MNANKV-EAED	-	-KLYC-M---Y-T---
	Gemmata obscuriglobus	168700966	------MHRDR-F-EAE	-	-K-YV-------T---
	Pirellula staleyi	283780181	------M-ADQTV--EQ	-	-IKL--I-----T---
	Planctomyces maris	149179060	------SMKDQ-M-RET	-	-YKI--------T---
	Plesiocystis pacifica	149919214	------MVADE---PEQ	-	--LV--------T---
	Campylobacter hominis	154149298	-V-ATNF-NGE--SEDE	-	-VKFTSY---------
	Desulfo. retbaense	258405089	-VP-TNLHAGEV--EQD	-	-RGY-AFTP---S---
	Conexibacter woesei	284042611	-VA-ASLHQGE--AEEE	-	-R-Y--F-P---R---
	Rhodococcus equi	296038806	-VP-A--HSGE----SK	G	-K-Y--W-S---R---
	Nocardia farcinica	54022104	-VP-A--HADE----SA	G	-K-Y--W-S---R---
	Thermobifida fusca	72160435	-VP-A--HANE--PADA	-	-T-YI-W-S---R---
	Eggerthella lenta	257792717	-VQ-TNIHAGE---AGQ	-	--KYCAFTP---E---
	Ferroglobus placidus	288930586	-HP-AAMHM-ET-GEDE	-	--LY--V-P-------
	Naegleria gruberi	291002089	--G-AALHC-EFMPNFK	G	--KY--------T---
	Veillonella dispar	238019657	-V--TN-HSGE--SEEE	-	-KYYTAFTA---A---
	Bacillus tusciae	295695170	-VP-VS--ADEV-SEEE	-	-K-ML-I-N---R-V-
	Theileria annulata	85000409	-QPIAALHRGEVYQK-Q	-	-I-Y--I-T---R---
	Plasmodium chabaudi	70941484	-QP-CALHRDET-ESEY	-	--KYV-I-S-------
	Trypanosoma cruzi	71421977	-MPIAA-HRGKWFTELK	E	--KY--M-S-------

Supplemental Figure 20: A partial sequence alignment of conserved region of Seryl-tRNA synthetase showing a one amino acid deletion specific for the genus *Borrelia*.

Group	Species	ID	114		154
Borrelia (12/12)	Borrelia garinii	219684344	LIGREGKNLDSLQLLTNVYTSK	LIG	ENGSFNRVVLDIGDYR
	Borrelia burgdorferi	15594788	-------------------A--	---	---A----I-------
	Borrelia afzelii	111115270	---------------------R	---	-T-A----I-------
	Borrelia valaisiana	224532080	-------------------A--	---	-T-A----I-------
	Borrelia spielmanii	224534746	----------------------	---	-TST----I-------
	Borrelia hermsii	187918309	------R---A----A---V-R	---	D--N---II---E---
	Borrelia turicatae	119953232	------R---A----A---M-R	---	D--I---I----E---
	Borrelia duttonii	203284352	------R---A----A-I-I-R	---	DT-N----I---E---
	Borrelia sp. SV1	225552479	-------------------A--	---	---A----I-------
	Borrelia bissettii	343127751	-------------------A--	---	---T----I-------
	Borrelia crocidurae	386859673	------R---A----A-I-I-R	---	DT-N----I---E---
Other Spirochaetes (0/36)	Treponema paraluiscuniculi	338706894	---KK-----A----A--FFT-		IGAKGVK----CEN--
	Treponema saccharophilum	381181581	----K-----AI---A---MGH		LGREDL------EN--
	Treponema brennaborense	332296678	----K-----A----A---AGR		LGREDV--I--TEN--
	Treponema sp. JC4	384110038	----K-----A----A-I-AGR		LGHEEV--I--TEN--
	Treponema succinifaciens	328946938	----K-----AM--IV---AGH		LGREDL-II--TEN--
	Treponema azotonutricium	333993182	---KK-----A----A-I-AGR		QGREDM--I--SEN--
	Treponema primitia	333999780	---KK-----A----A-I-AGR		QGREDM--I--SEN--
	Treponema pallidum	15639933	---KK-----A----A--FFT-		IGAKGVK----CEN--
	Treponema vincentii	257457348	---KK-----A---IA---AG-		I-ISE-IM--CEN--
	Treponema denticola	42527896	---KK------I---A---AGT		IGHAYA--SV-CES--
	Treponema caldaria	339498653	---KK-----A----A-IFA--		LGYDDT-II--AEN--
	Turneriella parva	392405311	I--KH-RT-EA--FM--LIVE-		IT-EQPKIL---EN--
	Spirochaeta africana	383789251	I---K-----A--V-V--VSNR		LA--DFK----TEN--
	Spirochaeta thermophila	306531451	---KK-----A--V-A--IA-R		DG--SV-----AEN--
	Spirochaeta smaragdinae	302336557	---KK-----A----V---CG-		LHEGR--IV-AEN--
	Sphaerochaeta coccoides	330835991	I---K-----A--MMA--FIG-		FD-DRK--I-SE---
	Sphaerochaeta globosa	325969857	I---K-----AI---A---AGQ		IDPDLK-II-SEN--
	Sphaerochaeta pleomorpha	374316290	I---K-----AI---A--FAGQ		IDPDQKI-V-SEN--
Other Bacteria (0/>250)	Eubacterium ventriosum	154483918	---KR-QT-----Y-VSLVIN-		-CEK----K--TEN--
	Anaerostipes caccae	167748062	---KH-QT--A--Y--SLFVN-		-SEA-IK-K--TEN--
	Roseburia intestinalis	240145747	---KR-QT-----Y---LAVN-		NAE--VK-KI-TE---
	Anaero. colihominis	167770828	I---R-ET--A--Y-SSLVANR		-E-DYI--TI-S-N--
	Coprococcus catus	291521110	---KR-QT-----YIISLVVN-		-SD-YI-IKV-TEN--
	Peptoniphilus duerdenii	304439079	V--KR-TT---I-YILSLIIN-		HSD--V--IV-SSG--
	Thermomicrobium roseum	221633068	---KR-EH-AQF-Y-V--LVNR		RLP-WT--II-VEG--
	Bacillus halodurans	15616625	---KR-QT-----Y-V-LVAN-		-E-E-I-IK--AEN--
	Paenibacillus polymyxa	305860919	----R-QT--A--Y---IVANR		YSDK-I-I---AEQF-
	Meiothermus silvanus	297565962	F--K--RT-K-VEF-A---MA-		QF-GEY-----AAG--
	Thermus aquaticus	218296521	F--K--RT-KAVEY-AG-VLA-		HF-GAY-----AAG--
	Lawsonia intracellularis	94987513	M---G-QT-A---Y-SSRIL-R		IMS-SI-IQFN-----
	Bacteroides pectinophilus	218134369	---KR-QT---I-Y-VSLVVN-		--EKYM--KV-TE---
	Thermobispora bispora	296271540	-V--G-EV-EA--E--RLAVHR		RT-ERS-LM--V-G--
	Streptomyces hygroscopicus	256777844	-V--D-EV-EA--E--RLAVHR		-T-DRS-LM---AG--
	Nakamurella multipartita	258655507	-V-DH-EV--A--E--RLAAMA		-T-QRS-LM---AG--

Supplemental Figure 21: Partial sequence alignment of conserved region of Spoiiij-associtated protein showing a three amino acid insert specific for the genus *Borrelia*.

Group	Organism	Accession	134		159
Borrelia (12/12)	Borrelia hermsii	187918492	LIATKTARIKEA		GATNLAEFGLRRAQ
	Borrelia spielmanii	224534534	------S----S		--KI---L------
	Borrelia duttonii	203284534	------------		--K----L------
	Borrelia recurrentis	203288068	------------		--Q----L------
	Borrelia burgdorferi	218249472	-----------S		--KI---L------
	Borrelia garinii	219684323	-----------S		--KI---L------
	Borrelia valaisiana	224532144	-----------S		--KI---L------
	Borrelia afzelii	111115467	-----------S		--KI---L------
	Borrelia turicatae	119953414	------------		--K-----------
	Borrelia lonestari	145652231	------------		--K-----------
	Borrelia crocidurae	386859869	------------		--Q----L------
	Borrelia sp. SV1	225551865	-----------S		--KI---L------
	Borrelia bissettii	343127936	-----------S		--KI---L------
Other Spirochaetes (0/19)	Treponema brennaborense	332297432	--------VWL-	S	-KGHIM--------
	Treponema azotonutricium	333994371	--------VWL-	S	-KGSVM--------
	Treponema primitia	374813305	--------VWL-	T	-KGSVM--------
	Treponema sp. JC4	384108767	---------WL-	S	KKAPIM--------
	Treponema paraluiscuniculi	338706584	--------MWR-	S	-EGV-M--------
	Treponema succinifaciens	328947918	--------VWLS	S	KKG-VM--------
	Treponema phagedenis	320538187	---------WL-	S	NKSSIM--------
	Treponema pallidum	15639616	--------MWR-	S	-EGV-M--------
	Treponema vincentii	257457678	---------WL-	S	-KSSIM--------
	Treponema denticola	42525692	---------WL-	S	KKGSIM--------
	Treponema caldaria	339500499	--------VYL-	S	-KGTIM--------
	Spirochaeta africana	383790326	-V---A--MR--	A	AGGPIL--------
	Spirochaeta smaragdinae	302339397	--------VYN-	S	RGGLIL--------
	Sphaerochaeta coccoides	330836881	-----A--MSL-	A	SGGPIM---Y----
	Sphaerochaeta globosa	325971624	-----AS-MAL-	S	NRGS-M--------
	Sphaerochaeta pleomorpha	374317523	-----AS-MAL-	A	NRGL-M--------
Other Bacteria (0/>250)	Anaerococcus tetradius	227500763	-V----N--VR-	A	-NRLVM---A----
	Dorea longicatena	153853960	------S--VF-	A	NGDGIM--------
	Filifactor alocis	291171126	-----SS--CQV	A	KDDVVM------S-
	Blautia hansenii	260588774	-----AS-VVY-	A	KGDGIM--------
	Ruminococcus obeum	295110720	-----A--VCY-	A	RGDGIM--------
	Anaerostipes caccae	167748377	-----AS-VVY-	A	-GSGVM--------
	Coprococcus comes	226323559	-----SS--VY-	A	-D-GVM--------
	Planctomyces maris	149175181	-----S---CA-	A	-SDPVL--------
	Plesiocystis pacifica	149919902	-----A---VA-	A	EGDPVL--------
	Campylobacter jejuni	284925506	------N--VR-	A	KDSKIL---S----
	Microscilla marina	124002542	-V---A--MNL-	T	KGEPVL--------
	Chthoniobacter flavus	196229944	-----A--VCL-	A	QDDPVL--------
	Opitutus terrae	182415477	-----A---CLV	A	--DPVI--------
	Atopobium vaginae	227516151	-L------VVS-	A	QGRPVF--------
	Bacillus cereus	152974344	-----A--M-H-	A	-NDE-L---T---H
	Lactococcus lactis	125624279	-----A----SV	I	-EDP-L---T----
	Brevibacillus brevis	226310438	-----AS---NV	A	-NDV-M---T----
	Waddlia chondrophila	297620776	-----AS—CA-A	D	GD-VL---------

Supplemental Figure 22: Partial sequence alignment for the conserved region of Nicotinate phosphoribosyltransferase showing a one amino acid deletion specific for the genus *Borrelia*. Homologs for this sequence were not found in members of the *Leptospiraceae* or *Brachyspiraceae* in our BLAST search.

Group	Organism	GI	86 / 110
Borrelia (12/12)	Borrelia turicatae 91E135	119953435	DGADEILLETK A LIKGGGAAHLMEK
	Borrelia hermsii DAH	187918513	---------K- - -------------
	Borrelia recurrentis A1	203288089	-----V--DK- - -------------
	Borrelia crocidurae str. Achem	386859891	-----V--DK- - -------------
	Borrelia duttonii Ly	203284555	-----V--DK- - -------------
	Borrelia garinii PBi	51598912	---------K- S ----M-G------
	Borrelia afzelii ACA-1	216263505	---------K- S ----M-G------
	Borrelia burgdorferi 80a	195941477	---------K- S ----M-G------
	Borrelia valaisiana VS116	224531804	---------K- S ----M-G------
	Borrelia spielmanii A14S	224534514	---------K- S ----M-G------
	Borrelia bissettii DN127	343127958	---------K- S ----M-G------
	Borrelia sp. SV1	225551838	--------GK- S ----M-G------
Other Spirochaetes (0/18)	Treponema phagedenis F0421	320536897	------DSKKN --------L-R--
	Treponema vincentii ATCC 3558	257457570	------DSKKN --------L-R--
	Treponema denticola ATCC 3540	42527149	------DPDKN --------L-K--
	Treponema saccharophilum DSM 2	381179076	------DG-NN --------L-L--
	Treponema paraluiscuniculi	133712453	------DTQNF V-------L-Q--
	Treponema succinifaciens DSM 2	328949354	------DPDNN --------L-R--
	Treponema pallidum subsp. pall	15639604	------DTQNF V-------L-Q--
	Treponema brennaborense DSM 12	332298818	------SP-GY --------L-R--
	Treponema sp. JC4	384107651	-----VDPDCN ---------VR--
	Spirochaeta africana DSM 8902	383789693	----AVDPQLA ----------T--
	Leptonema illini DSM 21528	374587785	-----VDPQLR ---------TR--
	Spirochaeta thermophila	307719346	-----VDPTLH -V----G-LFR--
Other Bacteria (0/>250)	Oceanobacillus iheyensis	23100064	-----VDANNH --------LFR--
	Bacillus cereus	52142718	------DSNLQ ------G-L-R--
	Leuconostoc citreum	170016933	-----VDT-LN G-------F----
	Fulvimarina pelagi	114707094	------DGDLR ------G-L-R--
	Hoeflea phototrophica	163759649	------DGALT -V----G-L-R--
	Pelagibaca bermudensis	114766781	-----FDS-LN ------G-L-Q--
	Sagittula stellata	126729285	-----FDE-LS ------G-L-Q--
	Stappia aggregata	118588076	-----LDPHLS ------G-L-R--
	Oceanicola granulosus	89069151	-----YDSNLN ------G-L-Q--
	Ruegeria pomeroyi	56696213	-----FDGDLN ------G-L-Q--
	Ochrobactrum anthropi	153009422	-----VDTNLS ------G-L-R--
	Hirschia baltica	254294211	-----FDP-LR --------L-R--
	Picrophilus torridus	48477637	-----FDPYGN ------G-LVR--
	Haemophilus somnus	170718309	------NPQKM M-------LTR--
	Pasteurella multocida	15603535	------NPQKM M-------LTR--
	Variovorax paradoxus	239815147	------DPRGF MV------LTR--
	Delftia acidovorans	160899421	------DGRGY MV------LTR--
	Scardovia inopinata	294791458	-----VDKNFD G-------L-W--
	Haemophilus somnus	113460269	------NPQKM M-------LTR--
	Gardnerella vaginalis	283782797	--S--VDKNFN G-------L-W--
	Monosiga brevicollis	167526415	-----VSAQLD C-----GCL-Q--
	Nostoc punctiforme	186681596	-----VDPQKN ---------TR--
	Nodularia spumigena	119510229	-----VDPQKN ---------TR--
	Plasmodium chabaudi	70953348	-----VDSDLN ----R-G-LVR--

Supplemental Figure 23: A partial sequence alignment of ribose Ribose 5-phosphate isomerase showing a one amino acid insert specific for the genus *Borrelia.* Homologs for this sequence were not found in members of the *Brachyspiraceae*, *Sphaerochaeta*, *Turneriella*, or *Leptospira* in our BLAST search.

Group	Species	ID	64		94
Borrelia (12/12)	Borrelia burgdorferi	195941574	HADHITGLLGIVMLMSQS	GE	TRKEPLIIAGP
	Borrelia sp. SV1	225551828	------------------	--	-----------
	Borrelia garinii	219684248	------------------	--	-----------
	Borrelia spielmanii	224534454	-----------I------	--	-----------
	Borrelia bissettii	343128051	------------------	--	---D-------
	Borrelia afzelii	111115584	------------------	--	---D-------
	Borrelia valaisiana	224531528	-----------I------	--	---D-------
	Borrelia crocidurae	386859992	---------------A--	-D	------T-I--
	Borrelia recurrentis	203288182	---------------A--	-D	------T-I--
	Borrelia duttonii	203284649	---------------A--	-D	------T-I--
	Borrelia turicatae	119953531	---------------A--	-N	------T-I--
	Borrelia hermsii	187918610	---------------A--	-D	------T-I--
Other Spirochetes (0/19)	Treponema denticola	325473715	--------P-LL--S--V		D-E---Y-I--
	Treponema saccharophilum	381180848	----V---P--M--S--V		D-T---Y-Y--
	Treponema pallidum	15639805	--------P-LL--S--V		A-S---Y-I--
	Treponema paraluiscuniculi	338706768	--------P-LL--S--V		A-S---Y-I--
	Treponema vincentii	257457947	----V---P--L--SA-V		D-D---Y-I--
	Treponema succinifaciens	328948129	----V---P--L--S--V		D-T---Y-F--
	Treponema phagedenis	320536508	----V---P-LL--S--V		D-DD--Y-I--
	Treponema sp. JC4	384110060	----V---P--L--SA-V		D-T---Y-Y--
	Treponema azotonutricium	333994820	----V--IP-LL--S--V		D-DD--Y-I--
	Treponema primitia	333997962	----V--IP-LL--S--V		D-DD--Y-I--
	Treponema brennaborense	332297926	----V--IP-LM--SA-V		D-D---Y-Y--
	Treponema caldaria	339499904	----V---P--L--S--V		D-DD--T-I--
	Spirochaeta thermophila	386347399	----V---P--L--S--V		D-S---Y-Y--
	Spirochaeta smaragdinae	302338444	----V---P--M--S--V		D-E---T-I--
	Spirochaeta africana	383790621	----V---P--L--S--V		D-DT--Y-Y--
	Sphaerochaeta globosa	325971176	----V---P--L--S--V		D-N---T-Y--
	Sphaerochaeta pleomorpha	374314894	----V---P--L--S--V		D-DD--T-Y--
	Sphaerochaeta coccoides	330836512	----V---P--L--S--V		E-DT--Y-Y--
Other Bacteria (0/>250)	Eubacterium rectale	291524005	-----S--P-LLLT-GNA		E-T---T-I--
	Ruminococcus lactaris	197303575	-G---S--P-LLLT-GNA		D-----TLI--
	Roseburia intestinalis	257414083	-----S--P-LLLS-GNA		E-T---TMI--
	Coprococcus comes	226322516	-G---S--P-LLLT-GNA		D-----TLI--
	Dorea formicigenerans	166031523	-G---S--P-LLLT-GNA		D-T---TLI--
	Butyrivibrio crossotus	260438115	----VS--P-FLLT-GNA		D-R-D-L----
	Alkaliphilus oremlandii	158320712	-G---F-IP-LLST-GN-		Q-LD-VT-V--
	Catonella morbi	229824063	-G---F--P-FLSSR-FQ		GGDQ--TLY--
	Rhodothermus marinus	268315990	-G--FF--F-LLATLAML		N-T---VVV--
	Dokdonia donghaensis	86130530	-G--FF--M-VITTF-LL		K-TA--T-Y--
	Bulleidia extructa	283768806	----TA--P-LLLT-AKN		N-L--IR-Y--
	Lactobacillus antri	259503099	-G---F--P-LLSSR-FQ		GGN---T-Y--
	Enterococcus faecalis	257416213	-G---F--P-LLSSR-FQ		GGT---E-Y--
	Geobac. kaustophilus	56420868	-G--LF--P-LLGSR-FQ		SGET--TVF--
	Bacillus halodurans	15614276	-G---F--P-LLGSR-FQ		GGEN--FLY--
	Enterococcus faecium	257878181	-G---F--P-LISSR-FQ		GGDT--E-Y--
	Pediococcus pentosaceus	116492893	-G---F--P-FLSSR-NQ		GGS-E-T-F--
	Hyperthermus butylicus	124027542	-G--FF--P-LLQS-GML		G--T--LV---
	Ignicoccus hospitalis	156937827	-G--VL--P-LLQT-AMA		S-RDE-LVI--
	Metallosphaera sedula	146304551	----VL--PSLIQT-GMY		D---R-Y-L--
	Vibrio harveyi	153832098	-G--CY--P-LLASAGMN		N--D--T-IA-
	Providencia stuartii	188026419	-G---F--P-LLCSR-MG		GSTD--TLY--
	Pyrococcus abyssi	14521306	-G--YL--PALIQT-NLW		K-----H-Y--

Supplemental Figure 24: Partial sequence alignment of conserved region of Ribonuclease Z showing a two aa insert specific for the genus *Borrelia*. Homologs for this sequence were not found in members of the *Leptospiraceae* or *Brachyspiraceae* in our BLAST search.

Group	Species	GI	206		236
Borrelia (12/12)	Borrelia crocidurae	386859948	GCHMILPVEFANKVR	R	EPDDIKFCPYCSRIL
	Borrelia duttonii	203284608	---------------	-	---------------
	Borrelia turicatae	119953490	---------------	-	----V----------
	Borrelia hermsii	187918568	---------------	-	---------------
	Borrelia bissettii	343128011	---------------	-	--N------------
	Borrelia valaisiana	224531577	---------------	-	--N------------
	Borrelia garinii	51598965	---------------	-	--N------------
	Borrelia burgdorferi	224532401	-------I-------	-	--N------------
	Borrelia afzelii	216263491	-------I-------	-	--N------------
	Borrelia sp. SV1	225551819	-------I-------	-	--N------------
	Borrelia spielmanii	224534561	-------I-------	-	--N------------
Other Spirochaetes (0/23)	Treponema denticola	325473908	-------AQ-V-E--		SDQ------------
	Treponema primitia	374814114	-------AQ---M--		KREE-V---------
	Treponema sp. JC4	384109888	-------AQ---I--		VG-N-N---------
	Treponema pallidum	15639485	-------AQ-STG--		-GNS-VY--------
	Treponema azotonutricium	333994742	-------AQ---DI-		VGKE-V---------
	Treponema saccharophilum	381180808	-------AQ---E--		GGEK-L---------
	Treponema brennaborense	332297994	-------AQ---E-H		NG-K-V---------
	Treponema succinifaciens	328948112	-------AQ---E-H		HGEK-L---------
	Treponema vincentii	257457794	------SAQ--IE--		-GKN-MY--------
	Treponema medium	15022435	------SAQ--IE--		-GKN-MY--------
	Treponema phagedenis	320537363	-------AQ--IE-H		KGKS-MY--------
	Treponema caldaria	339500010	-------AQ---Q--		MGEE-I---------
	Spirochaeta smaragdinae	302338587	----M--AQ-E-D--		SGEN-L---------
	Spirochaeta thermophila	307718769	--F-L--R--V-RI-		RGEE-I---H-----
	Spirochaeta africana	383790694	----M--NQLV-D--		SGEH-HN--------
	Sphaerochaeta coccoides	330836586	----T---Q-V-T--		KNEE-E---------
	Sphaerochaeta globosa	325971192	---IV--IQ-V-D--		SAK--E---------
	Sphaerochaeta pleomorpha	374314883	---IV--IQ-V-D--		SQTQ-D---------
	Brachyspira intermedia	384209704	A-NVAI-KMTV-E--		RQNQ-IM-FH-G---
	Brachyspira pilosicoli	300870319	A-NVAI-KMTV-E--		RQNQ-IM-FH-G---
	Brachyspira murdochii	296125762	A-NVAI-KMTV-E--		RQNQ-IM-FH-G---
	Brachyspira hyodysenteriae	225621328	A-NVAI-KMTV-E--		RQNQ-IM-FH-G---
Other Bacteria (0/>250)	Chlamydophila abortus	62184859	---IV--PQHE-L--		KKDRLI--EH-----
	Waddlia chondrophila	297621674	---IT-TAQHE-L--		KGERLV--EH----H
	Chlamydia trachomatis	296435919	---IA-TPQHE-L--		KQDHLV--EH-----
	Haliangium ochraceum	262195662	----A--PQLN-TLA		AGATVE---R-N--V
	Desulfovibrio vulgaris	218885362	---ISI-PQSYIELQ		KGTQ-LS--N-Q-LI
	Nautilia profundicola	224373474	--N-KINDKIFSE-I		KGEE-VT--H-G-V-
	Helicobacter hepaticus	32266138	--FIK-NDTIYSEIL		KG---IN--H-G---
	Campylobacter gracilis	257461317	--F-RISDKTYSA-I		KSD--VT----G---
	Geobacter bemidjiensis	197116877	--N-H--PQLY-TLF		RAD-VIT--H-Q---
	Campylobacter hominis	154148691	--F-KINDKTYSA-V		KSD--VT----G---
	Myxococcus xanthus	108759984	--N-NV-PQLY-QL-		TGLGTDI--S-N--I
	Sorangium cellulosum	162452055	---LSV-PMMFQ-M-		RQEEFER--N-N---
	Stigmatella aurantiaca	310823613	--N-NV-PQLY-QL-		TSLGTDV--S-N--I
	Chthoniobacter flavus	196231449	----KVTTQTVVR-K		GNRE-VH-EQ-G---
	Alistipes shahii	291514606	--FNRI-PQRQVDI-		QGKK-II-E--G---
	Dyadobacter fermentans	255038890	--FS-V-PQRQADI-		-RKKLIV-EH-G---
	Microscilla marina	124004509	--FNVV-PQRQAEI-		-RKK-IV-EH-G---
	Cytophaga hutchinsonii	110638206	--FNTV-PQRQGEI-		-RKK-IV-EH-G---
	Solibacter usitatus	116619945	A--IV-RLQYFQDLK		RGES-LP-ES-Q---
	Persephonella marina	225850570	--F--I-PKVYSELV		KSKKLLT--H-G-F-
	Meiothermus ruber	291294467	A-NVQ--MHV-QQ-H		QASKVVR--S-G---

Supplemental Figure 25: A partial sequence alignment of conserved region of Hypothetical protein BDU_716 showing a one amino acid insert specific for the genus *Borrelia*. Homologs for this sequence were not found in members of the *Leptospiraceae* in our BLAST search.

```
                                                         374                                    412
Borrelia      Borrelia turicatae          119953471      EAIILSMTKKERLNPVILN S PSRKKRIALGSGTTVFEVN
(12/12)       Borrelia hermsii            187918549      ------------V------ - -------------------
              Borrelia lonestari          145652229      ----F-------------- - -------S------I----
              Borrelia spielmanii         224534518      ------------I------ N ----------------D--
              Borrelia bissettii          343127994      ------------I------ N ----------------D--
              Borrelia valaisiana         224532274      ------------I------ N --------------I-D--
              Borrelia garinii            386854096      ------------I------ N --------------I-D--
              Borrelia sp. SV1            225551839      ------------I------ N --------M-------D--
              Borrelia burgdorferi        365992395      ------------I------ N --------M-------D--
              Borrelia duttonii           203284591      --------QR--R------ - --------------I----
              Borrelia afzelii            216263608      ------------T------ N --------------I-D--
              Borrelia crocidurae         386859927      --------QR--R------ - --------------I----
              Borrelia recurrentis        203288125      --------QR--R------ - S-------------I----
Other         Spirochaeta thermophila     386346818      --------PA--R--R-IG   --------R----S-Y---
Spirochetes   Spirochaeta smaragdinae     302338385      --------IV--R-HR-IG   ---R----K---SS-A---
(0/23)        Sphaerochaeta coccoides     330836478      K---Q---YT--E-CH-IG   -T-R----R---SS-ND--
              Treponema brennaborense     332297535      ------------A-HL-IG   -G-R----K----S-A---
              Treponema sp. JC4           384107750      ----Q---Y-----HL-IG   ---R----K----S-AD--
              Brachyspira hyodysenteriae  225620952      K---Q--------ALFPVI   N---M--SK---QS-YD--
              Brachyspira intermedia      384210112      K---Q--------ALFPVI   N---M--SK---QS-YD--
Other         Buchnera aphidicola         21672654       ----Y---H---MH-I-IK   G---R--------KIQD--
Bacteria      Thermocrinis albus          289548587      ---------E--K--A-I-   L---Q---R------S---
(0/>250)      Aquifex aeolicus            15606801       ----N---PE--R--K-I-   M-------R------SD--
              Buchnera aphidicola         21672654       ----Y---H---MH-I-IK   G---R--------KIQD--
              Veillonella parvula         282850086      ----T---AA--E--S---   G--R---------Q-QD--
              Filifactor alocis           291171833      ----R---L---R--D-I-   A--R----S----SIA---
              Eubacterium yurii           304551351      ----Q----Q---D-S-I-   G---I---K----Q-SQ--
              Ruminococcus obeum          295109860       -------P---S--G---   ----N---R-A-VDIT---
              Coprococcus comes           226325149      ----Y---I---Q--D-IT   PQ--R---A-A-VDIS---
              Brevibacillus brevis        226313205      ---AK----Q--A--DL--   A--R----S----SIQ---
              Meiothermus ruber           291295393      ---V----P---RD-R---   A--R----A------Q---
              Thermus aquaticus           218295520      ---V----PE--KD-R---   G--R----K----S-Q---
              Rickettsia bellii           157826674      --------P---R--D-I-   A--R------A----QK--
              Starkeya novella            298292734      K---D-------R--KL-D   G--R----A----K-ED--
              Maricaulis maris            114571433      ---------Q--AK-E--K   A-------A---VQ-AD--
              Acidiphilium cryptum        148261145       ---S----A--A--D-MK   A-----V-A----S-Q---
              Kingella oralis             238021560      ----N---P---A--A-IK   A-------A-A----Q---
              Eikenella corrodens         225025490      ----N---P---A--AL-K   A---R---A-A----Q---
              Kosmotoga olearia           239617834      ----S---RE--R--K-I-   Y---Q---R----S-T---
              Fusobacterium varium        253581989      ----Q----E--KK-D--K   A------------E-AD--
```

Supplemental Figure 26: A partial sequence alignment of conserved region of Signal recognition particle, subunit FFH/SRP54 showing a one amino acid insertion specific for the genus *Borrelia*. Homologs for this sequence were not found in members of the *Leptospiraceae* in our BLAST search.

Group	Species	GI	52		97
Borrelia (12/12)	Borrelia burgdorferi	15594416	GGMPSSHSSTVTALSTSIALTEGID		TNFIIALAFALITIRDSFGVR
	Borrelia sp. SV1	225552245	-------------------------		---------------------
	Borrelia bissettii	343127392	------------------------N		---------------------
	Borrelia valaisiana	224532242	------------------------G		---------------------
	Borrelia garinii	386853478	------------------------G		---------------------
	Borrelia spielmanii	224534874	-----------------------VG		---------------------
	Borrelia afzelii	111114892	--------------A---------G		---------------------
	Borrelia turicatae	119952873	--------------A---LI-----		---------------------
	Borrelia hermsii	187917949	--------------A---LI----N		---------------------
	Borrelia duttonii	203283996	--------------A---LIK---N		-H-------------------
Other Spirochaetes (0/19)	Spirochaeta africana	383790271	---------M--S-AL-TG--Y-FN	T	GL-MF-FFYGGLVV--AM---
	Spirochaeta thermophila	386346208	--------AL----A---GFHD-A-	S	SL-FLSVFY-A-I---AV---
	Sphaerochaeta globosa	325971017	-------TAG-I--V------Q--G	T	VY-A--AT--AVV-H-AM-I-
	Sphaerochaeta pleomorpha	374315103	-S-----TAG-I--L-G-GM----G	T	VD-A-SMT--A-V-H-AM---
	Treponema caldaria	339499894	---------L----A--V-FK---G	S	-L--VT-CL---V----M---
	Treponema brennaborense	332298018	--------ALMCT-C---GFRS---	S	DI--LSFC---VV---AV---
	Treponema sp. JC4	384109473	--------AL--S-CVT-GFRH---	S	DI-VFS-M-FFVV---A----
	Treponema primitia	333998644	-------A-M-S-MT----II--VR	S	NL-AVSFFMS--VM--AM---
	Treponema phagedenis	320536264	--------AL-SS-A---GIK----	S	-I--F-FFSSI-V---AL---
	Treponema vincentii	257456862	--------AL------T-GFKQ-VS	S	DL--FSIFS-M-V---AM---
	Treponema denticola	42526896	--------AL-AS-TV--GIRQ-F-	S	DL--F-CFM---V---AV---
	Treponema saccharophilum	381180874	--------AV-SCVA-CVG-RS-L-	S	DV--VSFVLFF-----AL---
	Treponema succinifaciens	328948214	-S------AL-AT-C-T-GFRS-VN	S	DV--LS-G-Y-V----AV---
	Treponema azotonutricium	333994927	-------AAV-CSMA-AVGVYD--G	S	NL-AVCFF--MVAM--AM---
	Treponema pallidum	15639146	--------AL-S--TL-F--KC-LH	S	DL--FSFFS-I-VV--AL---
	Treponema paraluiscuniculi	338706131	--------AL-S--TL-F--KC-LH	S	DL--FSFFS-I-VV--AL---
Other Bacteria (0/>250)	Turicibacter sanguinis	293375096	--------AF-S--A-AVGV-D--H	S	TT-A-SFC--AVV-F-AM-I-
	Clostridium leptum	160932238	--------AF-CSIAMG-GFR--FA	S	SF-AL-FMI-MVVMY-AM---
	Catonella morbi	229824325	--------AA-SS-I--LV-EY-FE	S	PLVA--TI-GV-VMF--M---
	Ammonifex degensii	260892548	-----A--AM-S--AVAVG-CL-F-	S	AE-AM--V----VWH-AM---
	Halothermothrix orenii	220932274	-------A-F-ST---TVG-NY-F-	S	DL-A-VTV-S--I-Y-AG---
	Eubacterium siraeum	167750991	-----A--A--C-TAITTCRVC--C	S	PE-AL-MIL-MVVMY-AM---
	Filifactor alocis	291171532	---------F-IG-AN-VG----Y-	S	TI-ALS-V---VVMY-AA---
	Bacillus halodurans	15615964	--------AA----A-A---ED-L-	S	PL-A-SAV-GI-VMF-AT---
	Listeria monocytogenes	16804424	--------AA----M-TL-IEY-L-	S	PY-A-SVV-GI-VMF-AT---
	Brevibacillus brevis	226314334	---------A------AVG-R--FG	S	-M-A-SAILGV-VMF-AA---
	Lactococcus lactis	116511824	---------L-V--A-ATG-RQ-FE	S	PL-A--TVL-FVVLY-AQ-I-
	Abiotrophia defectiva	229825745	--------A---SVALMTG--T-F-	S	P -G--FIL-IVVMH-AS---
	Geobacillus kaustophilus	56419884	--------AG-S--A-F---ER-VR	-	ID-AL-AL-G--VMY-AQ---
	Lactococcus lactis	281491493	---------L-V--A-ATG-IQ-F-	S	SL-A--TVL-FVVLY-AQ-I-
	Oceanobacillus iheyensis	23099802	--------AA----T-A-GI---VT	S	SV-AL-CI-SV--MF-AS---
	Bacillus mycoides	228998316	--------------A-GVGMV---T	S	AV-A--VI--I-VMY-AS---
	Exiguobacterium sibiricum	172058214	-----------V--AVV-GFQ--FS	S	SL-AL-AI--V-IMY-AT---
	Deinococcus geothermalis	94984807	--------AM-A--T-GV---Q-MG	S	PL-AASAV----VMY-AT---
	Thermus thermophilus	46199060	-----T--A--S--AV-VG-R--F-	-	SL-AV-AV----VMY-AA-I-
	Meiothermus ruber	291296743	--------A--A--A-GVGI---VG	S	AF-A--VVL-I-VMY-AT-I-
	Crocosphaera watsonii	67924906	-----A--AL-G--A-GVG--K-WE	S	PE-A--CL--V-VMY-AA---
	Microcystis aeruginosa	166364197	-----A--AL-G--A-GVG-QM-WS	S	PE-A--AL--V-VMY-AA---
	Fusobacterium ulcerans	257467716	-----------SC-T-C--IRY--R	S	DI-A-TII-SG-VMY--A-I-
	Arthrospira maxima	209526716	--------AF-G--AA-VGQVI-WG	S	PE-A--VV--I-VMY-AA---
	Raphidiopsis brookii	282895576	-----A--AL----AAGVGQ-I-WS	S	PD-AL-AVV-I-VMY-AT---
	Nostoc punctiforme	186683903	-----A--AL----AAGVGQ-L-WA	S	PD-AV-MI--I-VMY-AA---
	Nodularia spumigena	119509102	-----A--AL----AAGVGQSL-WA	S	PD-AL-TV--I-VMY-AA---
	Anabaena variabilis	75908917	-----A--AL--S-AAGVGQ-L-WA	S	PD-AL-TV--I-VMY-AA---
	Prochlorococcus marinus	123966394	--------ALI-GATSG-G-QL-F-	S	PI-AL-I-IS--VMY-AS---

Supplemental Figure 27: A partial sequence alignment for conserved region of Hypothetical protein BSV1_0075 showing a one amino acid deletion specific for the genus *Borrelia*. Homologs for this sequence were not found in members of the *Leptospiraceae* or *Brachyspiraceae* in our BLAST search.

Group	Organism	ID	364		402
Borrelia (12/12)	Borrelia turicatae	119953137	LSEGYDLQYYVKACKIVE	N	VLIPKFNEIFNNYSYIITP
	Borrelia recurrentis	203287796	-------E--T-----I-	-	------D------------
	Borrelia duttonii	203284259	-------E--T-----I-	-	------D------------
	Borrelia hermsii	187918212	----------A-----I-	-	--V---S-----C------
	Borrelia crocidurae	386859575	-------E--T-----I-	-	------D------------
	Borrelia bissettii	343127657	------VK--S---E-LQ	-	-I-----KL-ESCDF----
	Borrelia garinii	219684617	------AK------E-LQ	-	LI-----KL-ESCDF----
	Borrelia valaisiana	224531998	------AK--A---E-LQ	-	LI-----KL-ESCDF----
	Borrelia spielmanii	224534264	------AK--A---E-LQ	-	LI-----KL-ESCDF----
	Borrelia afzelii	216264000	------SK--A---E-LQ	-	LI-----KL-ESCDF----
	Borrelia burgdorferi	221217739	------SK--A---E-LQ	-	LI-----KL-ESCDF----
	Borrelia sp. SV1	225552009	------SK--A---E-LQ	-	LI-----KL-ESCDF----
Other Bacteria (0/>250)	Brevibacillus brevis	226310281	--S--YDA--K--QQVRT		LI-QD--S--AD-DV-LH-
	Paenibacillus polymyxa	305859990	--S--YDA--L--Q-VRT		LIKQD-DRV-EQ-DV--G-
	Lactobacillus plantarum	28377926	--A--YDA-FH--GQVRT		MI-ND--KV-ED-DL-MG-
	Bacillus coahuilensis	205372469	--S-FYDA--K--QQART		LIKKD-EDV-EK-DV--G-
	Pediococcus acidilactici	270291085	--A-SYDAFFK--AQVRT		LI-DD--KV-E--DL--G-
	Eubacterium siraeum	291531793	--S--YDA--L--LALKQ		QI-KEY----E-ADV-L--
	Acetohalobium arabaticum	302392790	--S--YDA--L--Q-VRT		LIKQD-E-A-AD-DVL-S-
	Desulfotomaculum reducens	134300180	--A--YDA--L--L-VRT		LIKQD-DRA-EK-DALLS-
	Coprococcus catus	291520975	--S--YDA--I--LQVKA		LIKKG-DDA-AK-DI-LG-
	Gemmata obscuriglobus	168704720	--S--KDA--L--L-VRR		LVKKD-D-A-KTCDVVMG-
	Geobacter sulfurreducens	298507414	--S--YDA--L--Q-VRT		LIMQD-MKA-EQVDALL--
	Pelobacter propionicus	118581491	--S--YDA--L--Q-VRT		LIMAD-IQA-EGVDL-L--
	Ignisphaera aggregans	305663461	--I--REM--IR-LRFRR		M-RD------KRFDAVVS-
	Sulfolobus islandicus	229584745	--A--YEEF-L--L-VRN		LIKKSLD-L-TK-DILVS-
	Dictyoglomus turgidum	217967553	--A--YDA--L--T-VRR		LIRME-E-A-KKVDI-AS-
	Acetobacter pasteurianus	258543589	--A--YDA--L--Q-VRT		LIKRD-EQA-E-VDVLL--
	Acidiphilium cryptum	148259882	--A--YDA--L--Q-VRA		LILRD-Q-A-GKVDALL--
	Methanocella paludicola	282165110	-----YGK--L--L-VRT		LIKQD-EKV-KGVDVLAA-
	Chlorobium tepidum	21673107	--A--YDT--K--QQVRR		-FQD-YR-A-EKVDV-FG-
	Chlorobium limicola	189345875	--A--YDT--K--QQVRR		-FQDRYR-ALEKVDV-AG-
	Chitinophaga pinensis	256420379	--A--YDA-FA--QQVRR		MVVD-L---LSE-DA-VM-
	Pedobacter heparinus	255533716	--A--YDA--Q--QQVRR		LIRE-ME-LLTRFDV--S-
	Aquifex aeolicus	15605793	--A--YDA--L--Q-VRR		LITND-LKA-EEVDV-AS-
	Persephonella marina	225851021	--S--YDA--L--Q-VRT		LIYQD-MKA-ESVDI-L--
	Gardnerella vaginalis	298253223	--A--YDAW-GS-Q-VRT		LI-RD-E-A-KKVDVL-S-

Supplemental Figure 28: Partial sequence alignment of Aspartyl/glutamyl-tRNA amidotransferase subunit A showing a one amino acid insertion specific for the genus *Borrelia.*

Group	Organism	ID	15		48
Borrelia (12/12)	Borrelia duttonii	203284234	EGYLARSVYKLIEIDKKFSLFS		SGNILDIGASPG
	Borrelia crocidurae	386859549	----------------------		------------
	Borrelia hermsii	187918187	---------------QR-----		------------
	Borrelia turicatae	119953113	----------------R-----		------V-----
	Borrelia bissettii	343127630	--------------NE------		---V--------
	Borrelia garinii	51598573	--------------NE------		---V--------
	Borrelia burgdorferi	387827228	--------------NE------		---V--------
	Borrelia sp. SV1	225552371	--------------NE------		---V--------
	Borrelia valaisiana	224532294	--------------NE------		---V-----A--
	Borrelia spielmanii	224534384	--------------NE------		Y--V--------
	Borrelia afzelii	111115138	--------------NE------		Y--V--------
Other Spirochetes (0/36)	Treponema phagedenis	320535785	---P-------E--N---K---	P	-DK---L--A--
	Treponema azotonutricium	333995984	---P-------K-M-E----LK	R	GMKV--L--A--
	Treponema saccharophilum	381178935	---P-------K---E--GMIK	K	GYTV--L--A--
	Treponema pallidum	15639669	A--R-------AAL---Y--L-	R	ASRV--L--A--
	Treponema vincentii	257456100	---P-------E-MQ---N--G	K	TDSV--L--A--
	Treponema denticola	42527473	-N-P-------E-MN---N---	P	NDKV--L--A--
	Treponema succinifaciens	328949039	---P-------Q---E--GMLK	K	-SI---L--A--
	Treponema brennaborense	332297375	---P-------K---E--GMIR	K	NYRV--L-----
	Treponema sp. JC4	384108523	---P-------K---E--GMIK	K	NYTV--L-SA--
	Spirochaeta thermophila	307719842	---P-------M--QQ-YRIV-	P	GDRV--L--A--
	Spirochaeta africana	383789324	Q--F-------E--NN--G-LK	R	--RY--M--A--
	Spirochaeta smaragdinae	302336841	---P---I---E--E-R-GVIN	T	ELP---V--A--
	Sphaerochaeta globosa	325972772	---P-------E-LQQS-N-IK	S	GDTV--V--A--
	Sphaerochaeta coccoides	330836075	---P-------E--Q-V-RIIR	P	GAKV--V--A--
	Sphaerochaeta pleomorpha	374316396	---P-------E--QNT-K-VK	P	GDSV--V--A--
	Brachyspira pilosicoli	300869923	-N-K----F--E-AQN--KFIK	S	-D-V--V-C---
	Brachyspira hyodysenteriae	225619180	-N-K----F--E-AQN--KFIK	A	-D-V--V-C---
	Brachyspira murdochii	296127776	-N-K----F--E-AQN--KFIK	S	-DTV--V-C---
	Brachyspira intermedia	384208684	-N-K----F--E-AQN--KFIK	A	-DTV--V-C---
	Leptospira weilii	489091975	E-VS-GA---LKAFEV-PFQV	D	-KLCV-L---T-
	Leptospira alstoni	463329184	E-VS-GA---LKVFDV-P-KV	D	-KLCV-L---T-
	Leptospira inadai	498101585	K-VS-GAF--K-ALT--NISV	D	-KLCI-W---T-
Other Bacteria (0/>250)	Sorangium cellulosum	162450262	Q--P----F--E---RRVR-LR	P	GQRV--L--A--
	Desulfovibrio piger	212704983	-N-P-------K-L-A--K--R	Q	GMRV--L--A--
	Legionella drancourtii	254496115	---RS-A----K-V-E-E--LK	P	GMTVV-L--A--
	Acinetobacter baumannii	126642713	---R--AA---L--QE-YK-IK	P	GMTVV-L--A--
	Zymomonas mobilis	56551298	---RS-AAF----L-ER---LK	N	ARR-I-L-IA--
	Maricaulis maris	114569722	---RS-AA---LQL-ER-K-LK	P	GMRVV-L-SA--
	Oceanicola granulosus	89068308	D--RG-AA--IL-L-D-YRFLV	P	GARVV-L-CA--
	Sagittula stellata	126729852	---RG-AAF-IL---E-YRFLV	P	GARVV-L-CA--
	Methylobacterium populi	188582866	---RS-AAF------ER-K-LK	P	AQR-V-L--A--
	Stappia aggregata	118589174	D--RS-AA-------D-HK-LK	P	GYRVV-L-CA--
	Neorickettsia sennetsu	88608723	-Q-RS-AA---L---E--N-IR	K	GFVV-EL-SA--
	Caulobacter segnis	295689972	L--RS-AAF-IS---E--HF-R	K	GARVI-L-CA--
	Methanosaeta thermophila	116754447	---R---A---KQ-ND--HIIR	R	GSRVV-L--A--
	Cryptosporidium hominis	67608071	--FR---A----Q--E-YNI-D	K	VTRAV-LC-A--
	Plasmodium berghei strain	68074303	A--R---AF---Q-AR-YNI-K	N	ANILI-LC-A--
	Halalkalicoccus jeotgali	300709773	Q--RS-AA---QQL-EMED-L-	H	GDRVV-L--A--

Supplemental Figure 29: Partial sequence alignment of conserved region of Ribosomal RNA methyltransferase showing a one amino acid deletion specific for the genus *Borrelia*.

Group	Species	ID	320	Insertion (boxed)	365
Borrelia 12/12	Borrelia spielmanii	224534310	GIDIANLANTPIRAAREGIVVTVGFNAG	G	YGKYIVISHSNGFQTLY
	Borrelia garinii	219685977	------------K---------------	-	-----I-----------
	Borrelia afzelii	111115087	------------K---------------	-	-----------------
	Borrelia valaisiana	224531978	------------K-S-------------	-	-----------------
	Borrelia bissettii	343127580	------------K-S-------------	-	-----------------
	Borrelia sp. SV1	225552006	------------K-S-------A-----	-	-----------------
	Borrelia burgdorferi	387827178	------------K-S---V---A-----	-	-----------------
	Borrelia hermsii	187918136	------A-----F-TK---------SV-	-	---------N-------
	Borrelia turicatae	119953061	------V-----F-TK------A--SV-	-	-----I---N-------
	Borrelia crocidurae	386859491	------V----VV-TK------A--SV-	-	-------A-N-------
	Borrelia duttonii	203284183	------V----VV-TK------A--SV-	-	-------A-N-------
	Borrelia recurrentis	203287722	------V----VV-TK------A--SV-	-	-------A-N-------
Other Spirochaetes (0/36)	Turneriella parva	392401646	-V-L-DATG---Y--AD-R-IGC-YSG-		--LAVK---PF------
	Treponema azotonutricium	333995248	AV-L-APIG-LV---SD-K-S-----ST		F--F-VLG-A-S---M-
	Treponema pallidum	15639148	-L-MVSRRG--VYS-LG---R---YS-V		--N-LIVG-HA-Y----
	Treponema denticola	42527820	---L-TYRGA--Y--LP-TIAAT-YSNV		--N-VI-R-HS-Y----
	Treponema vincentii	257456803	---M-APKG-SVY--LN-Q-IAT-YSTV		--N-VM-R-HS-Y----
	Treponema primitia	374813996	AL-L-AGLG--VK-SLD-K-A---L-SV		-----IL--G-------
	Treponema sp. JC4	384108522	---M-TGSG---Y--LD-K-T-T----T		--N-VI-T-HS-YK---
	Treponema succinifaciens	328949038	---M-CAKG---Y--LP-V-SVC-D--I		----V-V--HS-YK---
	Treponema brennaborense	332298566	---M-IAQG------MS-KIIAA-YTNV		--N-V--D-E--Y----
	Treponema caldaria	339499484	-L-L-APLG--VY-C-----SNT-Y-EV		--N----A-DG-WTS--
	Spirochaeta africana	373483417	------APG-R-N-S-G-R-AVA-SHPT		--N-V----DG------
	Spirochaeta smaragdinae	302338245	---L-VPEG--VMP--S---EQT-YDSV		L-N-VIL--EG-YE-V-
	Spirochaeta thermophila	307718438	----PGDVG--VY--LG-R--QT-THPI		----VIL--PD------
	Leptospira noguchii	359722224	-V-F-SAEG---Y-TAP----ES-QSS-		-L--N-K-N-L--IF-V-
	Leptospira weilii	359726098	-V-F-SAEG---Y-TAP----ES-QSS-		-L--N-R-N-L--IF-V-
	Leptospira interrogans	45659184	-L-M-AEEG--VY-SAD-E-YFSDKKG-		--NL-ILG-KL-YE---
	Leptospira borgpetersenii	116327063	-L---AEEG--VY-SAD-E-YFSDKKG-		--NL--LG-KL-YE---
	Leptonema illini	374585366	-L-M-GARG--VE-TA--E----MNWDA	-	--NAV--K-GF------
	Brachyspira murdochii	296125657	-V--PGRL---VY---K-K-IFA-YSG-		--NLVIVR-DK-YT-Y-
Other Bacteria (0/>250)	Brevibacillus brevis	226314868	-V-M-ARKG---L--AD---LFA-Y-G-		F-NTVM-K-NAEYT---
	Enterococcus gallinarum	257868949	---FTGSSG---Y-IQG-K--EA-YGPS		T-N-VI-K-P--IYSY-
	Clostridium hylemonae	225570772	-M-F-AATG---Y--AA-T--SA-YSGN		A-NL-I-N-G--L--Y-
	Alkaliphilus oremlandii	158321812	-----SSKG-TVT--DA-R-SFA-RQGS		--NLVI-D-E--Y--Y-
	Dorea formicigenerans	166032791	-V-F-ASTG---Y--AA-T-TSA-YSGK		A-NL-I-N-G--LL-Y-
	Eubacterium yurii	306820490	-V-F-DS-G---Y--E--Q-IYA-PKGT		--NAVM-D-GA-IV---
	Herb. seropedicae	300309747	-V-F-APTG---H--AD-V-DF--KQN-		--NIV--K-WS-YS-A-
	Bordetella avium	187479434	-V-Y-APSG---HSTAD-T-DF--WQN-		--NVVI-K-HGQYS---
	Meiothermus silvanus	297564670	-L-L-APPG--VY--AS-T--SA-W-GV		F-QAVE-D-GY-YR---
	Campylobacter showae	255321542	-V-YGAPKG---K--G--T-KF--TKS-		---VVILG-TS-YE---
	Lawsonia intracellularis	94986820	-L-L-ST--A--P--ND----YT-DLGI		--NIVI-D-GL-L----
	Wolbachia endosymbiont	42520351	-V-Y-AKLG---Y--A--VIEYI-K-G-		--N--K-K-K-EYS-C-
	Brucella ovis	148559209	-V-W-APRG---I--GN-V-EKA-WSN-		--NQTL-R-A--YV-S-
	Maricaulis maris	114569207	-T-F-APRG--VM--GN-V-ERADRYGS		F-N-VR-R-A--Y--A-
	Fulvimarina pelagi	114706933	-V-W-APRG---L--AG-T-LEA-WKS-		--RH-K-K-A--YV-S-
	Cellvibrio japonicus	192359426	-T-Y-AARG-----TGD-K-SFA-RKG-		--NC-I-N-GS-YE---
	Selenomonas noxia	292670319	----GGDYGD--Y--QA-T-EYA-WIS-		--NAVI-N-GG-IS---

Supplemental Figure 30: Partial sequence alignment of LysM domain/M23/M37 peptidase domain protein showing a one amino acid insertion specific for the genus *Borrelia*.
